# Mapping the evidence of novel plant-based foods: a systematic review of nutritional, health, and environmental impacts in high-income countries

**DOI:** 10.1093/nutrit/nuae031

**Published:** 2024-04-25

**Authors:** Sarah Nájera Espinosa, Genevieve Hadida, Anne Jelmar Sietsma, Carmelia Alae-Carew, Grace Turner, Rosemary Green, Silvia Pastorino, Roberto Picetti, Pauline Scheelbeek

**Affiliations:** Department of Population Health, London School of Hygiene and Tropical Medicine, London, United Kingdom; Department of Population Health, London School of Hygiene and Tropical Medicine, London, United Kingdom; Priestley International Centre for Climate, University of Leeds, Leeds, United Kingdom; Department of Population Health, London School of Hygiene and Tropical Medicine, London, United Kingdom; Department of Public Health, Environment and Society, London School of Hygiene and Tropical Medicine, London, United Kingdom; Department of Population Health, London School of Hygiene and Tropical Medicine, London, United Kingdom; Centre on Climate Change and Planetary Health, London School of Hygiene & Tropical Medicine, London, United Kingdom; Department of Population Health, London School of Hygiene and Tropical Medicine, London, United Kingdom; Department of Population Health, London School of Hygiene and Tropical Medicine, London, United Kingdom; Centre on Climate Change and Planetary Health, London School of Hygiene & Tropical Medicine, London, United Kingdom; Department of Population Health, London School of Hygiene and Tropical Medicine, London, United Kingdom; Centre on Climate Change and Planetary Health, London School of Hygiene & Tropical Medicine, London, United Kingdom

**Keywords:** climate change, climate change mitigation, dairy substitutes, diet change, environmental sustainability, health, meat substitutes, novel plant-based foods, plant-based, sustainable diets

## Abstract

**Context:**

Shifting from current dietary patterns to diets rich in plant-based (PB) foods and lower in animal-based foods (ABFs) is generally regarded as a suitable strategy to improve nutritional health and reduce environmental impacts. Despite the recent growth in supply of and demand for novel plant-based foods (NPBFs), a comprehensive overview is lacking.

**Objectives:**

This review provides a synthesis of available evidence, highlights challenges, and informs public health and environmental strategies for purposeful political decision-making by systematically searching, analyzing, and summarizing the available literature.

**Data Sources:**

Five peer-reviewed databases and grey literature sources were rigorously searched for publications.

**Data Extraction:**

Study characteristics meeting the inclusion criteria regarding NPBF nutrient composition and health and environmental outcomes in high-income countries were extracted.

**Data analysis:**

Fifty-seven peer-reviewed and 36 grey literature sources were identified; these were published in 2016–2022. NPBFs typically have substantially lower environmental impacts than ABFs, but the nutritional contents are complex and vary considerably across brands, product type, and main primary ingredient. In the limited evidence on the health impacts, shifts from ABFs to PB meats were associated with positive health outcomes. However, results were mixed for PB drinks, with links to micronutrient deficiencies.

**Conclusion:**

If carefully selected, certain NPBFs have the potential to be healthier and nutrient-rich alternatives to ABFs and typically have smaller environmental footprints. More disaggregated categorization of various types of NPBFs would be a helpful step in guiding consumers and key stakeholders to make informed decisions. To enable informed policymaking on the inclusion of NPBFs in dietary transitions as part of a wider net-zero and health strategy, future priorities should include nutritional food standards, labelling, and subdivisions or categorizations of NPBFs, as well as short- and long-term health studies evaluating dietary shifts from ABFs to NPBFs and standardized environmental impact assessments, ideally from independent funders.

## INTRODUCTION

The fragile interconnection between food systems and the environment is increasingly evident.^1–3^ While current agricultural practices are damaging the environment, environmental change is putting food supplies at risk of disruption if timely adaptation strategies are not used.^4–8^ This relationship exists at a time when food systems are already struggling to provide healthy diets for all, with many populations experiencing a coexistence of undernutrition and obesity.^1^^,^^3^

Structural changes in food systems are critical to both safeguard people’s health and accomplish the climate adaptation and mitigation commitments mentioned in The United Nations Framework Convention on Climate Change^9^ and the United Nations’ Sustainable Development Goals.^10^ While production-side strategies can contribute toward climate mitigation, substantial opportunities for further emission reductions and acceleration toward net-zero targets can be achieved through dietary changes and the resulting lower demand for foods with a large environmental footprint.

In food-secure and high-income settings, a shift from “conventional diets” (which typically contain high amounts of animal-based foods [ABFs]) to predominantly plant-based (PB) diets could improve population and planetary health.^2^^,^^11^ Dietary change has many obstacles, with diets influenced by many factors^12^^,^^13^ that act as barriers to increasing consumption of minimally processed PB foods (eg, legumes, vegetables). If common barriers are removed, such as the need for additional cooking skills, major changes in taste and appearance of commonly consumed dishes, and fear of social stigma,^14^^,^^15^ novel plant-based foods (NPBFs), products designed to mimic and replace ABFs to allow easy incorporation into habitual diets (eg, vegan and vegetarian meat and dairy) (see Box 1), may offer an easier option to facilitate this shift.

In recent years, the NPBF landscape has expanded rapidly. Several new types of NPBFs (eg, PB drinks, yogurts, eggs, meats) were introduced to the market, and trends showed increasing sales, volume, and investment growth across many countries.^16–21^ In 2023, data suggested a possible slowdown, especially for PB meats, with some consumers criticizing their cost and taste,^22^ and some NPBF manufacturers reporting net losses.^23^^,^^24^ However, sales of supermarkets’ own-label PB meat alternatives have seen growth,^23^ alongside consistent increases in sales of PB dairy and eggs^25^ (see Supplementary file 1, section 1.1, in the Supporting Information online for detailed information on costs).

According to a global survey focusing on individuals following vegan or vegetarian diets most or all of the time, 22.0% of consumers reported adhering to a meat-free diet, and there is growing interest in embracing PB eating, with approximately 42.0% of consumers anticipating that PB foods will replace most meat within a decade.^26^ With consumption of NPBFs in the United Kingdom doubling between 2008 and 2019, particularly among women and younger generations, and the fact that in 2022, 60.0% of US households purchased at least 1 type of NPBF, verification of any health and sustainability claims in marketed products is of vital importance.^22^^,^^27^^,^^28^ Currently, various NPBFs are advertised as potential dietary “game changers,” with claims that they would play an important and positive role in sustainability and health,^29^^,^^30^ and, thus, could play a pivotal role in the so-called consumption corridors.^31^ However, because of their novelty, some consumers question these positive claims.^32^ Although NPBFs are generally regarded as a low-carbon alternative to ABFs, their nutrient and health profiles remain largely unknown and are often criticized. This is primarily related to concerns regarding micronutrient and protein content, along with higher content of saturated fats and sodium in comparison to ABFs, and level of processing.^33^^,^^34^

Previous reviews have primarily focused on single aspects of NPBFs^17^^,^^19^^,^^22^^,^^25^^,^^29^^,^^34–46^ or ingredients of NPBFs^39^^,^^47–50^; a few recent reviews explored the positive health and environmental outcomes of consuming selected NPBFs.^51–53^ However, research quantifying the potential impacts of NPBFs is still in its infancy, and an overview that is both systematic and comprehensive, comprising health, nutrient, and environmental outcomes from peer-reviewed and grey literature of different types of NPBFs, does not yet exist, to our knowledge. This lack makes it difficult for policy makers and consumers to assess the trade-offs between nutrient composition and the environmental and health impacts of NPBFs, and hinders the potential inclusion of NPBFs in sustainable and healthy dietary recommendations.

To synthesize available evidence, highlight challenges, inform public health and environmental strategies, and inform purposeful political decision-making, we aimed, in this study, to systematically search, analyze, and summarize the available grey and peer-reviewed literature on the nutrient composition, environmental footprints, and health effects of NPBFs sold and consumed in high-income countries, and to quantify and summarize their reported results.

## METHODS

The full-study protocol we followed is published elsewhere (see Nájera Espinosa et al^54^ and Supplementary file 1, section 2, in the Supporting Information online for more details on the methods). Briefly, a systematic search was performed to identify peer-reviewed journal articles and grey literature that contained data on the nutrient composition, health impacts, and environmental impacts of NPBFs. The Preferred Reporting Items for Systematic Reviews and Meta-Analyses (PRISMA) guidelines were followed.^55^

### Peer-reviewed literature

Five scientific databases were systematically searched (MEDLINE, Embase, Global Health, GreenFILE, and the Web of Science Core Collection) on August 29, 2021; we conducted an updated search on June 29, 2022. The search was limited to articles published and accepted after January 2016 until June 29, 2022, because of the substantial growth in supply and demand of NPBFs in the past 7 years.^16–19^ In addition to database searching, citation lists from identified systematic literature reviews were handsearched (see Supplementary file 1, section 2.6, in the Supporting Information online for the full search strategy). After the quality criteria were applied (described in Supplementary file 1, Table S1 in the Supporting Information online), titles were manually and triple screened. Abstracts were manually double screened after application of a supervised machine-learning algorithm (ie, a support vector machine^56^) through Scikit Learn^57^ that ranked and highlighted likely relevant articles (ie, conducted priority screening). This approach is described elsewhere in detail (see Supplementary file 1, section 2.1, in the Supporting Information online).^58^ Full texts were manually screened by 2 authors and data were also double extracted.

### Grey literature

To capture grey literature in a systematic way, a manual search was conducted on Google (see Supplementary file 1, section 2.3, and Table S3 in the Supporting Information online). Text from the webpages was then scraped and a state-of-the-art, pretrained language model from Hugging Face^59^ was used to create a summary of each web link. Results were exported into a comma-separated value, or CSV, file. Additionally, a manual search in Google of relevant websites from the top NPBF producers in the United Kingdom and United States was conducted.^60–63^ And literature from relevant websites that promote NPBFs, such as the Good Food Institute and Green Queen, were searched and screened manually (see Supplementary file 1, section 2.3, and Tables S4 and S5 in the Supporting Information online).

### Data analysis, categorization, and key definitions: nutrient, health, and environmental outcomes

The PICO (population, intervention, comparison, and outcome) criteria are defined in Table 1 (see Supplementary file 1, Table S1 in the Supporting Information online for a detailed list of the inclusion and exclusion criteria). Main study characteristics and nutrient, health, and environmental outcomes were extracted (see Supplementary files 1 and 3 in the Supporting Information online for more details).

**Table 1:** PICO criteria for inclusion of studies

Parameter	Theme outcome	Study selection criteria
Population		High-income countries
Intervention or exposure		Novel plant-based foods and animal-based foods
Comparison		Animal-based foods
Outcomes	Health	Dietary risk-related outcomes, mental and dental health outcomes
	Nutrient^a^	Macronutrients: saturated fat (g/100 g), total sugar (g/100 g), energy (g/100 g), and fiber (g/100 g) Micronutrients: calcium (mg/100 g), iodine (μg/100 g), iron (mg/100 g), and vitamin B_12_ (μg/100 g)
	Environment^a^	Greenhouse gas emissions (kg CO_2_ equivalent/100 g); land use (m^2^/100 g); blue-water footprint (L/100 g)

a PB drinks and milk reported in 100 ml of product.

NPBFs and their ABF counterparts were categorized into food groups on the basis of their primary ingredient (Table 2). See Supplementary file 1, sections 2.4 and 2.5, in the Supporting Information online for more details on the selection of nutrients, data analysis assumptions, and ABF baseline comparators). The following terms for each NPBF type are used in this review:

**Table 2:** Food groups for novel plant-based foods and animal-based foods and their respective reported main primary ingredient

Food group	Primary ingredient
Novel plant-based foods	
Cereals and grains	Barley, millet, oat, quinoa, rice, spelt, wheat
Coconut	Wick, meat, and flesh of coconut palm fruit
Legumes	Bean, chickpea, fava bean, lentil, lupin, pea, soy
Mycoprotein	Mycoprotein: protein made of fungus *Fusarium venenatum*
Nuts and seeds	Almond, canary grass, cashew, hazelnut, hemp, macadamia, pistachio, pumpkin seed, sesame seed, sunflower seed, tiger nut, walnut, peanuts^a^
Fruits and vegetables	Vegetables (as reported by authors), mushrooms, potatoes, and jackfruit
Unknown	Undefined plant-based product blends^b^ or unknown^c^
Animal-based foods (baseline)	
Dairy	Bovine milk (whole, reduced fat, and skimmed); goats’ milk; bovine yogurt (natural, low fat, and nonfermented); bovine cheese (all types); and sheep cheese (all types)
Meat and poultry	Beef (conventional/grass-fed ground, mincemeat, burger, meatballs); pork (sausages and primal cuts); chicken (breast, nuggets, burger, fillet) and lamb meat

a For the purposes of this review, peanuts were included in the Nuts and Seeds group because they are typically consumed as such.

b Blended or mixed products, if reported, the first ingredient was taken as the primary ingredient. For example, soy & almond PB drinks were labelled as legumes.

c If a product did not report any ingredients, they were categorised as unknown.

- PB meat products or alternatives: include different types of PB meats (eg, PB chicken, sausages, mincemeat), categories (eg, mycoprotein, legumes), and brands
- PB drink products or alternatives: include different PB drink categories (eg, legumes, nuts, seeds) and brands
- PB yogurt products or alternatives: include different PB yogurt categories (eg, legumes, coconut) and brands
- PB cheese products or alternatives: include different types of PB cheese categories (eg, coconut, nuts, seeds) and brands
- PB egg products or alternatives: include different types of PB egg categories and brands

Mention of PB products (without further specification) refers to all the listed product subcategories mentioned, except for PB eggs.

### Assessment of robustness and relevance

A modified version of the Critical Appraisal Skills Program checklist for randomized controlled trials^64^ was adapted to assess robustness and relevance of the studies in the full-text reviewing stage. The modifications involved the exclusion of the randomization, blinding, and cost-effectiveness criteria on the Critical Appraisal Skills Program checklist, and funding source was added as a criterion. Studies were assessed by 4 reviewers (G.H., R.P., S.P., and S.N.E.). Studies were assessed as follows: (1) clear description of the study design, (2) appropriate comparison group, (3) clear description of the methods, (4) rigorous and clearly described analysis, (5) funding source, and (6) precision of measure of effect. Studies with a minimum score of 1 were included, and sensitivity analysis was performed by funding source (see Supplementary file 1, section 2.2, in the Supporting Information online for more details).

### Fruit, vegetable, legume, and nut content in novel plant-based foods

In addition to the review component, a cross-sectional analysis was conducted to examine the total fruit, vegetable, legume, and nut content (percentage estimate) of each type of NPBF sold in the United Kingdom. For this, a time-stamped data set of observations from UK supermarkets generated by FoodDB in October 2021 was used. Details are described elsewhere^65^ and in Supplementary file 1, section 2.7, in the Supporting Information online. Detailed data at the global level are not available to date; hence, this part of the analysis is limited to the United Kingdom only.

### Sensitivity analysis

A common concern about studies on the health impacts and environmental sustainability of NPBFs is that they can be funded by the industry that produces them; hence, we conducted a sensitivity analysis by funding source. Furthermore, given that relative improvements in health and environmental sustainability depend on the baseline comparator used (Supplementary file 1, section 2.2, in the Supporting Information online), the sensitivity analysis based on the main primary ingredient of a given NPBF and its respective ABF comparator was also performed. The Wilcoxon test for sensitivity analysis with a significance level set at *P* ≤ 0.05 was used.

## RESULTS

### Systematic search results

**Figure 1:**
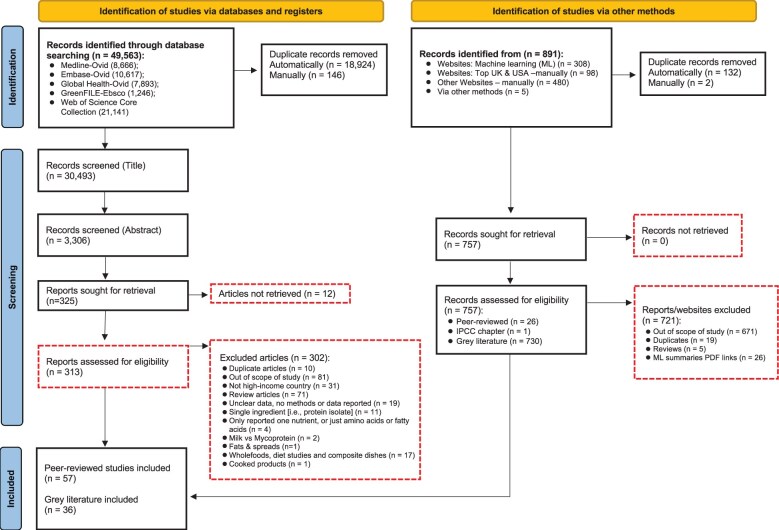
**Preferred Reporting Items for Systematic Reviews and Meta-Analyses (PRISMA) flowchart of systematic review process reporting nutrient composition, and environmental and health outcomes of novel plant-based products in high-income countries**. *Abbreviations*: IPCC, Intergovernmental Panel on Climate Change.

A total of 49 563 peer-reviewed and 891 grey literature records were identified from the initial search. After unique literature sources were screened, 57 peer-reviewed articles and 36 grey literature studies met the inclusion criteria (Figure 1). Supplementary 1, section 2, in the Supporting Information online provides further details on the screening process. The study characteristics that were extracted included basic study details (eg, authors, year, type of study, country, number of participants, follow-up period), relevant macro- and micronutrient content (eg, those related to common deficiencies, such as iron, calcium, vitamin B_12_), health and health proxy data (eg, obesity, micronutrient status, risk factors related to noncommunicable diseases), and environmental variables (eg, carbon, water, and land-use data).

### Nutrient composition of novel plant-based foods

The nutrient content of NPBFs was the most frequently studied outcome (n = 56 studies). Nutrient data were typically collected through supermarket cross-sectional surveys or manufacturers’ websites. PB meat alternatives (n = 35) and PB drink alternatives (n = 19) were most frequently reported; fewer studies researched PB cheese (n = 5) and yogurt alternatives (n = 4). No studies were found that assessed PB egg alternatives. The nutritional profile of NPBFs varied greatly by manufacturing process, including the main base ingredient (eg, soy, almond); the processing techniques, time, and temperature applied; and the type of product manufactured (ie, PB drinks, PB meats).^39^^,^^40^^,^^66^^,^^67^

#### Energy density, saturated fat, fiber, sugar, sodium, and micronutrient content of plant-based meat alternatives

**Figure 2:**
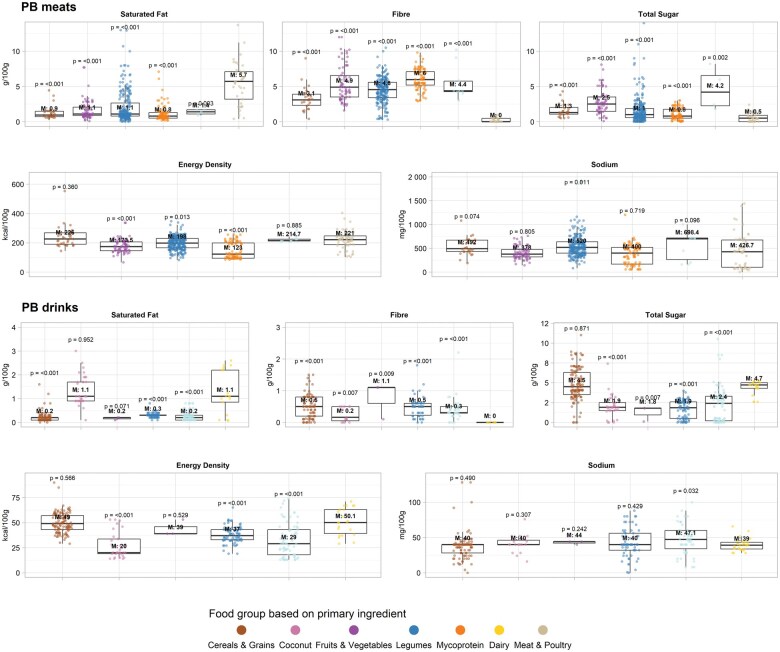
**Macronutrient, sodium, and energy content in plant-based meat and drink alternatives in their respective food group based on main primary ingredient (ie, predominant or core food item on the ingredient list) compared with meat and poultry, and dairy, respectively**. Data were limited to raw products only. *Abbreviation*: M, median of each category.

The 35 publications evaluating PB meat alternatives reported on 508 PB meat products with 66 ABF comparators. Where the median values for meat comparators were reported to be 221.0 kcal/100 g (interquartile range [IQR], 186.6–246.7), 5.7 g/100 g saturated fat (IQR, 3.2–7.1), and very low fiber (<0.1 g/100 g; IQR, 0.0–0.5), most meat-alternative groups were reported to have lower energy density, lower saturated-fat content, and more fiber (Figure 2 and Supplementary file 2: Table S1 for detailed macronutrient information disaggregated by main ingredient). Mycoprotein-based meat alternatives were reported to be the least energy dense, with a median energy value of 123.0 kcal/100 g (IQR, 94.0–198.5; with ABFs, *P* value of difference [*P*_d_] < 0.001), whereas meat alternatives based on cereals and grain had the highest energy density of all PB meats (226.0 kcal/100 g [IQR, 189.8–268.5]; *P*_d_ < 0.360), with values very similar to those of meat and poultry. Mycoprotein-based meats were also reported to be lowest in saturated fat (0.8 g/100 g [IQR, 0.5–1.3]; *P*_d_ < 0.001), whereas nut- and seed-based meats had the highest saturated fat content (1.4 g/100 g [IQR, 1.1–1.7]; *P*_d_ = 0.003) of all PB meats, which still was significantly lower than saturated fat content in meat and poultry. Finally, mycoprotein-based meat was reported to contain the highest fiber content (median, 6.0 g/100 g [IQR, 5.2–7.1]; *P*_d_ < 0.001), whereas cereal- and grain-based meats had the lowest fiber content of all PB meats (3.1 g/100 g [IQR, 2.3–3.9]; *P*_d_ < 0.001), which still was significantly higher than in meat and poultry.

Meat and poultry contained a median of 0.5 g/100 g total sugar (IQR, 0.0–0.9) and 426.7 mg/100 g sodium content (IQR, 101.0–672.8). All PB meats contained more total sugar but had similar levels of sodium in comparison with meat and poultry. Mycoprotein-based meats had the lowest total sugar content of all PB meats (median, 0.8 g/100 g [IQR: 0.5–1.8]; *P*_d_ < 0.001], and nut- and seed-based meats contained the highest total sugar amount (median, 4.2 g/100 g [IQR, 2.3–6.6]; *P*_d_ = 0.002); both showed strong evidence of being higher in total sugar content than meat and poultry. This is equivalent to 0.4 g and 3.4 g of total sugar/80.0 g serving size, or, if these sugars are considered free, 1.6% and 13.4% of the maximum recommended approximately 25.0 g average daily sugar intake.^68^ Finally, the median sodium values for all PB meat groups did not show strong evidence of a difference from meat and poultry, except for legume-based meats (median, 520.0 mg/100 g [IQR, 400.0–636.0]; *P*_d_ = 0.011). This is equivalent to 416.0 mg of sodium (or 1.0 g of salt) per 80.0 g serving size, or 20.8% of the maximum recommended 5.0 g average daily salt intake. Moreover, there were extreme outliers, with some PB meats reported to contain more than 1400.00 mg sodium (equivalent to 2.8 g salt) per 80.0 g; thus, consumption of 1 portion of this PB meat alternatives is more than half the recommended maximum daily intake of salt.^69^

Only a few studies (n = 9) evaluated micronutrient data; these reported on 250 PB meat products and 24 ABF comparators. Micronutrient content ranged vastly across all groups: whereas some products would provide substantial contributions to average daily requirements, others were much less nutritious (Table 3 and Supplementary file 2: Table S2).^69–83^ For example, the median iron content for cereal- and grain-based PB meats (5.4 mg/100 g [IQR, 4.2–5.4]) was higher than the median of meat and poultry (1.3 mg/100 g [IQR, 1.1–1.6]). On the contrary, vitamin B_12_ levels were lower for PB meat alternatives (medians ranged from 0.1 μg/100 g [IQR: 0.0–0.9] to 0.3 μg/100 g [IQR: 0.3–0.3]) as compared with 1.2 μg/100 g (IQR: 0.6–1.6) in meat and poultry. However, certain individual products had a comparable or higher vitamin B_12_ content than their ABF comparator.

**Table 3:** Summarized micronutrient values for PB meat and drinks and animal-based foods^a^

Micronutrient	PB product	Non-PB product	Global ADR (Adults aged 18 to <70 y)
	**PB drinks (combined groups)**	**Dairy milks**	
Calcium (mg/100 mL)			
No. of products	232	34	Men: 860–750 mg/d^83^ Women: 860–750 mg/d^83^
Median [IQR]	120 [120.0–120.0]	118.5 [109.7–124.0]	
min-max	0.0–203.3^70^^,^^71^^,^^72^	101.0–138.2^72^^,^^73^	
Iodine (μg/100 mL)			
No. of products	153	10	Men: 95 μg/d^83^ Women: 95 μg/d^83^
Median [IQR]	0.0 [0.0–1.4]	33.9 [21.2–38.2]	
min-max	0.0–35.0^70^^,^^71^^,^^76^	20.0–73.0^70^	
Sodium (mg/100 mL)			
No. of products	247	25	Men and women: <2000 mg/d^69^
Median [IQR]	40.0 [32.0–48.0]	39.3 [34.5–44.0]	
min-max	0.0–400.0^71^^,^^77^	31.8–65.7^73^	
	**PB meat alternative (combined groups)**	**Meat and poultry**	
Iron (mg/100 g)			
No. of products	248	24	Men: 6–19.2 mg/d^83^ Women: 6–22.4 mg/d^83^
Median [IQR]	0.7 [0.7–2.1]	1.3 [1.1–1.6]	
min-max	0.3–10.0^78^	0.6–2.1^79^^,^^80^	
Vitamin B_12_ (μg/100g)			
No. of products	227	16	Men: 2 μg/d^83^ Women: 2 μg/d^83^
Median [IQR]	0.1 [0.1–0.3]	1.2 [0.6–1.6]	
min-max	0.0–7.10^75^^,^^78^	0.2–2.2^80^^,^^81^	
Sodium (mg/100 g)			
No. of products	495	50	Men and women: <2000 mg/d^69^
Median [IQR]	480.0 [360.0–600.0]	426.75 [101.0–672.8]	
min-max	56.0–7200.0^74^^,^^78^^,^^82^	0.0–1440.0^79^	

a Values are compared with global average daily requirements (see Supplementary file 2 in the Supporting Information online for detailed information containing all disaggregated numbers by main ingredient of each novel plant-based food and animal-based foods). The table only reports micronutrients commonly found in meat and dairy. PB products also provided other micronutrients not commonly found in meat and dairy (ie, calcium in PB meats).

*Abbreviations*: ADR, average daily requirement; max, maximum; min, minimum; IQR, interquartile range; PB, plant-based.

No studies reported nutrient data from organic products. Although protein levels were not the main focus of this study, protein results are reported in Supplementary file 1: Figure S2 and Supplementary file 2: Table S1, and show that, particularly, legume- and mycoprotein-based PB meats typically match meat and poultry in protein content.

#### Energy density, saturated fat, fiber, sugar, sodium, and micronutrient content of plant-based drinks

The 19 studies evaluating PB drinks reported on 397 PB drinks (unflavored and unsweetened) and 52 dairy milk products. Where dairy milk comparators were reported to contain median values of 50.1 kcal/100 mL energy density (IQR, 39.3–63.0), 1.1 g/100 mL saturated fat (IQR, 0.9–2.2), and no fiber (0.0 g/100 mL; IQR, 0.0–0.0), most PB drink groups were reported to have lower energy density, lower saturated fat content, and more fiber (Figure 2 and Supplementary file 2: Table S1). Coconut-based drinks were reported to be the least energy dense (median energy value, 20.0 kcal/100 g [IQR: 19.0–33.7]; *P*_d_ < 0.001), whereas drinks based on cereals and grains had the highest energy density of all PB drinks (median, 59.0 kcal/100 mL [IQR: 43.0–57.0]; *P*_d_ = 0.566) but not significantly higher than dairy milks. PB drinks made of cereals and grains, fruits and vegetables, and nuts and seeds were reported to be lowest in saturated fat (median, 0.2 g/100 mL; IQRs, 0.1–0.2, 0.2–0.2, and 0.1–0.3, respectively; *P*_d_ < 0.001), whereas coconut-based drinks had the highest saturated fat content (median, 1.1 g/100 mL; IQR, 0.9–1.7; *P*_d_ = 0.952) of all PB drinks, but this was not significantly different than dairy milks. All PB drinks contained more fiber than dairy milks; however, only the drinks based on cereals and grains, legumes, and nuts and seeds were significantly higher in fiber when compared with dairy milks (for cereals and grains, and for legumes: median, 0.5 g/100 mL [IQRs, 0.2–0.8 and 0.2–0.6, respectively]; and for nuts and seeds, 0.3 g/100 mL [IQR, 0.3–0.5]; *P*_d_ < 0.001).

Dairy milks contained a median of 4.7 g/100 mL total sugar (IQR, 4.3–5.0) and 39.1 mg/100 mL sodium (IQR, 33.6–43.3). Most PB drinks contained less total sugar than did dairy milks, but they had similar levels of sodium. However, the total sugar content was only significantly lower for coconut (median, 1.9 g/100 mL; IQR, 1.5–2.5), legumes (median, 1.9 g/100 mL; IQR, 0.5–2.6), and nut- and seed-based drinks (median, 2.4 g/100 mL; IQR, 0.2–3.3) when compared with dairy milks (*P*_d_ < 0.001). This is equivalent to 3.8 g and 4.8 g of total sugar/200.0 mL serving size, or, if these sugars are considered free, 15.2% and 19.2% of the maximum recommended 25.0 g average daily sugar intake.^68^ The only PB drink group that was statistically different in sodium content compared with dairy milks was the group based on nuts and seeds (median, 47.2 mg/100 mL [IQR, 34.0–60.0]; *P*_d_ = 0.032). This is equivalent to 94.4 mg of sodium (0.2 g of salt) per 200.0 mL serving size, or 4.0% of the maximum recommended 5.0 g average daily salt intake.^69^ However, there were also some extreme outliers, some of which reported containing more than 3 times this amount of sodium per 200.0 mL, the equivalent of approximately 12.0% of the daily World Health Organization recommendation.^69^

A few studies (n = 16) evaluated micronutrient data of PB drinks, reporting on 249 PB alternative products and 37 ABF comparators. Iodine was only reported in PB drinks, not in other types of PB products. Like PB meat alternatives, micronutrient content ranged vastly across all groups: some products contributed to the average daily requirement, whereas others were much less nutritious (Table 3 and Supplementary file 2: Table S2). For example, the median calcium content for all PB drink categories was 120.0 mg/100 mL (IQRs as follows: cereals and grains, 120.0–120.0; coconut, 120.0–120.0; fruits and vegetables, 120.0–120.0; legumes, 120.0–120.0; nuts and seeds, 114.5–120.0) as compared with 116.7 mg/100 mL (IQR, 109.3–124.0) for dairy milks. However, none of the PB products (median, 0.0 μg/100 mL; IQR, 0.0–1.4) matched the iodine content of dairy milks (median, 24.9 μg/100 mL; IQR, 20.0–36.5).

Only 4 studies (evaluating 29 PB drinks and 11 dairy milk products) reported nutrient data from organic PB products. All evaluated different nutrients, hence no further pooling of results was possible for organic products as a subgroup. Protein results are reported in Supplementary file 1: Figure S2 and Supplementary file 2: Table S1 in the Supporting Information online, which show that, particularly, legume-based PB drinks typically match dairy milk in protein content.

#### Energy density, saturated fat, fiber, sugar, sodium and micronutrient content of plant-based yogurt alternatives

The 4 studies on PB yogurt alternatives evaluated 191 PB yogurt products with 90 dairy-based comparator products (unflavored and unsweetened). The overall nutritional composition of PB yogurts appears to show some variation by main primary ingredient (see Supplementary file 2 in the Supporting Information online); however, formal disaggregated assessment of PB yogurts by primary ingredient was not possible, because that information was often not reported by authors. At an aggregate level, PB yogurts typically contained less saturated fat and sodium but had a higher energy density and higher total sugar and fiber content.

Only 2 studies evaluated micronutrient data of PB yogurts (excluding sodium) and, therefore, no further pooling of results was possible. No studies reported nutrient data from organic PB yogurts. Protein results are reported in Supplementary file 2: Table S1 in the Supporting Information online. Only the sample of a legume-based PB yogurts came close to matching dairy yogurt in protein content.

#### Energy density, saturated fat, fiber, sugar, sodium and micronutrient content of plant-based cheese alternatives

The 5 studies evaluating PB cheese alternatives reported on 163 PB cheese products with 143 dairy-based comparator products. PB cheese alternatives were the least nutritionally diverse foods. Where the primary ingredient of PB cheeses was known, this was mostly coconut oil (Supplementary file 2 in the Supporting Information online); however, like PB yogurts, the main ingredient was often not reported by authors.

The cheese comparators were reported to contain median values of 284.0 kcal/100 g energy density (IQR, 108.0–330.1), 14.0 g/100 g saturated fat (IQR, 11.0–17.3), and no fiber (0.0 g/100 g; IQR, 0.0–0.0). Most PB cheese subgroups were reported to have higher energy densities and higher saturated fat and fiber content. PB cheese based on nuts and seeds had the highest energy density (328.0 kcal/100 g [IQR, 306.0–328.0]; *P*_d_ = 0.334]), whereas coconut oil-based cheese had the highest saturated fat content (21.0 g/100 g [IQR, 19.7–22.0]; *P*_d_ < 0.001]), a significant difference, with 50.0% more than dairy cheese. Unlike PB drinks, PB meat, and PB yogurt alternatives, not all PB cheese contained fiber. Nut- and seed-based cheese had the highest fiber content (median, 2.5 g/100 g [IQR, 2.4–2.7]; *P*_d_ < 0.001). Although the median fiber content of PB cheese made from coconut oil was 0.0 g/100 g (IQR, 0.0–1.7; *P*_d_ = 0.011), some products did contain up to 5.9 g/100 g and, therefore, strong evidence was found that both PB cheese based on nuts and seeds and on coconut oil had significantly higher fiber content than did dairy cheese.

Most PB cheese contained less sugar and sodium than did dairy cheese, which had a median of 2.0 g/100 g (IQR, 0.5–5.0) and 720.0 mg/100 g (IQR, 560.0–1000.0), respectively, across the identified studies. In general, PB cheese alternatives had either no or minimal total sugar content. Finally, coconut oil–based cheese had the highest sodium content across all PB cheese (median, 714.0 mg/100 g [IQR, 600.0–880.0]; *P*_d_ = 0.897), but this was similar to dairy cheese. PB cheese made of nuts and seeds had the lowest median sodium content (240.0 mg/100 g [IQR, 200.0–240.0]; *P*_d_ = 0.001), which would equal 48.0 mg of sodium (0.1 g of salt) per 20.0 g serving size, or 2.0% of the recommended maximum daily salt intake^69^; hence, this type of PB cheese had a large reduction in sodium compared with dairy cheese.

The micronutrient content of PB cheese was evaluated by only 2 studies. Only 1 product made of nuts and seeds was fortified with calcium, whereas coconut-based PB cheese was typically fortified with vitamin B_12_ (median, 2.5 μg/100 g; IQR, 2.5–2.5). For dairy cheeses, these medians were 815.0 mg/100 g (IQR, 463.0–930.0) for calcium and 2.5 μg/100 g (IQR, 1.8–2.5) for vitamin B_12_.

No studies reported nutrient data from organic products. Protein results are reported in Supplementary file 2: Table S1 in the Supporting Information online. Nut- and seed-based cheese typically had the highest protein content, though it did not match the protein content of dairy cheese.

### Health impacts and risk factors of novel plant-based foods

Eleven peer-reviewed studies were included in this review, 9 of which evaluated PB meat alternatives and 3 evaluated PB drinks (Table 4)^84–94^ (see Supplementary file 1, section 3.3, in the Supporting Information online for further details on the health outcomes). No health studies were found that evaluated consumption of PB cheese, yogurt, or egg alternatives; links between NPBFs and mental health outcomes; nor any grey literature evaluating any health outcomes.

**Table 4:** Summary of the evidence on the health impacts and risks of novel plant-based foods

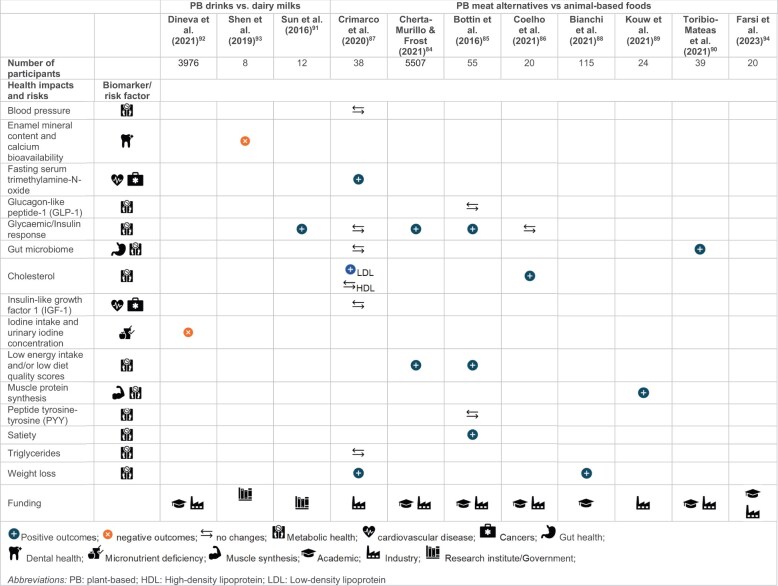

#### Health impacts and risk factors of plant-based meat alternatives

Studies of PB meats (n = 9) showed positive health outcomes when individuals switched from consuming ABFs. Three studies on mycoprotein consumption by both healthy and overweight adults found a positive association with lower glycemic markers,^84^ reduced energy intake,^84^^,^^85^ and insulin release.^85^ Moreover, mycoprotein consumption was hypothesized to have a beneficial impact on the plasma lipidome.^86^

Four studies with healthy adults evaluated PB meat alternatives consumption (other than mycoprotein). When considering the same caloric intake, consumption of PB meats was associated with a lower risk of cardiovascular disease than was consumption of ABFs, mostly by reducing fasting serum levels of trimethylamine-*N*-oxide, and low-density lipoprotein cholesterol concentrations, compared with ABF consumption.^87^ Furthermore, consumption of PB meats was associated with a reduction in body weight as compared with meat consumers.^87^^,^^88^ Lysine-enriched PB meat as a substitute for ABFs was reported to increase muscle protein synthesis rates, which is a biological process of building new protein cells via amino acids.^89^ Last, the replacement of 4 meat-containing meals per week with PB meat alternatives elicited positive changes in the gut microbiome, with changes in the presence of butyrate-producing pathways and increased taxa.^90^

#### Health impacts and risk factors of plant-based drinks

Studies assessing PB drinks (n = 3) only focused on almond and soy drinks. The main focus and health outcomes of these studies varied. Sun et al^91^ researched the reduction in glycemic response in young adults consuming soy drink or bovine milk together with white bread. These authors found that both products had a similar glycemic response through different biological pathways. Dineva et al^92^ assessed micronutrient content in PB drinks and found significantly lower iodine intake and urinary iodine concentration in people consuming only PB drinks,^93^ highlighting the need for appropriate fortification as more people transition to eat more NPBFs. Finally, Shen et al^93^ evaluated the impact of PB drinks on dental health and found that a soy drink with added sugar caused enamel demineralization, compared with dairy milk, which promoted remineralization.

### Environmental impacts of novel plant-based foods

**Figure 3:**
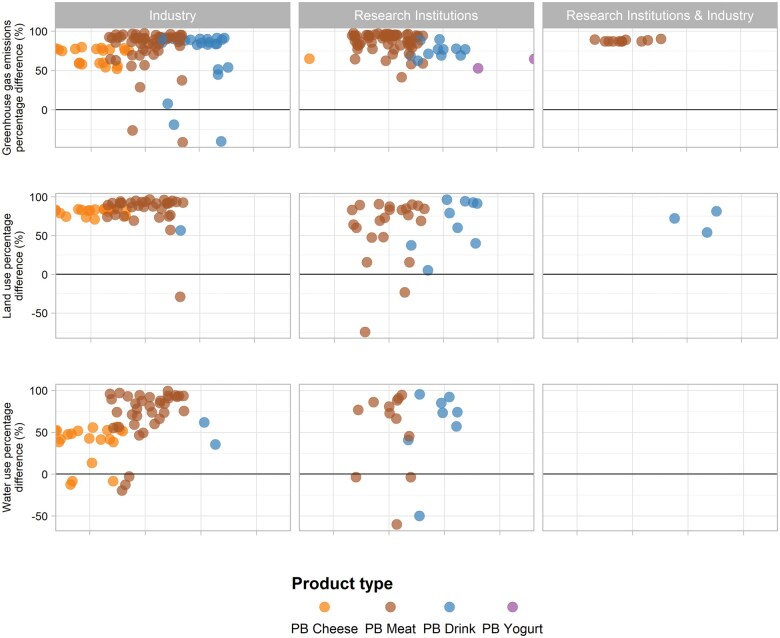
**Reduction of environmental impacts by respective funding source**. Calculated as a percentage difference between each novel plant-based (PB) product (by product type and food group based on main primary ingredient [ie, predominant or core food item on the ingredient list]) in comparison with their respective reported baseline (eg, dairy milk and cheese, meat and poultry). See Supplementary file 3 in the Supporting Information online for detailed information on the baseline used for each reference. Data were limited to raw products only. Studies reporting data on cooked PB products also found reductions in environmental impacts.

A total of 53 studies evaluated at least 1 environmental outcome, using the life cycle assessment method, evaluating 209 PB products and 91 ABFs as comparators. Most studies used life cycle assessment inventories, and some relied on data providers (n = 32) to calculate environmental footprints. System boundaries varied across studies, with the majority evaluating category impacts from cradle-to-retail (see Supplementary file 3 in the Supporting Information online). Studies mainly assessed the effect of substituting ABFs with NPBFs on greenhouse gas emissions (GHGE) (n = 50), followed by blue-water footprint (WF) (n = 39) and land use (LU) (n = 17) (Figure 3 and Supplementary file 1: Table S11 in the Supporting Information online). Although methods, assumptions, and inventory data varied from 1 study to another, most studies consistently reported percentage reductions in GHGE and LU for the production of NPBFs as compared with ABFs. Wider differences were observed in blue WF.

#### Environmental footprints of plant-based meat alternatives replacing meat and poultry

The 34 publications evaluating PB meat alternatives reported on 135 PB meat products with 53 ABF comparators. The percentage difference showed reductions of more than 70% in GHGE, LU, and WF for most products when shifting from ABFs to PB meat alternatives. GHGE reductions across PB meat groups, based on primary ingredients, were similar, with the largest reduction in GHGE seen for nut- and seed-based meats, with a median value of –94.2% (IQR, –94.4 to –93.4), whereas PB meats based on legumes had the smallest reduction (–86.1%; IQR, –88.6 to –77.5). Only 2 of 134 PB products had higher levels of GHGE than their ABF comparator. For LU, mycoprotein (median, 89.0%; IQR, –92.3 to –76.5) and nut- and seed-based meats (median, 89.5%; IQR, –90.0 to –89.0) had the largest reduction. Alternatively, legume-based meats had the smallest LU reductions (median, –71.2%; IQR, –84.7 to –47.6). Only 3 of 55 products had higher LU than their ABF comparator. Finally, the largest reduction of WF was observed in PB meats made of cereals and grains (median, –92.6%; IQR, –94.1 to –92.0), and the smallest was observed with products made of mycoprotein (median, –73.7%; IQR, –84.4 to –55.2). Nine of 51 products had a higher WF than their respective ABF counterparts. Specifically, when certain individual legume- and mycoprotein-based meats were compared with chicken, PB meat alternatives reported requiring between 2.7% and 339.0% more water, with the largest difference observed in a Swedish chicken comparator to mycoprotein-based meats. This variation was attributed to differences between feed types, rearing systems, and farm efficiency across countries.^74^ Comparisons were also made between the upper limit footprint of mycoprotein-based items and the average or lower limit footprint of the ABF. Moreover, there were extreme outliers, with some PB meats reporting a water percentage difference of 8006.9%. The authors attributed this to soybeans’ substantial water demand during processing and lower yield per soybean.^74^

#### Environmental footprints of plant-based drinks alternatives replacing dairy milk

The 21 publications evaluating PB drinks reported on 51 PB drink products with 13 ABF comparators. PB drinks also were associated with reductions in GHGE and LU when shifting from dairy milk to PB drinks. Fruit- and vegetable-based drinks had the largest reduction of GHGE (median, –90.2%; IQR, –90.8 to –90.2]), whereas PB drinks based on cereals and grains had the smallest reduction (median, –76.9%; IQR, –88.8 to –56.0). Only 2 products of 36 had an increase of GHGE when comparing soy- (40.0%) and almond-based (18.9%) drinks with dairy milk (equivalent to 0.3, 0.4, and 0.3 kg CO_2_ eq/100 g, respectively).^95^ Wider differences were observed on the LU percentage difference; however, reductions were found for all products (n = 13 PB drinks).

Cereal- and grain-based drinks had the largest reduction (median, –86.4%; IQR, –92.7 to –76.0), whereas legume-based drinks had the smallest LU reductions (median, –56.6%; IQR, –75.5 to –38.8). The magnitude of change in the percentage difference for WF varied considerably, although, these data were less frequently reported by authors (n = 11 PB drinks). Cereal- and grain-based drinks had the largest reduction (median, –85.0%; IQR, –88.7 to –71.0), whereas legume-based drinks had the smallest WF reductions (median, –67.6%; IQR, –73.9 to –42.2). Nut- and seed-based drinks presented contradictory evidence. For example, Grant and Hicks^95^ observed that almond drinks (9241.9%) required considerably more water than soy (–35.6%) and dairy milks (equivalent to 109.3, 0.8, and 1.2 L/100 g, respectively); whereas Ritchie^96^ found that an almond drink required half the amount of water (–40.87%) than dairy milk (equivalent to 37.2 and 62.8 L/100 g, respectively). Data were limited to these 2 products; hence, no further pooling of results was possible.

#### Environmental footprints of plant-based yogurt alternatives replacing dairy yogurt

The 2 publications evaluating 2 PB yogurt alternatives compared to 2 dairy yogurts. They reported GHGE reductions ranging between –64.7% and –52.9%. Analysis of LU and WF was not possible due to lack of a baseline, differences in methods, and system boundaries.

#### Environmental footprints of plant-based cheese alternatives replacing dairy cheese

The 2 publications evaluating PB cheese alternatives reported on 21 PB cheese products with 23 ABF comparators. Data on the environmental impacts were particularly from coconut oil–based cheese alternatives (n = 20). All coconut oil–based cheese alternatives had a large reduction in amounts of GHGE and LU (GHGE: median, –75.4% [IQR, –77.4 to –59.3]; LU: median, –83.1% [IQR, –83.8 to –80.6]). A smaller reduction was observed in WF (median, –45.1%; IQR, –52.0 to 38.5), with a higher WF being reported than for the ABF comparator for only 3 products.

### Health effects and environmental impacts of novel plant-based foods

**Figure 4:**
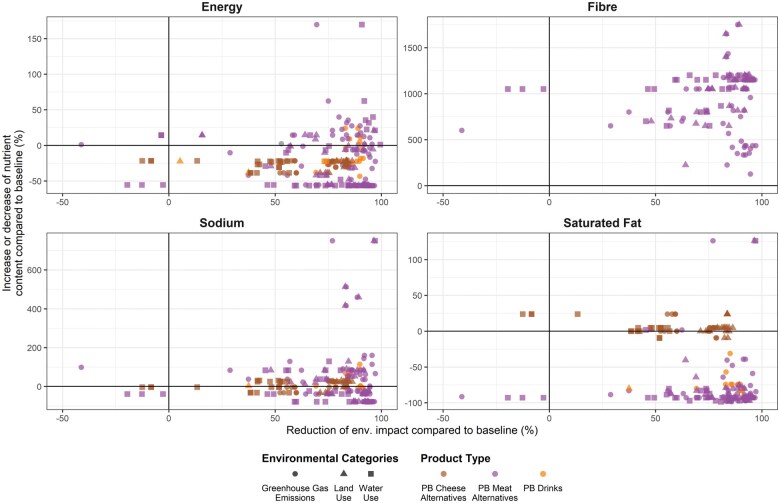
**Reduction of environmental outcomes and their associated nutrient outcomes of novel plant-based foods (NPBFs) compared with baseline** (eg, dairy milk and cheese, meat and poultry), **expressed in percentage difference**. The *y*-axis shows the increase or decrease of the nutrient content (energy, fiber, sodium, and saturated fat) in comparison with baseline; and the *x*-axis shows the reduction (or increase) of the environmental categories. Three environmental categories are reported: greenhouse gas emissions (circles), land use (triangles), and blue-water use (squares). Three NPBFs are reported: plant-based (PB) cheese alternatives (brown), PB meat alternatives (purple), and PB drinks (orange). PB yogurts were not included due to the limited amount of data. See Supplementary file 2 in the Supporting Information online for detailed information on the baseline used for each reference. Data were limited to raw products only.

Studies that simultaneously assessed both health and environmental outcomes and/or nutrient profiles of NPBFs were pooled (Figure 4). Only 1 study reported environmental outcomes together with diet-related health effects of PB meat alternatives, and this study found that free access to NPBFs was associated with greater weight loss and reduced dietary carbon and LU, as compared with a control arm.^88^ From 93 references, 20 studies assessed the environmental outcome and nutrient content of NPBFs; only 6 studies evaluated the health effects and nutrient content of NPBFs (see Supplementary file 1: Table S9 in the Supporting Information online).

When compared with ABF counterparts, data suggest NPBFs are overwhelmingly associated with smaller environmental footprints. Data on nutritional profiles of NPBF were mixed: nutritional profiles for some NPBF groups were better aligned with healthy diets, but not for others. Clear co-benefits were observed for fiber intake from NPBFs. However, for the other nutrients, the picture was much more mixed due to the variability in content arising from differences in the main primary ingredients and the type of NPBFs.

### Fruit, vegetable, legume, and nut content of novel plant-based foods

**Figure 5:**
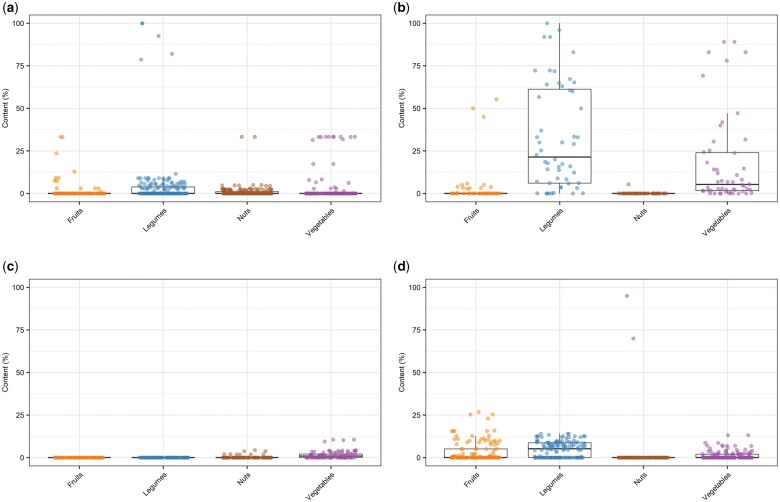
**Estimated fruit, vegetable, legume, and nut content (%) in each novel plant-based foods product from time-stamped data from UK supermarkets**. Panels show (a) plant-based (PB) drink alternatives; (b) PB meat alternatives; (c) PB cheese alternatives; and (d) PB yogurt alternatives.

The percentage of fruit, vegetable, legume, and nut content in each NPBF in the United Kingdom was estimated as a case study (Figure 5). Most NPBFs had at least 1 fruit, vegetable, legume, or nut, ranging from 0.0% to 100.0% of their weight. Overall, median content was low, with a few exceptions. PB meat alternatives had the highest content of vegetables and legumes, and PB cheese alternatives had the lowest content (Supplementary file 1: Figure S5 and Supplementary file 2: Table S5 in the Supporting Information online).

### Assessment of robustness and relevance of the included studies

For results on the assessment of robustness and relevance of the included studies see Supplementary file 1: Table S12 in the Supporting Information online in section 3.6.

#### Sensitivity analysis of funding sources of nutrient composition studies

Almost half of the nutrition studies included (n = 26; 46.4%) were funded by academic funders; 44.6% (n = 25) were fully funded or partially funded by industry; and 10.0% (n = 5) did not state their funding source. NPBF manufacturers were the support for the majority of industry-funded studies (n = 21; 37.5%), followed by the livestock industry (n = 3; 5.4%), and both (n = 1; 1.8%). The sensitivity analysis of the percentage difference for all the nutrients associated with the burden of disease, except total sugar, revealed that studies funded by industry were more likely to find differences than those funded by academia, with the former typically reporting more positive results on lower energy and saturated fat (Table 5 and see Supplementary file 1: Table S13 in the Supporting Information online for sensitivity analysis on studies partially funded by the industry). However, the direction across all studies was the same: reductions in energy and saturated fat content, and increases in fiber, total sugar, and sodium content.

**Table 5:** Sensitivity analysis, based on funding source, of the percentage difference between novel plant-based foods vs animal-based foods in nutrient content and environmental impacts^a^

Parameter (%)	Percentage difference in health and environmental outcomes of NPBF vs ABF		Academic funder vs industry funded
	Academic funder (n = 35)	Industry funded (n = 41)	Wilcoxon test
	Median (IQR)	Median (IQR)	*P* value
Energy^b^	–0.25	–14.10	
	(–18.81 to 14.29)	(–30.90 to 1.71)	<0.001
Fiber^c^	1976.92	733.33	<0.001
	(715.27 to 3669.23)	(495.83 to 966.67)	
Saturated fat^b^	–69.01	–84.46	<0.001
	(–83.33 to 19.47)	(–89.40 to –60.43)	
Total sugar	7.69	18.31	0.060
	(–80.77 to 107.04)	(–50.70 to131.69)	
Sodium^c^	4.05	61.92	<0.001
	(–26.61 to 53.14)	(19.91 to 98.58)	
Greenhouse gas emissions^c^	–89.61	–84.36	
	(–94.25 to –77.62)	(–91.28 to –71.59)	0.001
Land use^b^	–76.35	–84.24	0.007
	(–88.01 to –47.68)	(–91.88 to –77.26)	
Water use	–73.66	–56.43	0.447
	(–86.63 to –44.31)	(–84.83 to –41.79)	

a The funding source of 6 articles were unknown, so they were excluded from this analysis. The superscript b and c indicate the direction and dimension of the association.

b Industry-funded studies show a more positive impact on health and environmental outcomes of their PB products (vs animal sourced foods) as compared with academically funded studies.

c Industry-funded studies show a less positive impact on health and environmental outcomes of their PB products (vs animal-sourced foods) as compared with academically funded studies.

*Abbreviations*: ABF, animal-based food; IQR, interquartile range; NPBF, novel plant-based food.

#### Sensitivity analysis of funding sources of health studies

Only 2 health studies were funded by academia; the rest of the studies were either partially or wholly funded by industry (n = 9). Most industry-funded studies were from NPBF manufacturers (n = 8); 1 study was partially funded by Dairy Australia.

#### Sensitivity analysis of funding sources of environmental studies

Compared with nutritional studies, a greater percentage of environmental studies were by industry researchers, particularly from NPBF manufacturers (67.9%). Approximately 71.7% of studies (n = 38) were fully funded or partially funded by industry; 26.4% (n = 14) were supported by academic funders; and 1.9% (n = 1) did not state their funding source. Of the industry-funded studies, only 2 (3.8%) were funded by the livestock industry. The sensitivity analysis revealed that the percentage differences were significantly larger between academic and industry funders in terms of GHGE and LU. Studies funded by industry typically reported more positive results on LU than did studies funded by academic funders, and the opposite was observed for GHGE. Like nutrient studies, the direction (decreases in GHGE, LU, and WF) was the same regardless of the funding source (Table 5, and see Supplementary file 1: Table S13 in the Supporting Information online for the sensitivity analysis of studies partially funded by the industry).

## DISCUSSION

### Research findings

We reviewed evidence from high-income countries that was published in peer-reviewed and grey literature within the past 7 years on nutrient content, and environmental and health outcomes of consuming NPBFs. Most NPBFs typically have much lower environmental impacts compared with ABFs, particularly with respect to GHGE and, to a lesser extent, to LU and WF. The nutrient content of NPBFs is highly variable in comparison to the nutrient profiles of ABFs. Although several individual NPBFs had positive health and environmental outcomes, co-benefits identified were not universal across all NPBFs and several trade-offs were identified. The main primary ingredient, type of product, processing techniques, and brand were all important determinants of health, and nutritional and environmental outcomes, findings that show the need for further subcategorization of NPBFs to better educate consumers and enable them to take informed decisions regarding the healthiness and sustainability of their diets and (potential) dietary changes.

### Research in context

If carefully selected, certain NPBFs (particularly certain PB drinks and meat alternatives) could be an effective part of interventions to achieve net-zero and health targets in high-income countries. By applying a combination of strategies, enhanced uptake of these foods could improve the nutritional quality of diets, improve health, and contribute to tackling climate change impacts.

At the macronutrient level, NPBFs are generally the healthier option, given their higher fiber content and typically lower saturated fat and calorie contents, which could be advantageous in high-income (often obesogenic) settings. Certain types of NPBFs, particularly mycoprotein and legume-based meats, often also contain a substantial amount of fruit, vegetables, legumes, and/or nuts, which are food groups that are typically underconsumed in high-income settings. Composition of legume and fruit and vegetable-based drinks, were also typically consistent with healthier diets in high-income food secure settings, including low energy density, low total sugar, high fiber and low saturated fat content. Caution is recommended in the selection of these products if they were to be part of dietary recommendations, or standard institutional procurement for example, as certain NPBFs can also have higher levels of total sugar, sodium, and saturated fats in comparison to their respective ABF. This is particularly true for certain cereal and grain-based drinks, and coconut-based cheese and yogurts. Although the specific type of oil used in each NPBF product was not analyzed, coconut oil, which is high in saturated fatty acids, is often the ingredient that increases saturated fat levels in NPBFs to levels similar to its ABF counterparts.^51^^,^^75^ Indeed, coconut oil-based cheese had approximately 50% more saturated fat than dairy cheese, and typically contained the least amount of fruit, vegetables, legumes or nuts, with the majority being absent.

In line with other evidence,^39^^,^^97^^,^^98^ fortified NPBFs, in some cases, can be nutritionally comparable to their respective ABFs. Some individual NPBFs contained even higher concentrations of iron, vitamin B_12_, and calcium, whereas others did not. However, micronutrient assessment was difficult because not all included studies reported micronutrients. This could be because either NPBFs were unfortified or the information simply was not reported. Especially when nutrient information is gathered from supermarket websites for individual studies, micronutrient data are generally not reported.

The highly varying nutrient content across and within all PB products and categories may cause consumer confusion when individuals are looking for healthy and environmentally friendly alternatives to ABFs. Clearer front-of-package labelling of certain nutrients and information campaigns could reduce such confusion and better enable the consumer to make informed decisions about food purchases.^99^ Potential development of rules and regulations on the food standards of NPBFs could also be a step forward in having a larger range of “healthy” NPBFs, because such regulations could potentially encourage reformulation of NPBFs, including the reduction of sodium, total sugar, and saturated fat content, and increased micronutrients. From a technological perspective, this is certainly possible. For example, new biotechnological techniques have been developed that enable companies to reduce sugar content and improve palatability, nutrient profile, and digestibility of PB drinks.^67^^,^^100–103^ Some processing techniques can also decrease levels of anti-nutrients and polyphenols, which commonly are associated with low mineral and vitamin bioavailability,^35^^,^^98^^,^^101^^,^^104–107^ and increase protein yield.^101^ Given that specific raw materials, isolated proteins, processing levels, and fortification methods, often used in NPBFs, as compared with ABF nutrient profiles, are still debated in the scientific community, further research on the nutrient content and health risks related to bioavailability, bioaccessibility, and byproduct formation during industrial processes will reveal whether there are differences in terms of health impacts of “natural” vs more “isolated” nutrients.^30^^,^^108^^,^^109^ More research into the metabolic profiles of NPBFs is imperative, particularly in light of a recent study identifying differences in the abundance of profiled metabolites between beef and PB burgers, despite their labelled nutritional similarities.^110^ Instead of continuing the debate between the superiority of ABFs vs NPBFs, or vice versa, acknowledging and embracing their complementary differences can contribute to a less polarized dietary transition. This is especially relevant because emerging evidence has suggested that people who consume NPBFs also tend to purchase ABFs.^111^

From the limited evidence on health, the inclusion of NPBFs into diets appears to typically have beneficial health effects, particularly the consumption of PB meat alternatives. The positive health effects mostly relate to better weight management and associated reduced risk of noncommunicable diseases in high-income (and often obesogenic) countries. This is aligned with a recently published meta-analysis that found positive outcomes on total cholesterol, low-density lipoprotein cholesterol and triglycerides when consuming PB meat alternatives as replacements for meat.^51^ Furthermore, a few older studies also found positive health outcomes when assessing consumption of mycoprotein-based foods (eg, drinks, cookies, milkshakes, crisps)^112–115^ and soy protein with isoflavones,^50^ compared with consumption of dairy milk and/or meat products.

Previous evidence revealed that NPBFs are often regarded as healthier alternatives to ABFs^116^; hence, it could be hypothesized that people may consume NPBFs in larger quantities than they would otherwise have done when eating ABFs. This may have negative health implications, especially if consumed regularly. Establishing a clear division in PB foods classifications, including ultraprocessed and less processed PB alternative foods, could enable better assessment of short- and long-term health impacts of NPBFs if they were to be consumed at an even larger scale.^116^

Ultraprocessed foods have been associated with many diet-related diseases because these foods are generally energy dense and hyperpalatable.^117^^,^^118^ Almost all NPBFs fall, technically, within this category; however, in this review, we found that the nutritional composition of some NPBFs aligns well with healthy dietary recommendations, such as having a high fiber content, low energy density, and low saturated fat content. Additionally, 1 of the included studies^90^ also found positive associations with the gut microbiome when substituting meat in certain meals with PB meat alternatives. To get a better overview of the overall effect of NPBFs on health, more information and detailed analyses are needed regarding level of processing and gastrointestinal fate.

Consistent evidence was found regarding environmental outcomes, similar to previous research.^52^^,^^53^^,^^108^^,^^119–121^ Most NPBFs had smaller environmental footprints than their ABF counterparts, with median reductions reported of up to 94.3%, 89.5%, and 92.6% for GHGE, LU, and WF, respectively. Nevertheless, some PB products had greater environmental impacts than their ABF counterparts, with some extreme outliers particularly in terms of WF. Although evidence was rather consistent, and the direction of effect appears to be clear, care should be taken not to overinterpret the exact numerical results: environmental impact calculations are notoriously context dependent and sensitive to methodological and data choices. This makes it impossible to come up with a summary figure that is representative for all products, produced in all countries; generally, however, there is a broad body of evidence demonstrating a reduction in GHGE, LU, and WF for a wide range of PB products in a wide variety of contexts compared with their ABF equivalents.

To improve the strategic use of NPBFs to achieve more sustainable food systems, life cycle assessments of these products should incorporate the full range of environmental impact categories, as well as sociocultural, economic, and health impacts with harmonized methods and assumptions across studies.

This study revealed an evidence gap for health impacts of NPBFs, including mental and dental health, and other risks associated with micronutrient deficiencies. There is also a lack of health studies on PB yogurts, PB cheese, and PB egg alternatives. Research on the health effects of PB drinks has been conducted with only certain products, “generally soy and almond drinks,” but there is a gap in knowledge about other PB drinks, such as those made from oat, potato, and hazelnut, among others. Furthermore, some concerns have been raised about the carbohydrate content in some PB drinks. A study by Jeske et al^122^ revealed that the presence of β-glucan in many oat-based drinks causes a moderate glycemic index, despite the high carbohydrate content. In fact, Dhankhar^104^ associated the consumption of oat drinks with high β-glucan levels with a reduction in cholesterol levels in study participants. However, this evidence needs to be updated to reflect the potential benefits of different types of PB drinks and current market brands. Although dairy products contain naturally occurring sugars from lactose, it is difficult to determine the breakdown of “natural” vs added sugars in NPBFs from the available literature. More research is also required on dental health to assess the potential risks of increased dental cavities due to lower calcium bioavailability, and the effects of free sugar content, pH levels, and buffering capacity in NPBFs.

Additional research is needed to provide more nutrient environmental and health evidence for PB yogurts, cheese, and egg alternatives. Last, although this review assessment focused on 3 environmental outcomes, evidence on other environmental impacts, including biodiversity loss and socioeconomic implications, is scarce. Across the 3 themes assessed in this review, better standardization and clear reporting of results in NPBF studies in the future would facilitate updates of this review.

### Relevance for policy and practice

Minimally processed PB foods are still considered the gold standard for healthier and more sustainable diets. However, shifts from ABFs to PB whole foods remain problematic because, despite all the scientific knowledge about healthy eating, dietary change toward minimally processed PB foods has not been achieved. This review revealed that NPBFs can be healthier and more environmentally friendly alternatives to ABF consumption, if carefully selected. Although behavioral aspects are embedded in this transition, NPBFs could offer a convenient, novel, and potentially more realistic option to facilitate dietary transitions at large scale, diversifying diets, and increasing consumption of fruits, vegetables, legumes, and nuts without the need for significant individual dietary habits.

For potential promotion of the inclusion of NPBFs as part of public procurement or embedding them into food-based dietary guidelines, some of the consideration regarding varying healthiness of specific types of NPBFs and the need for further subclassifications needs to be carefully addressed. Furthermore, affordability is a concern because NPBFs often are more expensive than their ABF counterparts. Although comprehensively synthesizing price data was outside of the scope of this study, in the United Kingdom, the Food Foundation found that PB drinks are, on average, 50.0% more expensive than dairy milk.^71^

Active promotion of NPBFs would require more detailed analysis of consumer behavior: current consumption of NPBFs is generally higher among younger generations, women, White populations, and those with higher education and incomes.^28^ Better understanding of main drivers and barriers of consumption of NPBFs would allow targeted promotion to widen this consumer group.^71^ NPBFs could play an additional role in reducing the prevalence of micronutrient deficiencies, especially given their reformulation and fortification potential. For example, in Finland, a mass fortification strategy of vitamin D across dairy and nondairy products has shown positive health outcomes over the past decade.^123^ Finally, formalization, standardization, and accountability of environmental labelling could help consumers making informed decisions and avoid misinformation.

### Strengths and limitations

To our knowledge, this is the first systematic review assessing the published peer-reviewed and grey literature evidence from studies that evaluated nutrient, and health and environmental impacts or benefits of NPBFs. A strict and comprehensive search string was developed to assess the full breadth of studies and reports, and machine-learning models were used to filter the large number of studies and systematically present all the available evidence on various NPBFs.

This study only covered the past 7 years to assess the current evidence, and an exhaustive cross-check of references was not performed, which possibly introduces reporting bias for missed relevant studies from previous years. However, it was assumed that only a small amount of additional findings had been missed, given the recent emergence of the variety and types of these novel products. Second, only 3 environmental impact categories were examined: carbon footprint, LU, and blue-water consumption. However, the heterogeneity of study designs, from system boundaries to geographical location, agricultural inputs, and methods used to calculate environmental footprints, made the review process too time consuming to expand on other environmental impacts in this particular study. Reliable reporting of environmental impacts of novel ingredients used in NPBFs, including added minerals and vitamins for fortification purposes, are generally missing in many studies. All the data reported by authors were collected and each study was compared individually against its own baseline (ie, the ABF comparator provided by author). Given the large spectrum of methods to determine environmental footprints, this could have introduced some bias; however, the alternative (using a standardized comparator) would equally have its limitations (eg, this would not be representative for all farming systems and products). Third, products and nutrients were assessed individually. Although the nutrient content gives some guidance on probable health risks, in reality, people consume diets in which individual compounds interact, influencing unknown biological pathways. Fourth, several studies that did not specifically report on the proportion and type of NPBF in (self-)reported PB diets had to be excluded. For those studies, it was impossible, therefore, to assess the effect on health and environment of NBPFs alone vs all PB foods together (ie, whole foods, NPBFs, other PB foods such as tofu and tempeh) and complicated any efforts to calculate dietary shifts. Finally, most studies did not report the precision of measures of effect (n = 68), making it difficult to pool and synthesize results across the 3 themes assessed in this review.

### Conclusion

Food systems and diets need to change to meet environmental and health targets. This comprehensive systematic review presents a holistic approach to summarize the evidence on the nutrient, health, and environmental impacts of NPBF consumption. Although PB whole foods remain the preferred option on health grounds, some NPBFs have potential for being a useful steppingstone in the process of food system and dietary transformation, functioning as a healthy and environmentally friendly alternative to ABFs, if carefully selected. Reformulation and fortification could further enhance NPBFs as a viable and effective food group that could accelerate the dietary transition toward sustainable and healthy diets. However, given the great variability in nutritional composition of individual NPBFs, widespread promotion of such products should be introduced and addressed with caution. Given that NPBFs are already important in the food system and consumption is expected to continue to increase, a few steps are urgently required to guide consumers and enable them to make informed decisions regarding their diets. These include a further subdivision or categorization of NPBFs, which currently fall mainly in the ultraprocessed (hence, “unhealthy”) food category. Furthermore, standardized and verifiable environmental assessments of NPBFs are needed to compare foods with regard to their environmental footprints. Finally, more research on the short- and longer-term health effects of NPBFs is urgently required to facilitate informed decision-making on the inclusion of NPBFs as part of a wider net-zero and health strategy.

## Supplementary Material

nuae031_Supplementary_Data

## Supporting Information

The following supporting information is available through the online version of this article at the publisher’s website:

Supplementary file 3.

*Box 1* Key Definitions

- **Novel plant-based foods (NPBFs):** Acknowledging differences in terminology for NPBFs, for the purpose of this review, the term *novel plant-based foods* is used to describe plant-based (PB) drinks and PB meat, cheese, eggs, and yogurt alternatives that are of plant or fungal origin and designed to directly replace or mimic animal-based foods. This definition includes fungi-based foods (ie, mycoprotein) that biologically do not belong to the plant kingdom but are typically “designed” similarly to NPBFs as a direct replacement for animal-based foods. Here, the term excludes tofu, tempeh, and seitan because although these might be novel to some high-income settings, they have been part of traditional Asian diets for centuries and, hence, are not subject to the same challenges and evidence gap as NPBFs.
- **Ultraprocessed:** Foods that have undergone a series of industrial techniques and processes
- **Minimally processed plant-based foods:** Plant-based whole foods such as nuts, seeds, cereals, and legumes
