# Supplementary material for: Mapping the evidence of novel plant-based foods: a systematic review of nutritional, health, and environmental impacts in high-income countries
Source: Nutr Rev. 2024 Apr 25;83(7):e1626–46. doi: 10.1093/nutrit/nuae031 (PMC12166169; doi:10.1093/nutrit/nuae031)
Supplement: nuae031_Supplementary_Data [file nuae031_supplementary_data.zip › nuae031_Supplementary_Data/Supplementary1_NajeraEspinosa.docx]

Supplementary Materials 1 for

**Mapping the evidence of novel plant-based foods: A systematic literature review of nutritional, health, and environmental impacts in high-income countries**

Sarah Nájera Espinosa^*^, Genevieve Hadida, Anne Jelmar Sietsma, Carmelia Alae-Carew, Grace Turner, Rosemary Green, Silvia Pastorino, Roberto Picetti and Pauline Scheelbeek*

*Corresponding author email: [sarah.najeraespinosa@lshtm.ac.uk](mailto:sarah.najeraespinosa@lshtm.ac.uk); [pauline.scheelbeek@lshtm.ac.uk](mailto:pauline.scheelbeek@lshtm.ac.uk)

Published in Nutrition Reviews

Table of Contents

[1. Supplementary Background Information 2](#_Toc163725403)

[1.1. Supply and demand of novel plant-based foods 2](#_Toc163725404)

[2. Supplementary Methods 2](#_Toc163725405)

[2.1. Supplementary peer-reviewed literature search 2](#_Toc163725406)

[2.2. Assessment of robustness and relevance of studies 4](#_Toc163725407)

[2.3. Supplementary grey literature search 4](#_Toc163725408)

[2.4. Supplementary selection of nutrients 6](#_Toc163725409)

[2.5. Supplementary data analysis and assumptions 6](#_Toc163725410)

[2.6. Supplementary query searches per database 7](#_Toc163725411)

[2.7. Supplementary fruit, vegetable, legumes, and nut content in novel plant-based foods 11](#_Toc163725412)

[2.8. Prisma 2020 Checklist & abstract checklist 12](#_Toc163725413)

[3. Supplementary Results 14](#_Toc163725414)

[3.1. Supplementary systematic search results-country characteristics of the included studies 14](#_Toc163725415)

[3.2. Supplementary nutrient composition of novel plant-based foods 17](#_Toc163725416)

[3.3. Supplementary health impacts and risk factors of novel plant-based foods 19](#_Toc163725417)

[3.4. Supplementary environmental impacts of novel plant-based foods 23](#_Toc163725418)

[3.5. Supplementary novel plant-based foods fruit, vegetable, legumes, and nut content 26](#_Toc163725419)

[3.6 Results of the assessment of robustness and relevance of studies 27](#_Toc163725420)

[4 Supplementary references 30](#_Toc163725421)

Abbreviations

Animal-based foods ABFs

Greenhouse gas emissions GHGE

Land use LU

Plant-based PB

Novel plant-based foods NPBFs

Water footprint WF

# Supplementary Background Information

### Supply and demand of novel plant-based foods

In the UK, between 2018 and 2020, sales value and volume of different Novel plant-based foods (NPBFs) categories increased for virtually all NPBF products, by 27-165% and 21-154%, respectively.^1^ In Europe, NPBF companies such as Heura and THIS, have also reported sales growth of more than 200% in the past two years.^2,3^ More recently, some evidence suggests that this rate of increase may have attenuated, at least for some products or sub-markets such as plant-based (PB) meats, with one report in the USA indicating that whilst more and more people join the NPBF “consumer group” out of curiosity, 1 in 5 former consumers reported to have stopped buying NPBFs in 2023 due to the higher cost and undesirable taste compared to meats.^4^ Furthermore, companies such as Beyond Meat, Quorn and Meatless Farms have reported net losses in 2022 and 2023 as compared to their 2018-2019 sales.^5,6^ Although this may seem negative for NPBF manufacturers, sales of supermarket own label PB meat alternatives have grown by 14%, while volume has increased by 6% in the last year, potentially due to the lower cost.^5^ While other categories such as PB dairy alternatives continue to increase in high-income countries. For example, in the USA, sales of PB drink alternatives grew by 19% between 2019 to 2022 and PB eggs grew by 611% during the same period.^7^

# Supplementary Methods

### Supplementary peer-reviewed literature search

Three key concepts were combined in the search strategy: NPBFs, health, and environment. Health and environmental search terms were developed by adapting the search terms from the Rockefeller Lancet Commission on Planetary Health^8^, Jarmul et al.^9^ and Alae-Carew et al.^10^; and following discussions with experts and help from the London School of Hygiene & Tropical Medicine information scientists with longstanding expertise in systematic literature searches. The five databases of scientific publications were systematically searched using the London School of Hygiene & Tropical Medicine institutional access. The search strategy was first developed and refined using Medline-Ovid, then minor adjustments were applied for the remaining databases.

Database searching was completed by one individual (SNE). Duplicates articles were removed automatically by a reference citation manager (EndNote) and then screened manually by title and page number by one reviewer (SNE). Titles, abstracts and full-text considered the quality criteria described in Table S1. Only articles containing data on NPBF reporting data on at least one of the following were included: nutrient composition, environmental footprints, and environmental and health impacts; in high-income countries [following the World Bank Classification^11^]. These outcomes were selected because NPBF are novel foods that people are already consuming on a daily basis, and we lack an overview of their health and environmental evidence. Nutritional or environmental outcomes from composite dishes and/or meals were not assessed, because assessing those outside the NPBF field is already challenging. Given the limited number of health studies and the complexity of elaborating dietary studies with humans, studies assessing NPBF within meals or diets when assessing health outcomes were included. Ingredients used to elaborate NPBF [e.g., PB flours, PB hydrolysed protein, and protein isolate], were also excluded from the review for more accurate estimations. Articles outside of the scope of this study were those focusing on feed, ABFs, waste, biofuels, supplements, animal studies, infant formula, and PB whole foods. Lastly, PB diets without segregation of NPBFs and modelled studies without individual data on NPBFs were excluded.

Following the selection criteria, titles were screened manually by three reviewers (SNE, GH, CAC). Given the large number of results abstracts were screened manually and independently by two reviewers (SNE, GH) with the support of a supervised machine learning (Support Vector Machine^12^ through Scikit Learn^13^ where the manual classifications of the reviewers on a subset of the data were used to train the model, which learned to mimic decisions made by the (human) screener to identify abstracts that were likely to be relevant. This approach is described elsewhere in detail.^14^ Abstracts highlighted as likely relevant were then screened manually to avoid false positives. Full text screening and data extraction was completed manually and independently by two reviewers (SNE, GT). When there were discrepancies on either title, abstract screening, or full-text screening and extraction, they were discussed.

Authors were contacted to clarify unclear data or units, to retrieve data and articles that were not available, to request individual data (if possible) and requested main ingredient or food groups when summary tables were published without this information.

**Table S1: Inclusion and exclusion criteria for peer-reviewed papers and grey literature.**

| **Search stage** | **Inclusion Criteria** | **Exclusion Criteria** |
| --- | --- | --- |
| **Title & abstract** | - Any study containing data on single novel PB foods on at least one of the following: nutrient composition, environmental footprints, environmental and health impacts - Any population - Published in the last 6-7 years - Any language | - Does not mention nutrient composition, environmental footprints, and/or environmental and health impacts of alternative foods - Studies analysing infant formula PB alternatives - Minimally processed plant-based foods (e.g., nuts & seeds, legumes, grains etc.) and other traditional plant-based products (e.g., tofu, tempeh, and seitan) - Novel PB foods in diets or composite dishes/meals* |
| **Full-text** | - Studies from high income countries - Studies with clear units - Experimental and observational studies with novel PB foods currently sold in high-income countries (e.g., PB cheese alternatives, PB drinks, PB meats) | - Review articles - Plant-based creamer, cream, pate, buttermilk, and butter - In-vitro studies and animal studies - Single ingredients such as insect flour, plant-based protein isolate, plant-based flours, plant-based protein hydrolyzed without a specified novel PB food product - Novel PB foods used as single ingredients (e.g., cultured fat, flavouring ingredients) - Minimally processed plant-based foods - Grey literature: Nutrient data from food composition tables and manufacturers website |
| *For health studies composite dishes/meals were included only if novel PB food product were specifically measured without the inclusion of other minimally processed plant-based foods and/or other traditional plant-based products  PB: plant-based | | |

### Assessment of robustness and relevance of studies

In order to assess robustness and relevance, the following Table S2 shows the modified checklist from the Critical Appraisal Skills Programme for randomised controlled trials.^15^ If ‘yes’, studies were given a 1 and if ‘no’ they were given a 0.

**Table S2: Modified CASP checklist**

| **#** | **Criterion Description** | **Issues Considered** |
| --- | --- | --- |
| **1** | Clear study description | - Did the authors provide a clear description and justification of the foods evaluated? - Did the authors provide a clear description of (combination of) environmental, health and/or nutritional outcomes? |
| **2** | Appropriate comparison group/situation | - Were foods under the “intervention” [plant-based product(s)] compared to an appropriate and comparable baseline group/situation [animal-based product(s)]? |
| **3** | Clear methods description | - Were the methods clearly described? (e.g., consider exposure and/or outcome) |
| **4** | Rigorous and clearly described analysis | - Are sufficient data presented to support the findings? - Were analyses described in detail? (Could they be repeated by someone not involved in the study) - Did the researchers critically examine their potential biases during measurement, analysis and selection of data for presentation? |
| **5** | Funding | - Was the study funded only by research/governmental institution? (If not stated give 0; if funded by both research/governmental institution and industry give 0) |
| For inclusion in meta-analysis: | | |
| **6** | Precision of measure of effect | - Are the confidence limits, standard deviation and/or uncertainty presented by the authors?   or   - Are the medians and interquartile ranges presented by the authors? |

### Supplementary grey literature search

Results from Google search were exported using the plug-in SEOquake (Table S3). PDFs and academic articles were excluded as they were covered with the methods described above. The text from the remaining webpages were scraped using Beautiful Soup. Since grey literature does not contain structured abstracts like peer-reviewed literature, a pre-trained artificial intelligence text summarization pipeline from Hugging Face was adapted,^16^ and the computer was instructed to create a summary, which was then used to screen each web link. Food composition tables were not used to extract nutritional data. Nutritional data from manufacturers websites were extracted only if a product reported environmental data. The screening process was then followed in the same way as for the peer-reviewed literature.

**Table S3: Google advanced manual searches.**

|  | **Google advance search** | **Number of links** |
| --- | --- | --- |
| **Search #1** | | 104 |
| *All these words* | “environment*” or “nutrition*” or health or nutrient* or “plant-based” |  |
| *This exact word of phrase* | “Alternative protein*” |  |
| *Narrow results by* | Any language, any region, anytime, anywhere in the page, any format, not filtered by license |  |
| *Date of search* | 5 September 2022 |  |
| **Search #2** | | 100 |
| *All these words* | environment* or nutrition* or health or nutrient* or “Novel food*” or “Alternative protein*” or “plant rich” or “plant food*” or “plant protein” or “plant burger” |  |
| *This exact word of phrase* | “Plant based” |  |
| *Narrow results by* | Any language, any region, anytime, anywhere in the page, any format, not filtered by license |  |
| *Date of search* | 5 September 2022 |  |
| **Search #3** | | 104 |
| *All these words* | environment* or nutrition* or health or nutrient* or “Plant based” |  |
| *This exact word of phrase* | “Novel food”* |  |
| *Narrow results by* | Any language, any region, anytime, anywhere in the page, any format, not filtered by license |  |
| *Date of search* | 5 September 2022 |  |

Manual searches of relevant websites from the top NPBF producers in the UK and USA from the 29^th^ of June 2022 (Table S4).

**Table S4: Relevant websites from the top novel plant-based food producers in the UK and USA.**

| **Search on Google** | **Retrieved top 3 websites** | **Identified number of websites** |
| --- | --- | --- |
| **“top plant-based food companies UK”** | |  |
|  | <https://viable.earth/plant-based-food/top-plant-based-food-companies-in-the-uk/> | 14 |
|  | <https://meticulousblog.org/top-10-companies-in-plant-based-food-market/> | 10 |
|  | <https://www.veganrecipeclub.org.uk/articles/big-list-vegan-food-companies/> | 39 |
| **“top plant-based food companies in USA”** | |  |
|  | <https://investingnews.com/daily/life-science-investing/top-plant-based-food-stocks/> | 10 |
|  | <https://meticulousblog.org/top-10-companies-in-plant-based-food-market/> | 10 |
|  | <https://www.nxtaltfoods.com/news/articles/markets-investing/the-ultimate-guide-to-the-top-15-us-plant-based-fast-food-chains/> | 15  Excluded: out of scope |

Manual search of articles and reports from other relevant websites from the 10^th^ of February 2022 (Table S5).

**Table S5: Search of articles and reports from relevant websites.**

| **Website** | **Query (if apply)** | **Results** |
| --- | --- | --- |
| [ClinicalTrials.gov](https://clinicaltrials.gov/) | *“plant-based” OR “plant based” OR “meat alternatives” OR “Dairy alternatives” OR “plant-based diet” OR “vegan diet” OR “vegetarian diet” OR “novel food” OR “Alternative protein” OR “meat analogue” OR “Meat analog”* | 442 articles and links |
| [Food frontier](https://www.foodfrontier.org) |  | 9 reports |
| [The World Economic Forum](https://www.weforum.org/) | “plant-based”; alternative proteins | 18 (2 reports and 16 links) |
| [Food tech + connect newsletter](https://mailchi.mp/foodtechconnect/thrive-market-explores-ipo-kosher-lab-grown-pork-more-2998347?e=90359e0839) |  | 0 |
| [The Good Food Institute](https://www.ecosia.org/search?q=the%20good%20food%20institute&tts=st_asaf_macos) |  | 3 (2 reports and 1 link) |
| [Plant based food association](https://www.plantbasedfoods.org/marketplace/certification/) |  | 0 |
| [Proveg International](https://proveg.com/plant-based-food-and-lifestyle/vegan-alternatives/) |  | 6 (1 report and 5 links) |
| [New food magazine](https://www.newfoodmagazine.com) | “plant-based” | 1 report |
| [Green queen](https://www.greenqueen.com.hk/category/cellbased-protein-cultivated-meat/) |  | 1 report |

### Supplementary selection of nutrients

Nutrients of interest were those associated with the highest burden of disease in high-income countries, and of relevance to UK government advisory bodies.^17,18^ Macronutrients included in the analysis were saturated fat, total sugar, energy, and fibre. Although protein in NPBFs is greatly debated in the scientific community compared to its animal counterparts, protein was not included as a main component in the analysis. Given that protein deficiency is not one of the highest burdens of disease in high-income countries, and instead of lacking protein in the diet,^19,20^ there is an excess of consumption, particularly red meat.^21^ Nevertheless, data on this macronutrient and total fat can be found in supplementary material (see Supplementary file 2 for detailed nutrient information disaggregated by main ingredient). The selection of micronutrients was based on the probability of certain micronutrient deficiencies when shifting to inadequate plant-based diets (e.g., calcium, iodine, iron, and vit B12).^22-24^

### Supplementary data analysis and assumptions

To facilitate data analysis, all food products were standardized to the same nutritional/health/environmental unit and expressed per 100g/100ml of product. PB drinks specific gravity was assumed to be equal to 1.00, based on specific gravity values from soy drink sweetened (1.03) and unsweetened (0.99) from the McCance and Widdowson’s Food Composition Table (FCT).^25^ Energy was converted to kcal considering that 1 kJ = 0.23901 kcal, and data per 8oz were transformed to 100ml considering that 1 fluid ounce = 29.57 ml. Values that reported smaller (<) values were rounded to the closest highest number (i.e., <0.05 = 0.05). Values that reported ‘not detected,’ ‘nor reported’, ‘no nutritional data’ or ‘*’ or ‘-’ were kept as empty cells. Finally, if ranges were reported, the mean was calculated.

For studies reporting environmental and nutrient outcomes with a single comparable ABF (e.g., bovine milk for PB drinks), the ABF was used as the baseline value in each respective study. If a study reported more than one product and did not specify their respective baseline (i.e., beef, chicken & pork), an average was taken as a baseline. The direction and percentage difference of environmental outcomes were extracted for each measurement [greenhouse gas emissions (GHGE), land use (LU) and blue-water footprint (WF)] comparing a single PB food with its animal counterpart (baseline). For environmental studies reporting GHGE without baseline, we used Poore & Nemecek^26^ data as baseline (resampled, randomized data with retail weights – IPCC 2013). An average of all types of meat [i.e., bovine meat from dairy and beef herds, lamb & mutton, poultry, and pig meat] was used for the meat & poultry category; while for milk and cheese only one value was reported. Environmental studies reporting LU and WF without baseline, were excluded for this analysis since methods and system boundaries were very different and hence there were limited possibilities to pool the data. For grey literature references from manufacturers reporting environmental data without nutrient data, food labels from the manufacturer’s website were extracted if the exact name of the product and brand was reported. For nutritional studies without baseline, the McCance and Widdowson’s FCT was used as baseline.^25^ Studies that did not describe life cycle assessment methods were excluded from the analysis.

Due to the clustered nature of the data (i.e., various products within a single paper), descriptive statistics were used analyse data. All plots and statistical analyses were performed in Rstudio 4.1.3.^27^

### Supplementary query searches per database

Initial query search used to find relevant articles for inclusion in the systematic.

**Table S6: Query searches for all databases used to find relevant articles for inclusion in the systematic review on the 29 of June 2022.** Each database contains the number of queries executed for each search (i.e., Search # for all databases and S# for Greenfile). Truncations and proximity searches remained the same for each database: Medline, Embase & Global health = adj#; Greenfile = W#; Web of Science = near/#.

| Search # | Query | Results from 29 June 2022 |
| --- | --- | --- |
| MEDLINE (Ovid) | | |
| 1 | ((Alga* or Seaweed* or Insect* or Fungi* or “culture grown” or “lab grown” or cultivate* or “in vitro” or synthetic* or bioartificial or Frankenstein or artificial*) adj2 (food* or diet* or protein* or meat* or muscle* or burger*)).mp. | 58,380 |
| 2 | (Seitan* or “gluten based” or “wheat based” or Tofu* or tempeh* or Mycoprotein* or “Pea protein*” or “Bean protein*” or “pulse* protein*” or “Chickpea protein*” or “Rice protein*” or “Sunflower seed protein*” or “Coconut protein*” or “Rapeseed protein*”).mp. | 3,566 |
| 3 | (Soy* adj2 (based* or food* or protein*)).mp. | 13,841 |
| 4 | ((Meat or chicken or beef or lamb or pork or turkey or steak* or sausage* or Dairy or milk or cheese or yogurt or butter or cream or Egg* or Seafood or “Sea-food”) adj2 (Alternative* or analog* or substitut* or replacement* or extender* or Emulsion*)).mp. | 4,515 |
| 5 | ((Sustainab* or “Plant based” or Plantbased or “plant rich” or “plant food*” or “plant protein*” or “plant burger” or “plant sourced” or “vegetable sourced”) adj2 (Food* or diet* or product* or ingredient* or protein* or substitut* or analog* or replacement* or meat* or milk* or vegan or vegetarian or flexitarian or pescatarian or extender* or beverage* or Emulsion*)).mp. | 102,789 |
| 6 | (“novel food*” or “Alternative protein*” or “Alternative beverage*”).mp. | 2,510 |
| 7 | (“Non-dairy” adj2 (milk* or beverage*)).mp. | 63 |
| 8 | ((“Dietary behaviour*” or “Dietary behavior*”) adj6 (Alternative* or analog* or substitut* or replacement* or extender*)).mp. | 12 |
| 9 | exp grain proteins/ or exp plant proteins, dietary/ | 17,576 |
| 10 | #1 or #2 or #3 or #4 or #5 or #6 or #7 or #8 or #9 | 188,871 |
| 11 | ((Nutrient* or Nutrition* or food* or micronutrient* or macronutrient*) adj2 (composition or value* or quality* or profile* or analysis or deficiency or availability)).mp. | 78,218 |
| 12 | ((meat or “animal-sourced” or ultra-processed or UPF or dairy or chicken or beef or lamb or pork or turkey or steak* or sausage* or milk or cheese or yogurt or butter or cream or egg*) adj3 (reduc* or decreas* or free)).mp. | 20,001 |
| 13 | (health adj2 (impact* or effect* or deterioration* or improvement* or disease* or risk* or well-being* or wellbeing)).mp. | 211,977 |
| 14 | (BMI or “body mass index” or obesity or “over-weight” or overweight or “under-weight” or underweight or malnutrition or malnour*).mp. | 659,501 |
| 15 | (Anemia or anaemia or hypertension or “blood pressure” or BP or stroke or diabetes or “heart disease” or “cardio-vascular” or “kidney disease” or CKD or cancer or chronic* or “non-communicable disease*”).mp. | 5,392,840 |
| 16 | ((Protein* or Carbohydrate* or Fat or fats or fatty or Mineral* or Vitamin* or Iron or Iodine or Calcium or Zinc or Magnesium or “Dietary mineral*” or Flavonoid* or “ascorbic acid” or Carotenoid* or Phenolic* or Antioxidant*) adj2 (deficien* or shortage* or value* or profile* or quality or score* or availability)).mp. | 175,036 |
| 17 | exp Diet, Vegetarian/ or exp Nutritive Value/ or exp Food Analysis/ | 43,927 |
| 18 | #11 or #12 or #13 or #14 or #15 or #16 or #17 | 6,116,985 |
| 19 | ((climate* or environment*) adj4 (friendly or footprint or “foot-print” or impact* or damage* or greenhouse* or emission* or land* or water* or benefit* or implication* or carbon* or sustain* or nitrogen* or biodivers* or “CO2” or “O3” or ozone* or contamination*)).mp. | 123,445 |
| 20 | (Ecohealth or “Eco-health” or “Planetary health”).mp. | 884 |
| 21 | Climate Change/ or Carbon Footprint/ | 23,845 |
| 22 | 19 or 20 or 21 | 140,658 |
| 23 | 18 or 22 | 6,241,540 |
| 24 | 10 and 23 | 29,461 |
| 25 | 24 not (Animals/ not (Animals/ and Humans/)) | 22,740 |
| 26 | 25 and 2016:2021.(sa_year). | 8,666 |
| EMBASE (OVID) | | |
| 1 | ((Alga* or Seaweed* or Insect* or Fungi* or “culture grown” or “lab grown” or cultivate* or “in vitro” or synthetic* or bioartificial or Frankenstein or artificial*) adj2 (food* or diet* or protein* or meat* or muscle* or burger*)).mp. | 53,432 |
| 2 | (Seitan* or “gluten based” or “wheat based” or Tofu* or tempeh* or Mycoprotein* or “Pea protein*” or “Bean protein*” or “pulse* protein*” or “Chickpea protein*” or “Rice protein*” or “Sunflower seed protein*” or “Coconut protein*” or “Rapeseed protein*”).mp. | 4,248 |
| 3 | (Soy* adj2 (based* or food* or protein*)).mp. | 14,738 |
| 4 | ((Meat or chicken or beef or lamb or pork or turkey or steak* or sausage* or Dairy or milk or cheese or yogurt or butter or cream or Egg* or Seafood or “Sea-food”) adj2 (Alternative* or analog* or substitut* or replacement* or extender* or Emulsion*)).mp. | 4,807 |
| 5 | ((Sustainab* or “Plant based” or Plantbased or “plant rich” or “plant food*” or “plant protein*” or “plant burger” or “plant sourced” or “vegetable sourced”) adj2 (Food* or diet* or product* or ingredient* or protein* or substitut* or analog* or replacement* or meat* or milk* or vegan or vegetarian or flexitarian or pescatarian or extender* or beverage* or Emulsion*)).mp. | 36,666 |
| 6 | (“novel food*” or “Alternative protein*” or “Alternative beverage*”).mp. | 2,575 |
| 7 | (“Non-dairy” adj2 (milk* or beverage*)).mp. | 67 |
| 8 | ((“Dietary behaviour*” or “Dietary behavior*”) adj6 (Alternative* or analog* or substitut* or replacement* or extender*)).mp. | 18 |
| 9 | #1 or #2 or #3 or #4 or #5 or #6 or #7 or #8 | 113,210 |
| 10 | ((Nutrient* or Nutrition* or food* or micronutrient* or macronutrient*) adj2 (composition or value* or quality* or profile* or analysis or deficiency or availability)).mp. | 154,260 |
| 11 | ((meat or “animal-sourced” or ultra-processed or UPF or dairy or chicken or beef or lamb or pork or turkey or steak* or sausage* or milk or cheese or yogurt or butter or cream or egg*) adj3 (reduc* or decreas* or free)).mp. | 22,564 |
| 12 | (health adj2 (impact* or effect* or deterioration* or improvement* or disease* or risk* or well-being* or wellbeing)).mp. | 261,742 |
| 13 | (BMI or “body mass index” or obesity or “over-weight” or overweight or “under-weight” or underweight or malnutrition or malnour*).mp. | 1,089,321 |
| 14 | (Anemia or anaemia or hypertension or “blood pressure” or BP or stroke or diabetes or “heart disease” or “cardio-vascular” or “kidney disease” or CKD or cancer or chronic* or “non-communicable disease*”).mp. | 9,397,675 |
| 15 | ((Protein* or Carbohydrate* or Fat or fats or fatty or Mineral* or Vitamin* or Iron or Iodine or Calcium or Zinc or Magnesium or “Dietary mineral*” or Flavonoid* or “ascorbic acid” or Carotenoid* or Phenolic* or Antioxidant*) adj2 (deficien* or shortage* or value* or profile* or quality or score* or availability)).mp. | 279,704 |
| 16 | exp Diet, Vegetarian/ or exp Nutritive Value/ or exp Food Analysis/ | 59,800 |
| 17 | #10 or #11 or #12 or #13 or #14 or #15 or #16 | 10,384,202 |
| 18 | ((climate* or environment*) adj4 (friendly or footprint or “foot-print” or impact* or damage* or greenhouse* or emission* or land* or water* or benefit* or implication* or carbon* or sustain* or nitrogen* or biodivers* or “CO2” or “O3” or ozone* or contamination*)).mp. | 173,453 |
| 19 | (Ecohealth or “Eco-health” or “Planetary health”).mp. | 1,102 |
| 20 | Climate Change/ or Carbon Footprint/ | 56,877 |
| 21 | #18 or #19 or #20 | 213,003 |
| 22 | #17 or #21 | 10,569,876 |
| 23 | #9 and #22 | 27,160 |
| 24 | #23 not (Animals/ not (Animals/ and Humans/)) | 25,458 |
| 25 | #24 and 2016:2021.(sa_year). | 10,617 |
| GLOBAL HEALTH (Ovid) | | |
| 1 | ((Alga* or Seaweed* or Insect* or Fungi* or “culture grown” or “lab grown” or cultivate* or “in vitro” or synthetic* or bioartificial or Frankenstein or artificial*) adj2 (food* or diet* or protein* or meat* or muscle* or burger*)).mp. | 12,112 |
| 2 | (Seitan* or “gluten based” or “wheat based” or Tofu* or tempeh* or Mycoprotein* or “Pea protein*” or “Bean protein*” or “pulse* protein*” or “Chickpea protein*” or “Rice protein*” or “Sunflower seed protein*” or “Coconut protein*” or “Rapeseed protein*”).mp. | 4,770 |
| 3 | (Soy* adj2 (based* or food* or protein*)).mp. | 10,523 |
| 4 | ((Meat or chicken or beef or lamb or pork or turkey or steak* or sausage* or Dairy or milk or cheese or yogurt or butter or cream or Egg* or Seafood or “Sea-food”) adj2 (Alternative* or analog* or substitut* or replacement* or extender* or Emulsion*)).mp. | 5,088 |
| 5 | ((Sustainab* or “Plant based” or Plantbased or “plant rich” or “plant food*” or “plant protein*” or “plant burger” or “plant sourced” or “vegetable sourced”) adj2 (Food* or diet* or product* or ingredient* or protein* or substitut* or analog* or replacement* or meat* or milk* or vegan or vegetarian or flexitarian or pescatarian or extender* or beverage* or Emulsion*)).mp. | 13,712 |
| 6 | (“novel food*” or “Alternative protein*” or “Alternative beverage*”).mp. | 2,232 |
| 7 | (“Non-dairy” adj2 (milk* or beverage*)).mp. | 82 |
| 8 | ((“Dietary behaviour*” or “Dietary behavior*”) adj6 (Alternative* or analog* or substitut* or replacement* or extender*)).mp. | 10 |
| 9 | exp protein/ | 12,857 |
| 10 | #1 or #2 or #3 or #4 or #5 or #6 or #7 or #8 or #9 | 55,962 |
| 11 | ((Nutrient* or Nutrition* or food* or micronutrient* or macronutrient*) adj2 (composition or value* or quality* or profile* or analysis or deficiency or availability)).mp. | 255,435 |
| 12 | ((meat or “animal-sourced” or ultra-processed or UPF or dairy or chicken or beef or lamb or pork or turkey or steak* or sausage* or milk or cheese or yogurt or butter or cream or egg*) adj3 (reduc* or decreas* or free)).mp. | 16,316 |
| 13 | (health adj2 (impact* or effect* or deterioration* or improvement* or disease* or risk* or well-being* or wellbeing)).mp. | 101,427 |
| 14 | (BMI or “body mass index” or obesity or “over-weight” or overweight or “under-weight” or underweight or malnutrition or malnour*).mp. | 270,075 |
| 15 | (Anemia or anaemia or hypertension or “blood pressure” or BP or stroke or diabetes or “heart disease” or “cardio-vascular” or “kidney disease” or CKD or cancer or chronic* or “non-communicable disease*”).mp. | 768,093 |
| 16 | ((Protein* or Carbohydrate* or Fat or fats or fatty or Mineral* or Vitamin* or Iron or Iodine or Calcium or Zinc or Magnesium or “Dietary mineral*” or Flavonoid* or “ascorbic acid” or Carotenoid* or Phenolic* or Antioxidant*) adj2 (deficien* or shortage* or value* or profile* or quality or score* or availability)).mp. | 106,888 |
| 17 | exp Nutritive Value/ or exp Food Analysis/ | 49,669 |
| 18 | #11 or #12 or #13 or #14 or #15 or #16 or #17 | 1,297,125 |
| 19 | ((climate* or environment*) adj4 (friendly or footprint or “foot-print” or impact* or damage* or greenhouse* or emission* or land* or water* or benefit* or implication* or carbon* or sustain* or nitrogen* or biodivers* or “CO2” or “O3” or ozone* or contamination*)).mp. | 48,362 |
| 20 | (Ecohealth or “Eco-health” or “Planetary health”).mp. | 424 |
| 21 | Climate Change/ or Carbon Footprint/ | 11,881 |
| 22 | #19 or #20 or #21 | 55,493 |
| 23 | #18 or #22 | 1,339,407 |
| 24 | #10 and #23 | 27,612 |
| 25 | #24 not (Animals/ not (Animals/ and Humans/)) | 20,957 |
| 26 | #25 and 2016:2021.(sa_year). | 7,893 |
| GREENFILE (Ebsco) | | |
| S1 | ((Alga* or Seaweed* or Insect* or Fungi* or “culture grown” or “lab grown” or cultivate* or “in vitro” or synthetic* or bioartificial or Frankenstein or artificial*) W2 (food* or diet* or protein* or meat* or muscle* or burger*)) | 1,060 |
| S2 | Seitan* or “gluten based” or “wheat based” or Tofu* or tempeh* or Mycoprotein* or “Pea protein*” or “Bean protein*” or “pulse* protein*” or “Chickpea protein*” or “Rice protein*” or “Sunflower seed protein*” or “Coconut protein*” or “Rapeseed protein*” | 236 |
| S3 | (Soy* W2 (based* or food* or protein*)) | 352 |
| S4 | ((Meat or chicken or beef or lamb or pork or turkey or steak* or sausage* or Dairy or milk or cheese or yogurt or butter or cream or Egg* or Seafood or “Sea-food”) W2 (Alternative* or analog* or substitut* or replacement* or extender* or Emulsion*)) | 207 |
| S5 | ((Sustainab* or “Plant based” or Plantbased or “plant rich” or “plant food*” or “plant protein*” or “plant burger” or “plant sourced” or “vegetable sourced”) W2 (Food* or diet* or product* or ingredient* or protein* or substitut* or analog* or replacement* or meat* or milk* or vegan or vegetarian or flexitarian or pescatarian or extender* or beverage* or Emulsion*)) | 4,653 |
| S6 | “novel food*” or “Alternative protein*” or “Alternative beverage*” | 248 |
| S7 | (“Non-dairy” W2 (milk* or beverage*)) | 6 |
| S8 | ((“Dietary behaviour*” or “Dietary behavior*”) W6 (Alternative* or analog* or substitut* or replacement* or extender*)) | 7,231 |
| S9 | (DE “plant proteins” OR DE “proteins”) | 1,162 |
| S10 | S1 or S2 or S3 or S4 or S5 or S6 or S7 or S8 or S9 | 7,819 |
| S11 | ((Nutrient* or Nutrition* or food* or micronutrient* or macronutrient*) W2 (composition or value* or quality* or profile* or analysis or deficiency or availability)) | 6,796 |
| S12 | ((meat or “animal- sourced” or ultra-processed or UPF or dairy or chicken or beef or lamb or pork or turkey or steak* or sausage* or milk or cheese or yogurt or butter or cream or egg*) W3 (reduc* or decreas* or free)) | 654 |
| S13 | (health W2 (impact* or effect* or deterioration* or improvement* or disease* or risk* or well- being* or wellbeing)) | 25,337 |
| S14 | (BMI or “body mass index” or obesity or “over-weight” or overweight or “under- weight” or underweight or malnutrition or malnour*) | 3,511 |
| S15 | (Anemia or anaemia or hypertension or “blood pressure” or BP or stroke or diabetes or “heart disease” or “cardio-vascular” or “kidney disease” or CKD or cancer or chronic* or “non- communicable disease*”) | 34,389 |
| S16 | ((Protein* or Carbohydrate* or Fat or fats or fatty or Mineral* or Vitamin* or Iron or Iodine or Calcium or Zinc or Magnesium or “Dietary mineral*” or Flavonoid* or “ascorbic acid” or Carotenoid* or Phenolic* or Antioxidant*) W2 (deficien* or shortage* or value* or profile* or quality or score* or availability)) | 1,701 |
| S17 | (DE “veganism” or DE “vegans” or DE “Vegetarian foods”) or (DE “Plant-based diet”) | 86 |
| S18 | #11 or #12 or #13 or #14 or #15 or #16 or #17 | 65,447 |
| S19 | ((climate* or environment*) W4 (friendly or footprint or "foot print" or impact* or damage* or greenhouse* or emission* or land* or water* or benefit* or implication* or carbon* or sustain* or nitrogen* or biodivers* or "CO2" or "O3" or ozone* or contamination*)) | 121,172 |
| S20 | (Ecohealth or "Eco- health" or "Planetary health") | 1,583 |
| S21 | (DE “Climate Change” or DE “Ecological impact”) | 50,165 |
| S22 | #S19 or #S20 or #S21 | 159,175 |
| S23 | #S18 or #S22 | 215,160 |
| S24 | #S10 and #23 | 2,698 |
| S25 | #10 and #23 **Limiters** – Publication Date: 20160101-20211231 | 1,246 |
| WEB OF SCIENCE | | |
| 1 | TS=(((Alga* or Seaweed* or Insect* or Fungi* or "culture grown" or "lab grown" or cultivate* or "in vitro" or synthetic* or bioartificial or Frankenstein or artificial*) near/2 (food* or diet* or protein* or meat* or muscle* or burger*)))  *Indexes=SCI-EXPANDED, SSCI, A&HCI, CPCI-S, CPCI-SSH, ESCI Timespan=All years* | [65,003](https://www.webofscience.com/wos/woscc/summary/a69b7f5c-b2bd-4b18-83a7-9d000f600d71-40b32974/relevance/1) |
| 2 | TOPIC: ((Seitan* or "gluten based" or "wheat based" or Tofu* or tempeh* or Mycoprotein* or "Pea protein*" or "Bean protein*" or "pulse* protein*" or "Chickpea protein*" or "Rice protein*" or "Sunflower seed protein*" or "Coconut protein*" or "Rapeseed protein*") )  *Indexes=SCI-EXPANDED, SSCI, A&HCI, CPCI-S, CPCI-SSH, ESCI Timespan=All years* | [8,629](https://www.webofscience.com/wos/woscc/summary/638169ba-02e7-4243-818a-ffeadd22ea67-40b3297b/relevance/1) |
| 3 | TS=((Soy* near/2 (based* or food* or protein*) ))  *Indexes=SCI-EXPANDED, SSCI, A&HCI, CPCI-S, CPCI-SSH, ESCI Timespan=All years* | [26,938](https://www.webofscience.com/wos/woscc/summary/9198f196-6362-4120-925f-e588ad4693a4-40b32980/relevance/1) |
| 4 | TS=(((Meat or chicken or beef or lamb or pork or turkey or steak* or sausage* or Dairy or milk or cheese or yogurt or butter or cream or Egg* or Seafood or "Sea-food") near/2 (Alternative* or analog* or substitut* or replacement* or extender* or Emulsion*) ))  *Indexes=SCI-EXPANDED, SSCI, A&HCI, CPCI-S, CPCI-SSH, ESCI Timespan=All years* | [10,815](https://www.webofscience.com/wos/woscc/summary/fdfc12f8-aac6-4330-9804-0a6400763955-40b32983/relevance/1) |
| 5 | TS=(((Sustainab* or "Plant based" or Plantbased or "plant rich" or "plant food*" or "plant protein*" or "plant burger" or "plant sourced" or "vegetable sourced") near/2 (Food* or diet* or product* or ingredient* or protein* or substitut* or analog* or replacement* or meat* or milk* or vegan or vegetarian or flexitarian or pescatarian or extender* or beverage* or Emulsion*) ))  *Indexes=SCI-EXPANDED, SSCI, A&HCI, CPCI-S, CPCI-SSH, ESCI Timespan=All years* | [52,361](https://www.webofscience.com/wos/woscc/summary/52c52d83-7c82-4200-b3c4-6e4b6612c4cf-40b32989/relevance/1) |
| 6 | TOPIC: (("novel food*" or "Alternative protein*" or "Alternative beverage*"))  *Indexes=SCI-EXPANDED, SSCI, A&HCI, CPCI-S, CPCI-SSH, ESCI Timespan=All years* | [4,663](https://www.webofscience.com/wos/woscc/summary/4d6027f4-5366-4c29-93b1-915487c62f2f-40b32990/relevance/1) |
| 7 | TS=(("Non-dairy" near/2 (milk* or beverage* or emulsion*)))  *Indexes=SCI-EXPANDED, SSCI, A&HCI, CPCI-S, CPCI-SSH, ESCI Timespan=All years* | [144](https://www.webofscience.com/wos/woscc/summary/d8ffe41a-e4ea-4675-b563-90ce909276fa-40b3299c/relevance/1) |
| 8 | TOPIC: ((("Dietary behaviour*" or "Dietary behavior*") near/6 (Alternative* or analog* or substitut* or replacement* or extender*) ))  *Indexes=SCI-EXPANDED, SSCI, A&HCI, CPCI-S, CPCI-SSH, ESCI Timespan=All years* | [14](https://www.webofscience.com/wos/woscc/summary/9fafecce-f812-4e13-8ec3-5ecc77070ed2-40b329a8/relevance/1) |
| 9 | #8 or #7 or #6 or #5 or #4 or #3 or #2 or #1  *Indexes=SCI-EXPANDED, SSCI, A&HCI, CPCI-S, CPCI-SSH, ESCI Timespan=All years* | [161,437](https://www.webofscience.com/wos/woscc/summary/296d7ce5-ca9a-4751-a779-fea9987d3344-40b329ca/relevance/1) |
| 10 | TS=(((Nutrient* or Nutrition* or food* or micronutrient* or macronutrient*) near/2 (composition or value* or quality* or profile* or analysis or deficiency or availability)))  *Indexes=SCI-EXPANDED, SSCI, A&HCI, CPCI-S, CPCI-SSH, ESCI Timespan=All years* | [163,114](https://www.webofscience.com/wos/woscc/summary/d0339a81-87d9-4643-b2db-82f1d56e9642-40b329db/relevance/1) |
| 11 | TS=(((meat or “animal-sourced” or ultra-processed or UPF or dairy or chicken or beef or lamb or pork or turkey or steak* or sausage* or milk or cheese or yogurt or butter or cream or egg*) near/3 (reduc* or decreas* or free)))  *Indexes=SCI-EXPANDED, SSCI, A&HCI, CPCI-S, CPCI-SSH, ESCI Timespan=All years* | [41,507](https://www.webofscience.com/wos/woscc/summary/2cbac162-0e06-4aa6-ac5d-e0f98578e0f4-40b329e4/relevance/1) |
| 12 | TS=((health) near/2 (impact* or effect* or deterioration* or improvement* or disease* or risk* or well-being* or wellbeing))  Indexes=SCI-EXPANDED, SSCI, A&HCI, CPCI-S, CPCI-SSH, ESCI Timespan=All years | [339,907](https://www.webofscience.com/wos/woscc/summary/7e5ab37b-2dc4-4a0e-82de-157e6c6904d5-40b329f6/relevance/1) |
| 13 | TOPIC: ((BMI or “body mass index” or obesity or “over-weight” or overweight or “under-weight” or underweight or malnutrition or malnour*))  *Indexes=SCI-EXPANDED, SSCI, A&HCI, CPCI-S, CPCI-SSH, ESCI Timespan=All years* | [671,418](https://www.webofscience.com/wos/woscc/summary/82606ec1-b47c-4d79-8530-b058bedf3cad-40b329ff/relevance/1) |
| 14 | TS=((Anemia or anaemia or hypertension or “blood pressure” or BP or stroke or diabetes or “heart disease” or “cardio-vascular” or “kidney disease” or CKD or cancer or chronic* or “non-communicable disease*”))  *Indexes=SCI-EXPANDED, SSCI, A&HCI, CPCI-S, CPCI-SSH, ESCI Timespan=All years* | [6,049,177](https://www.webofscience.com/wos/woscc/summary/4b223ff8-1794-4b68-bf51-ac5c3a61dfe5-40b32a05/relevance/1) |
| 15 | TS=(((Protein* or Carbohydrate* or Fat or fats or fatty or Mineral* or Vitamin* or Iron or Iodine or Calcium or Zinc or Magnesium or “Dietary mineral*” or Flavonoid* or “ascorbic acid” or Carotenoid* or Phenolic* or Antioxidant*) near/2 (deficien* or shortage* or value* or profile* or quality or score* or availability))  *Indexes=SCI-EXPANDED, SSCI, A&HCI, CPCI-S, CPCI-SSH, ESCI Timespan=All years* | [231,165](https://www.webofscience.com/wos/woscc/summary/3e7fcd28-c1da-4291-bdf9-e2c0872dca6a-40b32a0e/relevance/1) |
| 16 | *#15*or*#14*or*#13*or*#12*or*#11*or*#10*  *Indexes=SCI-EXPANDED, SSCI, A&HCI, CPCI-S, CPCI-SSH, ESCI Timespan=All years* | [7,000,917](https://www.webofscience.com/wos/woscc/summary/bde85e21-9da2-4d48-a2af-f0bdbf6021bb-40b32a42/relevance/1) |
| 17 | TS=(((climate* or environment*) near/4 (friendly or footprint or “foot-print” or impact* or damage* or greenhouse* or emission* or land* or water* or benefit* or implication* or carbon* or sustain* or nitrogen* or biodivers* or “CO2” or “O3” or ozone* or contamination*)))  *Indexes=SCI-EXPANDED, SSCI, A&HCI, CPCI-S, CPCI-SSH, ESCI Timespan=All years* | [568,206](https://www.webofscience.com/wos/woscc/summary/5e0de25b-10e5-4958-8550-2905633d822f-40b32a49/relevance/1) |
| 18 | TOPIC: ((Ecohealth or “Eco-health” or “Planetary health” or “climate change”))  *Indexes=SCI-EXPANDED, SSCI, A&HCI, CPCI-S, CPCI-SSH, ESCI Timespan=All years* | [308,700](https://www.webofscience.com/wos/woscc/summary/861ce157-c069-4d14-9bab-1a0fe9da9312-40b32a61/relevance/1) |
| 19 | #18 or #17  *Indexes=SCI-EXPANDED, SSCI, A&HCI, CPCI-S, CPCI-SSH, ESCI Timespan=All years* | [782,933](https://www.webofscience.com/wos/woscc/summary/edfd3ae5-dd94-45a7-a824-64c8b95faea8-40b32a7d/relevance/1) |
| 20 | #19 or #16  *Indexes=SCI-EXPANDED, SSCI, A&HCI, CPCI-S, CPCI-SSH, ESCI Timespan=All years* | [7,733,107](https://www.webofscience.com/wos/woscc/summary/9320afb9-b88b-4d08-a47e-cfd9b5b73a96-40b32abb/relevance/1) |
| 21 | #20 and #9  *Indexes=SCI-EXPANDED, SSCI, A&HCI, CPCI-S, CPCI-SSH, ESCI Timespan=All years* | [42,957](https://www.webofscience.com/wos/woscc/summary/6e3dcd29-fcd3-4e0b-a323-892bbb9191b4-40b32ae8/relevance/1) |
| 22 | #20 and #9  **Refined by: PUBLICATION YEARS:** (2021 OR 2020 OR 2019 OR 2018 OR 2017 OR 2016)  *Indexes=SCI-EXPANDED, SSCI, A&HCI, CPCI-S, CPCI-SSH, ESCI Timespan=All years* | [21,141](https://www.webofscience.com/wos/woscc/summary/33c22c88-f6d6-4c7c-a874-0d0e2eb7f51c-40b32af0/relevance/1) |

### Supplementary fruit, vegetable, legumes, and nut content in novel plant-based foods

For this analysis, each NPBF were classified by product type (i.e., PB drink) and food group based on the main primary ingredient (i.e., nuts & seeds). NPBFs with coconut cream, oil and milk as main primary ingredient were marked to have 0% of fruit content as these ingredients only add total and saturated fat into diets.

In order to select individual NPBFs across all supermarkets, items from FoodDB^28^ were selected by filtering the following supermarket shelves: Bacon alternatives, All dairy free & dairy alternatives, Dairy free cheese alternatives, Vegan cheese alternatives, Meatball alternatives, Dairy free yoghurt alternatives, Plant-based alternatives to yogurt, Dairy alternatives, Vegan meat alternatives, Milk alternatives, Cooked meat alternatives, Vegan dairy alternatives, Soya & dairy alternatives. After selecting these shelves, composite dishes were removed manually using the following keywords under product name: cheesecake, rolls, coffee, dessert, pasta, wellington, rice, custard, ice cream, biomel, seitan, tofu, tempeh, chocolate pots, caramel, flora, falafel, mayonnaise, lactofree, yeast, shake, pasties, spread, creamy, mousse, pudding, trifle, petit, butter, sunflower, cappuccino, macchiato, baking, sponge, crème, chocolate chip, banana blossom, Lactose, cream, sheep, goat, cow, benecol, pastry, veganaise, pasties, actimel.

### Prisma 2020 Checklist & abstract checklist

**Table S7: PRISMA 2020 Checklist.**

| **Section and Topic** | **Item #** | **Checklist item** | **Location where item is reported** |
| --- | --- | --- | --- |
| **TITLE** | | |  |
| Title | 1 | Identify the report as a systematic review. | Pg. 1 |
| **ABSTRACT** | | |  |
| Abstract | 2 | See the PRISMA 2020 for Abstracts checklist– | Supp 1 - Pg. 12-14 |
| **INTRODUCTION** | | |  |
| Rationale | 3 | Describe the rationale for the review in the context of existing knowledge. | Pg. 2-3 |
| Objectives | 4 | Provide an explicit statement of the objective(s) or question(s) the review addresses. | Pg. 2-3 |
| **METHODS** | | |  |
| Eligibility criteria | 5 | Specify the inclusion and exclusion criteria for the review and how studies were grouped for the syntheses. | Pg. 3-4 and Supp. 1 |
| Information sources | 6 | Specify all databases, registers, websites, organisations, reference lists and other sources searched or consulted to identify studies. Specify the date when each source was last searched or consulted. | Pg. 3-4 and Supp. 1 |
| Search strategy | 7 | Present the full search strategies for all databases, registers and websites, including any filters and limits used. | Supp. 1 |
| Selection process | 8 | Specify the methods used to decide whether a study met the inclusion criteria of the review, including how many reviewers screened each record and each report retrieved, whether they worked independently, and if applicable, details of automation tools used in the process. | Supp. 1 |
| Data collection process | 9 | Specify the methods used to collect data from reports, including how many reviewers collected data from each report, whether they worked independently, any processes for obtaining or confirming data from study investigators, and if applicable, details of automation tools used in the process. | Pg. 3-4 and Supp. 1 |
| Data items | 10a | List and define all outcomes for which data were sought. Specify whether all results that were compatible with each outcome domain in each study were sought (e.g. for all measures, time points, analyses), and if not, the methods used to decide which results to collect. | Pg. 3-5 and Supp. 1 |
|  | 10b | List and define all other variables for which data were sought (e.g. participant and intervention characteristics, funding sources). Describe any assumptions made about any missing or unclear information. | Pg. 3-5 and Supp. 1 |
| Study risk of bias assessment | 11 | Specify the methods used to assess risk of bias in the included studies, including details of the tool(s) used, how many reviewers assessed each study and whether they worked independently, and if applicable, details of automation tools used in the process. | Pg. 4-5 and Supp. 1 |
| Effect measures | 12 | Specify for each outcome the effect measure(s) (e.g. risk ratio, mean difference) used in the synthesis or presentation of results. | Pg. 4-5 and Supp. 1 |
| Synthesis methods | 13a | Describe the processes used to decide which studies were eligible for each synthesis (e.g. tabulating the study intervention characteristics and comparing against the planned groups for each synthesis (item #5)). | Pg. 3-5 and Supp. 1 |
|  | 13b | Describe any methods required to prepare the data for presentation or synthesis, such as handling of missing summary statistics, or data conversions. | Pg. 3-5 and Supp. 1 |
|  | 13c | Describe any methods used to tabulate or visually display results of individual studies and syntheses. | Pg. 3-5 and Supp. 1 |
|  | 13d | Describe any methods used to synthesize results and provide a rationale for the choice(s). If meta-analysis was performed, describe the model(s), method(s) to identify the presence and extent of statistical heterogeneity, and software package(s) used. | Pg. 3-5 and Supp. 1 |
|  | 13e | Describe any methods used to explore possible causes of heterogeneity among study results (e.g. subgroup analysis, meta-regression). | - |
|  | 13f | Describe any sensitivity analyses conducted to assess robustness of the synthesized results. | Pg. 4-5 and Supp. 1 |
| Reporting bias assessment | 14 | Describe any methods used to assess risk of bias due to missing results in a synthesis (arising from reporting biases). | Pg. 4-5 and Supp. 1 |
| Certainty assessment | 15 | Describe any methods used to assess certainty (or confidence) in the body of evidence for an outcome. | Pg. 4-5 and Supp. 1 |
| **RESULTS** | | |  |
| Study selection | 16a | Describe the results of the search and selection process, from the number of records identified in the search to the number of studies included in the review, ideally using a flow diagram. | Pg. 5 |
|  | 16b | Cite studies that might appear to meet the inclusion criteria, but which were excluded, and explain why they were excluded. | Figure 1 |
| Study characteristics | 17 | Cite each included study and present its characteristics. | Supp. 1 and Supp. 3 |
| Risk of bias in studies | 18 | Present assessments of risk of bias for each included study. | Pg. 23-15 and Supp. 1 |
| Results of individual studies | 19 | For all outcomes, present, for each study: (a) summary statistics for each group (where appropriate) and (b) an effect estimate and its precision (e.g. confidence/credible interval), ideally using structured tables or plots. | Pg. 13-15; Supp. 2 |
| Results of syntheses | 20a | For each synthesis, briefly summarise the characteristics and risk of bias among contributing studies. | Supp.1 |
|  | 20b | Present results of all statistical syntheses conducted. If meta-analysis was done, present for each the summary estimate and its precision (e.g. confidence/credible interval) and measures of statistical heterogeneity. If comparing groups, describe the direction of the effect. | Pg. 5-15; Supp. 2 |
|  | 20c | Present results of all investigations of possible causes of heterogeneity among study results. | - |
|  | 20d | Present results of all sensitivity analyses conducted to assess the robustness of the synthesized results. | Supp. 1 |
| Reporting biases | 21 | Present assessments of risk of bias due to missing results (arising from reporting biases) for each synthesis assessed. | Supp. 1 |
| Certainty of evidence | 22 | Present assessments of certainty (or confidence) in the body of evidence for each outcome assessed. | Pg. 13-15 and Supp. 1 |
| **DISCUSSION** | | |  |
| Discussion | 23a | Provide a general interpretation of the results in the context of other evidence. | Pg. 15-17 |
|  | 23b | Discuss any limitations of the evidence included in the review. | Pg. 17-18 |
|  | 23c | Discuss any limitations of the review processes used. | Pg. 17-18 |
|  | 23d | Discuss implications of the results for practice, policy, and future research. | Pg. 17 |
| **OTHER INFORMATION** | | |  |
| Registration and protocol | 24a | Provide registration information for the review, including register name and registration number, or state that the review was not registered. | Pg. 3 |
|  | 24b | Indicate where the review protocol can be accessed, or state that a protocol was not prepared. | Pg. 3 |
|  | 24c | Describe and explain any amendments to information provided at registration or in the protocol. | - |
| Support | 25 | Describe sources of financial or non-financial support for the review, and the role of the funders or sponsors in the review. | Pg. 18 |
| Competing interests | 26 | Declare any competing interests of review authors. | Pg. 18 |
| Availability of data, code and other materials | 27 | Report which of the following are publicly available and where they can be found: template data collection forms; data extracted from included studies; data used for all analyses; analytic code; any other materials used in the review. | Pg. 18 |
| *Supp = Supplementary file | | | |

**Table S8: PRISMA 2020 for abstracts checklist.**

| **Section and Topic** | **Item #** | **Checklist item** | **Reported (Yes/No)** |
| --- | --- | --- | --- |
| **TITLE** |  |  |  |
| Title | 1 | Identify the report as a systematic review. | Yes |
| **BACKGROUND** |  |  |  |
| Objectives | 2 | Provide an explicit statement of the main objective(s) or question(s) the review addresses. | Yes |
| **METHODS** |  |  |  |
| Eligibility criteria | 3 | Specify the inclusion and exclusion criteria for the review. | Yes |
| Information sources | 4 | Specify the information sources (e.g., databases, registers) used to identify studies and the date when each was last searched. | Yes |
| Risk of bias | 5 | Specify the methods used to assess risk of bias in the included studies. | Yes |
| Synthesis of results | 6 | Specify the methods used to present and synthesise results. | Yes |
| **RESULTS** |  |  |  |
| Included studies | 7 | Give the total number of included studies and participants and summarise relevant characteristics of studies. | Yes |
| Synthesis of results | 8 | Present results for main outcomes, preferably indicating the number of included studies and participants for each. If meta-analysis was done, report the summary estimate and confidence/credible interval. If comparing groups, indicate the direction of the effect (i.e. which group is favoured). | Yes |
| **DISCUSSION** |  |  |  |
| Limitations of evidence | 9 | Provide a brief summary of the limitations of the evidence included in the review (e.g. study risk of bias, inconsistency and imprecision). | Yes |
| Interpretation | 10 | Provide a general interpretation of the results and important implications. | Yes |
| **OTHER** |  |  |  |
| Funding | 11 | Specify the primary source of funding for the review. | Yes |
| Registration | 12 | Provide the register name and registration number. | No |

# Supplementary Results

### Supplementary systematic search results-country characteristics of the included studies

**Table S9: Articles included in the systematic literature review on the nutrient, health, and environmental outcomes in high-income countries.**

| # | Author (Year) | Reference ID | Plant-based (PB) product | Nutrient | Health | Environment |
| --- | --- | --- | --- | --- | --- | --- |
|  | Ridoutt et al. (2020)^29^ | PR1 | PB drinks | - | - | x |
|  | Vogels’ng-O'Dwyer et al. (2021)^30^ | PR2 | PB drinks | x | - | - |
|  | Pontonio et al. (2020)^31^ | PR3 | PB yogurt | x | - | - |
|  | Ma et al. (2016)^32^ | PR4 | PB drinks | x | - | - |
|  | Richard et al. (2016)^33^ | PR5 | PB drinks | x | - | - |
|  | Cole et al. (2022)^34^ | PR6 | PB meats | x | - | - |
|  | Martinez-Padilla et al. (2020)^35^ | PR7 | PB drinks | x | - | - |
|  | Collard & McCormick (2020)^36^ | PR8 | PB drinks | x | - | - |
|  | Zhang et al. (2020)^37^ | PR9 | PB drinks | x | - | - |
|  | Isidro (2017)^38^ | PR10 | PB drinks | x | - | - |
|  | Jeske et al. (2017)^39^ | PR11 | PB drinks | x | - | - |
|  | Sumner & Burbridge (2020)^40^ | PR12 | PB drinks | x | - | - |
|  | Toribio-Mateas et al. (2021)^41^ | PR13 | PB meats | x | x | - |
|  | Dineva et al. (2021)^42^ | PR14 | PB drinks | - | x | - |
|  | Al-Saedi et al. (2021)^43^ | PR15 | PB cheese | x | - | - |
|  | Seves et al. (2017)^44^ | PR16 | PB drinks & meats | x | - | x |
|  | Sun et al. (2016)^45^ | PR17 | PB drinks | - | x | - |
|  | Angelino et al. (2020)^46^ | PR18 | PB drinks | x | - | - |
|  | Szparaga et al. (2019)^47^ | PR19 | PB drinks | x | - | - |
|  | Tangyu et al. (2021)^48^ | PR20 | PB drinks | x | - | - |
|  | Winans et al. (2020)^49^ | PR21 | PB drinks | - | - | x |
|  | Grant & Hicks (2018)^50^ | PR22 | PB drinks | - | - | x |
|  | Boeck et al. (2021)^51^ | PR23 | PB yogurt | x | - | - |
|  | Roos et al. (2016)^52^ | PR24 | PB drinks | - | - | x |
|  | Smetana et al. (2022)^53^ | PR25 | PB meats | x | - | x |
|  | Liu et al. (2021)^54^ | PR26 | PB meats | x | - | - |
|  | van Vliet et al. (2021)^55^ | PR27 | PB meats | x | - | - |
|  | Crimarco et al. (2020)^56^ | PR28 | PB meats | x | x | - |
|  | Saget et al. (2021)^57^ | PR29 | PB meats | x | - | x |
|  | Mejia et al. (2020)^58^ | PR30 | PB meats | - | - | x |
|  | Detzel et al. (2021)^59^ | PR31 | PB drinks & meats | x | - | x |
|  | Mertens et al. (2020)^60^ | PR32 | PB meats | - | - | x |
|  | Heusala et al. (2020)^61^ | PR33 | PB yogurt | - | - | x |
|  | Goldstein et al. (2017)^62^ | PR34 | PB drinks & meats | x | - | x |
|  | Fresan et al. (2019)^63^ | PR35 | PB meats | x | - | x |
|  | Saerens et al. (2021)^64^ | PR36 | PB meats | - | - | x |
|  | Farsi et al. (2021)^65^ | PR37 | PB meats | x | - | - |
|  | De Marchi et al. (2021)^66^ | PR38 | PB meats | x | - | - |
|  | Kouw et al. (2021)^67^ | PR39 | PB meats | - | x | - |
|  | Curtain & Grafenauer (2019)^68^ | PR40 | PB meats | x | - | - |
|  | Cherta-Murillo & Frost (2021)^68^ | PR41 | PB meats | x | x | - |
|  | Bottin et al. (2016)^69^ | PR42 | PB meats | x | x | - |
|  | Coelho et al. (2021)^70^ | PR43 | PB meats | - | x | - |
|  | Fresan & Rippin (2021)^71^ | PR44 | PB cheese | x | - | - |
|  | Clegg et al. (2021)^72^ | PR45 | PB drinks, yogurt & cheese | x | - | - |
|  | Alessandrini et al. (2021)^73^ | PR46 | PB meats | x | - | - |
|  | Bianchi et al. (2021)^74^ | PR47 | PB meats | - | x | x |
|  | Fresan et al. (2019)^75^ | PR48 | PB meats | - | - | x |
|  | Bryngelsson et al. (2022)^76^ | PR49 | PB meats | x | - | - |
|  | Marques et al. (2021)^77^ | PR50 | PB drinks | x | - | - |
|  | Saget et al. (2021)^78^ | PR51 | PB meats | x | - | x |
|  | Coluccia et al. (2021)^79^ | PR52 | PB drinks | - | - | x |
|  | Boukid et al. (2021)^80^ | PR53 | PB yogurt & cheese | x | - | - |
|  | Pointke & Pawelzik (2022)^81^ | PR54 | PB meats & cheese | x | - | - |
|  | Shen et al. (2019)^82^ | PR55 | PB drinks | x | x | - |
|  | Farsi et al. (2023)^83^ | PR56 | PB meats | x | x | - |
|  | Melville et al. (2023)^84^ | PR57 | PB meats | x | - | - |
|  | Quantis (2020)^85^ | GL1 | PB cheese | - | - | x |
|  | CarbonCloud (2020)^86^ | GL2 | PB drinks | - | - | x |
|  | CarbonCloud (2021)^87^ | GL3 | PB drinks | - | - | x |
|  | CarbonCloud (2021)^88^ | GL4 | PB drinks | - | - | x |
|  | Alpro (2020)^89^ | GL5 | PB drinks | - | - | x |
|  | Ritchie (2022)^90^ | GL6 | PB drinks | - | - | x |
|  | Khan et al. (2019)^91^ | GL7 | PB meats | x | - | x |
|  | Heller & Keoleian (2018)^92^ | GL8 | PB meats | x | - | x |
|  | Dettling et al. (2016)^93^ | GL9 | PB meats | x | - | x |
|  | Reinhardt et al. (2020)^94^ | GL10 | PB drinks, meats, yogurt & cheese | - | - | x |
|  | Kazer et al. (2021)^95^ | GL11 | PB meats | - | - | x |
|  | THIS Co (2022)^96^ | GL12 | PB meats | - | - | x |
|  | Foundation Earth (2022)^97^ | GL13 | PB drinks | - | - | x |
|  | Foundation Earth (2022)^98^ | GL14 | PB drinks | - | - | x |
|  | Foundation Earth (2022)^99^ | GL15 | PB drinks | - | - | x |
|  | Foundation Earth (2022)^100^ | GL16 | PB drinks | - | - | x |
|  | Foundation Earth (2022)^101^ | GL17 | PB drinks | - | - | x |
|  | Foundation Earth (2022)^102^ | GL18 | PB drinks | - | - | x |
|  | Foundation Earth (2022)^103^ | GL19 | PB meats | - | - | x |
|  | Foundation Earth (2022)^104^ | GL20 | PB meats | - | - | x |
|  | Foundation Earth (2022)^105^ | GL21 | PB meats | - | - | x |
|  | Foundation Earth (2022)^106^ | GL22 | PB meats | - | - | x |
|  | Naked (2022)^107^ | GL23 | PB meats | x | - | x |
|  | Naked (2022)^108^ | GL24 | PB meats | x | - | x |
|  | Naked (2022)^109^ | GL25 | PB meats | x | - | x |
|  | Naked (2022)^110^ | GL26 | PB meats | x | - | x |
|  | Naked (2022)^111^ | GL27 | PB meats | x | - | x |
|  | Naked (2022)^112^ | GL28 | PB meats | x | - | x |
|  | Naked (2022)^113^ | GL29 | PB meats | x | - | x |
|  | Naked (2022)^114^ | GL30 | PB meats | x | - | x |
|  | Naked (2022)^115^ | GL31 | PB meats | x | - | x |
|  | Naked (2022)^116^ | GL32 | PB meats | x | - | x |
|  | DUG Drinks (2022)^117^ | GL33 | PB drinks | - | - | x |
|  | Cammelbeeck & Rolvink (2017)^118^ | GL34 | PB meats | - | - | x |
|  | Goudie & Hughes (2022)^119^ | GL35 | PB drinks | x | - | - |
|  | Kazer et al. (2022)^120^ | GL36 | PB meats | - | - |  |
|  | |  |  | **57** | **11** | **52** |

**Figure S1: Distribution of studies included in the systematic literature review.**


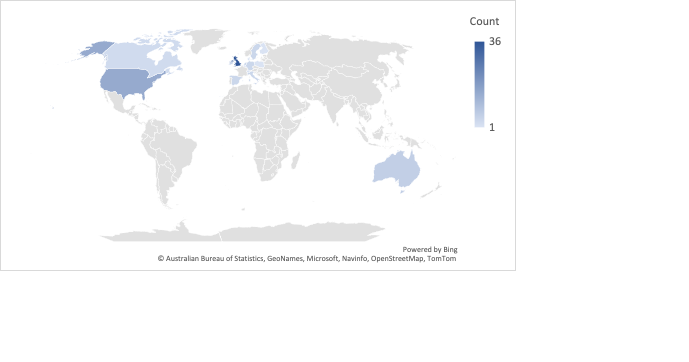


### Supplementary nutrient composition of novel plant-based foods

**Figure S2: Protein content in plant-based (PB) meat and drink alternatives in their respective food group based on main primary ingredient (e.g., predominant or core food item on the ingredient list), compared to meat & poultry and dairy milk.** Data were limited to raw products only. M refers to median of each category.

**
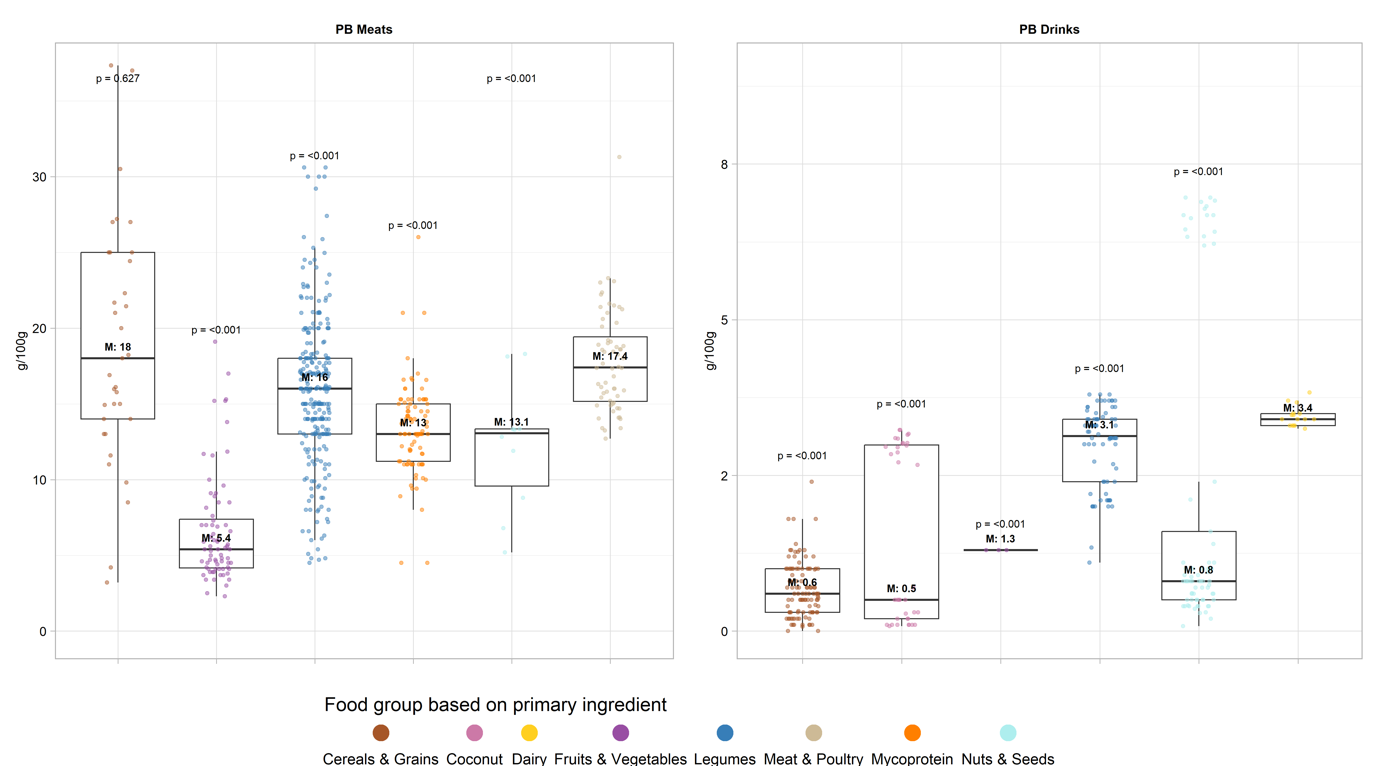
**

**Figure S3: Reduction of environmental outcomes and their associated total sugar of novel plant-based foods compared to baseline (e.g., dairy milk and cheese, meat, poultry) (Expressed in percentage difference).** The y-axis shows the increase or decrease of total sugar in comparison to baseline; and the x-axis shows the reduction (or increase) of the environmental categories. Three environmental categories are reported: greenhouse gas emissions (circles), land use (triangles) and blue water use (squares). Three novel plant-based foods are reported: PB cheese alternatives (brown), PB meat alternatives (purple) and PB drinks (orange).


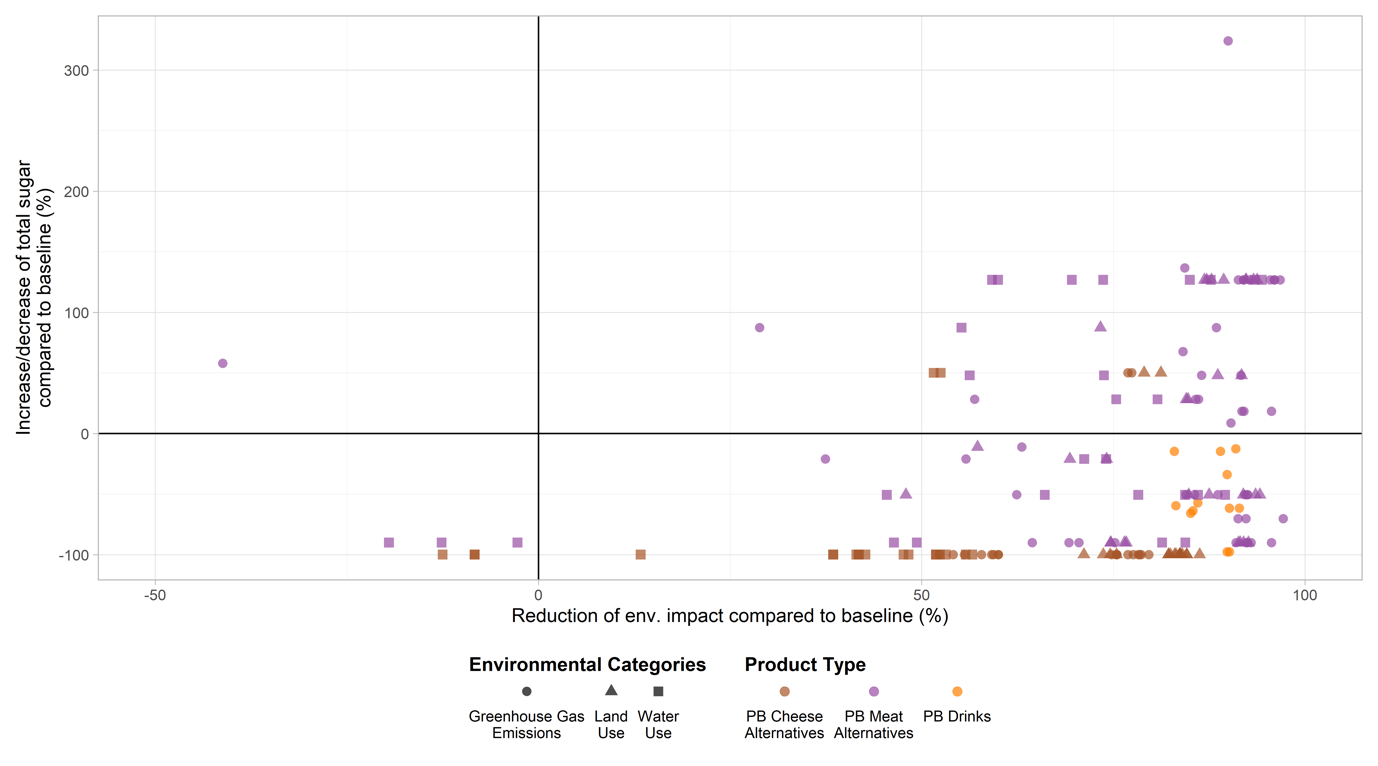


### Supplementary health impacts and risk factors of novel plant-based foods

**Table S10: Evidence on the health impacts/risks of novel plant-based foods.**^41,42,45,56,67,69,70,74,82,83,121^

| Reference | Year of data | Product | Location | n | Duration (days) | Subject | Measured outcomes | Study Design | Wash-out | Intervention | Outcome | Comments |  |
| --- | --- | --- | --- | --- | --- | --- | --- | --- | --- | --- | --- | --- | --- |
| *PB drinks* | | | | | | | | | | | | | |
| Dineva et al. (2021)^42^ | 2016 | PB drink & bovine milk | United Kingdom | 3976 | 4 | Adults | Iodine intake, Urinary iodine concentration | Dietary assessment | No | No | Significant lower iodine intake (exclusively people who consume PB drinks; P < 0.001). Cow milk consumers were iodine sufficient. | Soy and almond drinks were the most frequently reported milk-alternative types.  Funding: Faculty of Health and Medical Sciences at the University of Surrey, UK; Supervisor of paper received an honorarium from Oatly UK and Dairy  UK for outreach purposes |  |
| Shen et al. (2019)^82^ | 2018 | Soy drink & bovine milk | Australia | 8 | 30 | Adults | Enamel mineral changes, saliva calcium and fluoride levels | Double-blind, randomized, cross-over | 1 week in between | Both beverages were tested in random order a follows: 200 ml of soy drink for 15 days & then swap to milk for 15 days more; or the other way around. | Bovine milk resulted in remineralization (significant) and high calcium bioavailability; while soy drink with added sugar promoted enamel demineralization  lower calcium.  No differences between both were found on fluoride levels. | With at least 22 natural teeth; unstimulated whole salivary flow rate of ≥ 0.2 ml/min and chewing gum-stimulated whole salivary flow rate ≥ 1.0ml/min.  Funding: Australian Government Department of Industry and Dairy Australia and the Department of Industry |  |
| Sun et al. (2016)^45^ | 2015 | Soy drink & bovine milk (0.15% fat) | Singapore | 12 | 0.125 | Adults (Young healthy men) | Fasting blood glucose, Plasma amino acids | Randomised, cross-over | 1 week | Control: 50 g of available carbohydrate [white bread (91 g) and water (322ml)], 50 g of available carbohydrate = white bread (58g) + isovolumetric chilled soya milk (322ml) containing approximately 12 g of soya protein | Consumption of soy or bovine milk with carbohydrates induced similar reduction in glycaemic response (through different biological pathways, beyond insulin action). | A day before study: dinner of rice, chicken, and a non-alcoholic beverage. No vigorous physical activity  Funding: Singapore Institute for Clinical Sciences, Agency for Science, Technology and Research (A*STAR) |  |
| *PB meat alternatives* | | | | | | | | | | | | | |
| Crimarco et al. (2020)^56^ | 2020 | PB meat and animal-based foods | USA | 38 | 56 | Adults (Healthy, ≥1 serving meat/d) | Fasting serum trimethylamine-N-oxide (TMAO), LDL-cholesterol, weight, microbiome | Single-site, randomized crossover trial | No | 8 weeks from plant to animal-based foods or from animal to plant-based foods (≥2 servings/d of Plant compared with Animal for 8 wk each, while keeping all other foods and beverages as similar as possible between the 2 phases) | TMAO concentrations were significantly lower overall for Plant (2.7 ± 0.3) than for Animal (4.7 ± 0.9) (P = 0.012), but a significant order effect was observed; LDL cholesterol and weight were lower during plant phase; no IGF-1 differences were observed. | Animal group: no fish was allowed in the 48 h before a study blood draw.  Funding: Beyond Meat (as an unrestricted research gift made to Stanford University) |  |
| Cherta-Murillo & Frost (2021)^121^ | 2013 | Mycoprotein and beef | United Kingdom | 5507 | 4 | Adults | BMI, systolic and diastolic pressure, total cholesterol, triacylglycerides, HDL-cholesterol, LDL-cholesterol, total cholesterol:HDL ratio, C-reactive protein, fasting blood glucose and glycated HbA1c | Cross-sectional analysis | No | No | Mycoprotein intake is associated with lower glycaemic markers and energy density intake, and high fibre (+22.18 %, P < 0.001), energy intake and diet quality scores. | Funding: MRC, BBSRC, NIHR, an Integrative Mammalian Biology (IMB) Capacity Building Award, an FP7- HEALTH- 2009- 241592 EuroCHIP grant, the NIHR Biomedical Research Centre Funding Scheme and an educational no-string grant from Quorn Foods Ltd |  |
| Bottin et al. (2016)^69^ | 2011 | Mycoprotein & Poultry | United Kingdom | 55 | 3 | Adults (Overweight & obese) | Appetite, glucose, insulin, peptide tyrosine-tyrosine (PYY) and glucagon-like peptide-1 (GLP-1) | Single-blinded randomised-controlled trials | No | Consumed 350 g rissoto + a test meal containing low (44 g), medium (88 g) or high (132 g) mycoprotein or isoenergetic chicken meals | Ab libitum mycoprotein intake reduced energy intake (by 10%) and insulin release. The mechanism does not involve changes in PYY and GLP-1. | Funding: Medical Research Council (MRC), Biotechnology and Biological Sciences Research Council (BBSRC), National Institute for Health Research, an Integrative Mammalian Biology (IMB) Capacity Building Award, an FP7-HEALTH-2009-241592 EuroCHIP grant, NIHR Imperial Biomedical Research Centre Funding Scheme; and an educational no string grant from Marlow Foods |  |
| Coelho et al. (2021)^70^ | 2017 | Mycoprotein & animal-based foods | United Kingdom | 20 | 7 | Adults (Young & healthy) | Oral glucose tolerance tests, blood glucose monitoring, fasting plasma analysed using quantitative, targeted NMR-based metabolomics. | Randomised, controlled, parallel-design trial, with participants being randomly allocated | No | Two meals a day: meat/fish-derived dietary protein (n=10) or mycoprotein (n=10) | -No changes in whole-body insulin sensitivity or glycaemic control but changes to plasma lipid composition (primarily consisting of a coordinated reduction in circulating cholesterol-containing lipoproteins). | Overnight fast and refraining from intense exercise and alcohol consumption for at least 24 h  Funding: Marlow Foods Ltd and the College of Life and Environmental Sciences, University of Exeter |  |
| Bianchi et al. (2021)^74^ | 2019 | PB meat & animal-based foods | United Kingdom | 115 | 28 | Adults | Meat & meat substitutes consumption, body weight & body composition, blood pressure, blood lipid profiles (total cholesterol, High-density lipoprotein (HDL) cholesterol, triglycerides, Low-density lipoprotein (LDL) cholesterol, non-HDL cholesterol, LDL:HDL cholesterol ratio), psychosocial variables related to meat consumption, nutritional composition diet | 2-arm, individually randomized controlled trial | No | Behavioural: free meat substitutes, information, recipes, and success stories | Reduced meat consumption by 39 g/d, significant reduction in body weight and reduced greenhouse gas emissions and land use. | Funding: Medical Research Council, Green Templeton College, the NIHR School for Primary Care Research, NIHR Oxford Biomedical Research Centre and the NIHR Applied Research Collaboration |  |
| Kouw et al. (2021)^67^ | 2017 | PB meat & chicken | Netherlands | 24 | 0.208 | Adults (Men, Young & healthy) | Plasma (glucose, insulin, and amino acid concentrations & plasma L-(ring-13C6)-phenylalanine enrichments), and muscle samples (mixed muscle protein-bound L-(ring-13C6)-phenylalanine enrichments & protein signalling) | Parallel, double-blind, randomised controlled trial | No | Consumption of 40 g of protein: either 230 g of a baked lysine-enriched wheat & chickpea protein product or 174 g of baked chicken breast | Ingestion of lysine- enriched, PB meat and chicken increased muscle protein synthesis rates from post-absorptive to postprandial, with no differences between both | 3-days prior trial: Refrain from strenuous physical activity and alcohol consumption  Evening before trial: all participants consumer a pre-packaged standardised meal, followed by overnight fast  Funding: Tereos (Marckolsheim, France) and TiFN (Wageningen, the Netherlands) |  |
| Toribio-Mateas et al. (2021)^41^ | 2021 | PB meat & animal-based foods (red meat/poultry/fish/eggs/cheese dairy) | United Kingdom | 39 | 28 | Adults (Healthy) | Gut microbiome | Randomised controlled trial, pre-and post-intervention assessment | No | Replaced 4 meat-containing meals per week with PB meat alternatives | Positive changes in the gut microbiome: statistically significant changes in the presence of butyrate-producing pathways & a consistent increase in the joint abundances of butyrate-producing taxa | Funding: School of Applied Sciences, London South Bank University (grant number ETH2021-0025) and The Meatless Farm |  |
| Farsi et al. (2023)^83^ | 2019-2020 | Mycoprotein & red and processed red meat | United Kingdom | 20 | 28 | Adults (Healthy men) | Faecal genotoxicity and genotoxins, and changes in gut microbiome composition and activity | Investigator-blind, randomised, crossover | 4 week in-between | 4-week trial: consume 240 g/day red and processed meat for 2 weeks, then a crossover to 240 g/day mycoprotein for 2 weeks | Mycoprotein consumption instead of meat, increased self-reported fibre intake, significantly reduced faecal genotoxicity and genotoxin excretion and increases the abundance of microbial genera associated with gut health benefits in humans. | Funding: part funded by Marlow foods Ltd. And the Northumbria University |  |

### Supplementary environmental impacts of novel plant-based foods

#### Data sources & applied methods of environmental footprint calculations

There were six data providers mentioned as source for footprint calculations in the included studies: Foundation Earth (n=20), Carbon Cloud (n=4), Blonk consultants (n=2), Carbon Trust (n=2), Quantis (n=3), and the University of Michigan (n=1). Nineteen studies did not use a data providers, and two grey literature references were excluded as they did not clearly describe their methodology (as per protocol)^89,96^.

**Table S11: List of environmental categories listed across all studies.**

| # | Author | Reference ID | GHGE | EX | ET | IR | LU | HT | OD | PF | R | PM | WF | AP | B | M | E |
| --- | --- | --- | --- | --- | --- | --- | --- | --- | --- | --- | --- | --- | --- | --- | --- | --- | --- |
|  | Ridoutt et al. (2020)^29^ | PR1 |  |  |  |  | x |  |  |  |  |  |  |  | x | x |  |
|  | Seves et al. (2017)^44^ | PR16 | x |  |  |  | x |  |  |  |  |  |  |  |  |  |  |
|  | Winans et al. (2020)^49^ | PR21 | x | x | x |  |  | x | x | x |  | x | x | x |  |  | x |
|  | Grant & Hicks (2018)^50^ | PR22 | x | x | x |  |  |  |  |  | x |  | x |  |  |  | x |
|  | Roos et al. (2016)^52a^ | PR24 | x | x | x |  |  |  |  |  |  |  |  | x |  |  |  |
|  | Smetana et al. (2022)^53^ | PR25 | x | x |  | x | x | x | x |  |  |  |  | x |  |  |  |
|  | Saget et al. (2021)^57^ | PR29 | x | x | x | x | x | x | x | x | x | x | x | x |  |  | x |
|  | Mejia et al. (2020)^58^ | PR30 | x |  |  |  |  |  |  |  |  |  |  |  |  |  |  |
|  | Detzel et al. (2021)^59^ | PR31 | x |  | x |  | x |  | x | x | x | x | x | x |  |  | x |
|  | Mertens et al. (2020)^60^ | PR32 | x |  |  |  |  |  |  |  |  |  |  |  |  |  |  |
|  | Heusala et al. (2020)^61^ | PR33 | x |  |  |  | x |  |  |  |  |  |  |  |  |  |  |
|  | Goldstein et al. (2017^62^ | PR34 | x |  |  |  | x |  |  |  |  |  | x |  |  |  |  |
|  | Fresan et al. (2019)^63^ | PR35 | x |  |  |  |  |  |  |  |  |  |  |  |  |  |  |
|  | Saerens et al. (2021)^64^ | PR36 |  |  |  |  |  |  |  |  |  |  | x |  |  |  | x |
|  | Bianchi et al. (2021)^74^ | PR47 |  |  |  |  |  |  |  |  |  |  |  |  |  |  |  |
|  | Fresan et al. (2019)^75^ | PR48 |  | x | x |  |  |  |  |  |  |  | x |  |  |  |  |
|  | Saget et al. (2021)^78^ | PR51 | x | x | x | x | x | x | x | x | x | x | x | x |  |  | x |
|  | Coluccia et al. (2021)^79^ | PR52 | x |  |  |  |  |  |  |  |  |  |  |  |  |  |  |
|  | Quantis (2020)^85^ | GL1 | x |  |  |  | x |  |  |  |  |  | x |  |  |  |  |
|  | CarbonCloud (2020)^86^ | GL2 | x |  |  |  |  |  |  |  |  |  |  |  |  |  |  |
|  | CarbonCloud (2021)^87^ | GL3 | x |  |  |  |  |  |  |  |  |  |  |  |  |  |  |
|  | CarbonCloud (2021)^88^ | GL4 | x |  |  |  |  |  |  |  |  |  |  |  |  |  |  |
|  | Alpro (2020)^89^ | GL5 | x |  |  |  |  |  |  |  |  |  | x |  |  |  |  |
|  | Ritchie (2022)^90^ | GL6 | x |  | x |  | x |  |  |  |  |  | x |  |  |  |  |
|  | Khan et al. (2019)^91^ | GL7 | x |  | x |  | x |  |  |  |  |  | x |  |  |  |  |
|  | Heller & Keoleian (2018)^92^ | GL8 | x |  |  |  | x |  |  |  |  |  | x |  |  |  | x |
|  | Dettling et al. (2016)^93^ | GL9 | x | x | x | x | x | x | x |  | x | x | x | x |  |  |  |
|  | Reinhardt et al. (2020)^94^ | GL10 | x |  | x |  |  |  |  |  | x |  | x |  |  |  |  |
|  | Kazer et al. (2021)^95^ | GL11 | x |  |  |  | x |  |  |  |  |  | x |  |  |  |  |
|  | THIS Co (2022)^96^ | GL12 | x |  |  |  |  |  |  |  |  |  | x |  |  |  |  |
|  | Foundation Earth (2022)^97^ | GL13 | x |  | x |  |  |  |  |  |  |  | x |  | x |  |  |
|  | Foundation Earth (2022)^98^ | GL14 | x |  | x |  |  |  |  |  |  |  | x |  | x |  |  |
|  | Foundation Earth (2022)^99^ | GL15 | x |  | x |  |  |  |  |  |  |  | x |  | x |  |  |
|  | Foundation Earth (2022)^100^ | GL16 | x |  | x |  |  |  |  |  |  |  | x |  | x |  |  |
|  | Foundation Earth (2022)^101^ | GL17 | x |  | x |  |  |  |  |  |  |  | x |  | x |  |  |
|  | Foundation Earth (2022)^102^ | GL18 | x |  | x |  |  |  |  |  |  |  | x |  | x |  |  |
|  | Foundation Earth (2022)^103^ | GL19 | x |  | x |  |  |  |  |  |  |  | x |  | x |  |  |
|  | Foundation Earth (2022)^104^ | GL20 | x |  | x |  |  |  |  |  |  |  | x |  | x |  |  |
|  | Foundation Earth (2022)^105^ | GL21 | x |  | x |  |  |  |  |  |  |  | x |  | x |  |  |
|  | Foundation Earth (2022)^106^ | GL22 | x |  | x |  |  |  |  |  |  |  | x |  | x |  |  |
|  | Naked (2022)^107^ | GL23 | x |  | x |  |  |  |  |  |  |  | x |  | x |  |  |
|  | Naked (2022)^108^ | GL24 | x |  | x |  |  |  |  |  |  |  | x |  | x |  |  |
|  | Naked (2022)^109^ | GL25 | x |  | x |  |  |  |  |  |  |  | x |  | x |  |  |
|  | Naked (2022)^110^ | GL26 | x |  | x |  |  |  |  |  |  |  | x |  | x |  |  |
|  | Naked (2022)^111^ | GL27 | x |  | x |  |  |  |  |  |  |  | x |  | x |  |  |
|  | Naked (2022)^112^ | GL28 | x |  | x |  |  |  |  |  |  |  | x |  | x |  |  |
|  | Naked (2022)^113^ | GL29 | x |  | x |  |  |  |  |  |  |  | x |  | x |  |  |
|  | Naked (2022)^114^ | GL30 | x |  | x |  |  |  |  |  |  |  | x |  | x |  |  |
|  | Naked (2022)^115^ | GL31 | x |  | x |  |  |  |  |  |  |  | x |  | x |  |  |
|  | Naked (2022)^116^ | GL32 | x |  | x |  |  |  |  |  |  |  | x |  | x |  |  |
|  | DUG Drinks (2022)^117^ | GL33 | x |  |  |  |  |  |  |  |  |  |  |  |  |  |  |
|  | Cammelbeeck & Rolvink (2017)^118^ | GL34 | x |  |  |  | x |  |  |  |  |  | x |  |  |  |  |
|  | Kazer et al. (2022)^120^ | GL36 | x |  |  |  | x |  |  |  |  |  | x |  |  |  |  |
|  | *Abbreviations: GHGE: Greenhouse gas emissions; EX: Ecotoxicity potential (water & terrestrial); ET: Eutrophication potential (water, marine and terrestrial); IR: Ionising radiation, human health; LU: Land use; HT: Human toxicity (cancer and non-cancer); OD: Ozone depletion; PF: Photochemical ozone formation; R: Resource use (fossil fuels, minerals and metals); PM: Particulate matter emissions (organics and inorganics); WF: Water footprint (use); AP: Acidification potential (water & terrestrial); B: Biodiversity footprint; M: Malnutrition footprint; E: Energy use* | | | | | | | | | | | | | | | | |
|  | ^a^ Data reported was not used in aggregated data by 100g because it was presented on a yearly basis instead of a per-product basis. | | | | | | | | | | | | | | | | |

**Figure S4: Reported greenhouse gas emission for novel plant-based foods and their respective animal-based food comparators.** For meat & poultry, baseline values higher than 4 kg CO_2_ eq/100g were excluded from this figure, including the baseline value of 9.95 kg CO_2_ eq/100g for bovine meat (Beef herd) from Poore & Nemecek (2018). M refers to median of each category.


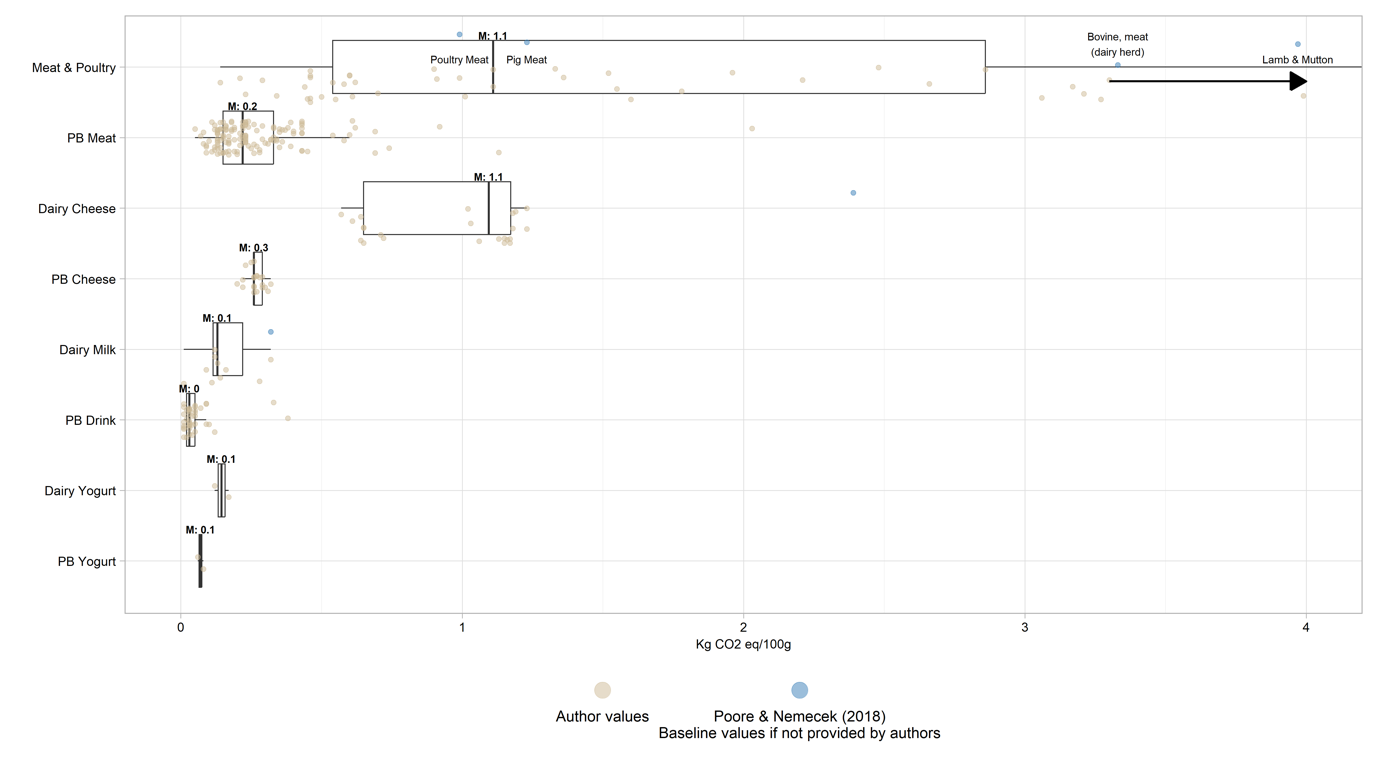


### Supplementary novel plant-based foods fruit, vegetable, legumes, and nut content

Figure S5 shows the fruit, vegetable, legumes, and nut percentage (%) by product and their respective main primary ingredient.

**Figure S5: Estimated fruit, vegetable, legume, and nut content (%) in each novel plant-based foods divided by their respective food group based on main primary ingredient.** Each column shows the type of product (e.g., Plant-based drink) and each row shows the fruit, legumes, nuts, and vegetable content.


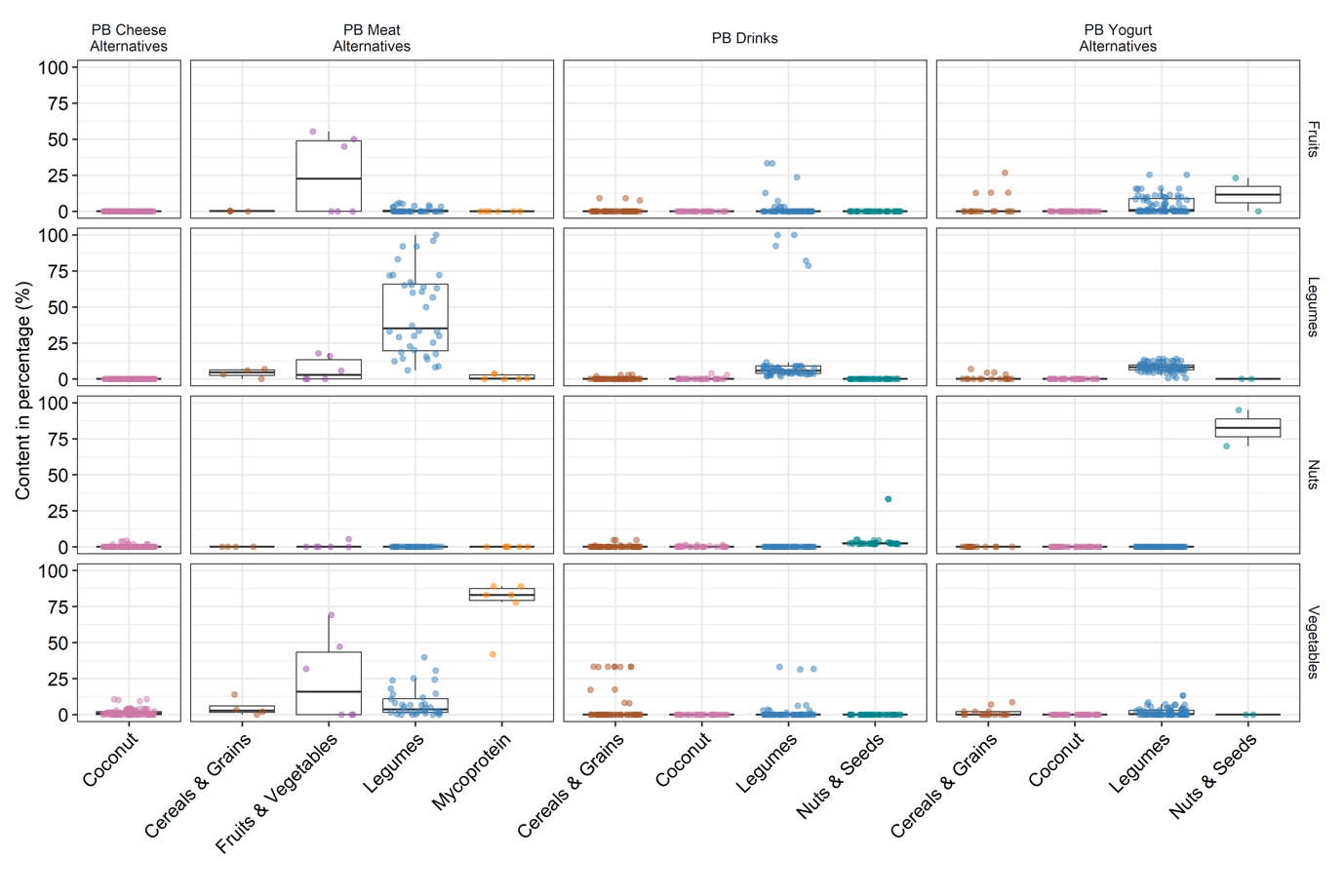


### Results of the assessment of robustness and relevance of studies

**Table S12: Scored studies with the modified CASP checklist.**

| # | Author (Year) | Reference ID | *Criteria 1* | *Criteria 2* | *Criteria 3* | *Criteria 4* | *Criteria 5* | *Criteria 6* | *Total Score* |
| --- | --- | --- | --- | --- | --- | --- | --- | --- | --- |
|  | Ridoutt et al. (2020)^29^ | *PR1* | *1* | *1* | *1* | *1* | *0* | *0* | *4* |
|  | Vogelsang-O’Dwyer et al. (2021)^30^ | *PR2* | *1* | *1* | *1* | *1* | *1* | *0* | *5* |
|  | Pontonio et al. (2020)^31^ | *PR3* | *1* | *0* | *1* | *0* | *0* | *1* | *3* |
|  | Ma et al. (2016)^32^ | *PR4* | *1* | *1* | *0* | *0* | *0* | *0* | *2* |
|  | Richard et al. (2016)^33^ | *PR5* | *1* | *1* | *1* | *1* | *0* | *0* | *4* |
|  | Cole et al. (2022)^34^ | *PR6* | *1* | *1* | *1* | *1* | *1* | *1* | *6* |
|  | Martinez-Padilla et al. (2020)^35^ | *PR7* | *1* | *1* | *1* | *1* | *1* | *0* | *5* |
|  | Collard & McCormick (2020)^36^ | *PR8* | *1* | *1* | *1* | *0* | *0* | *0* | *3* |
|  | Zhang et al. (2020)^37^ | *PR9* | *1* | *1* | *1* | *1* | *1* | *1* | *6* |
|  | Isidro (2017)^38^ | *PR10* | *1* | *0* | *1* | *0* | *0* | *0* | *2* |
|  | Jeske et al. (2017)^39^ | *PR11* | *1* | *1* | *1* | *1* | *1* | *1* | *6* |
|  | Sumner & Burbridge^40^ | *PR12* | *1* | *1* | *1* | *1* | *0* | *0* | *4* |
|  | Toribio-Mateas et al. (2021)^41^ | *PR13* | *1* | *1* | *1* | *1* | *0* | *0* | *4* |
|  | Dineva et al. (2021)^42^ | *PR14* | *1* | *1* | *1* | *1* | *0* | *1* | *5* |
|  | Al-Saedi et al. (2021)^43^ | *PR15* | *1* | *0* | *1* | *1* | *1* | *0* | *4* |
|  | Seves et al. (2017)^44^ | *PR16* | *1* | *1* | *1* | *1* | *1* | *1* | *6* |
|  | Sun et al. (2016)^45^ | *PR17* | *1* | *1* | *1* | *1* | *1* | *1* | *6* |
|  | Angelino et al. (2020)^46^ | *PR18* | *1* | *0* | *1* | *1* | *1* | *0* | *4* |
|  | Szparaga et al. (2019)^47^ | *PR19* | *1* | *0* | *1* | *1* | *1* | *1* | *5* |
|  | Tangyu et al. (2021)^48^ | *PR20* | *1* | *0* | *1* | *1* | *0* | *0* | *3* |
|  | Winans et al. (2020)^49^ | *PR21* | *1* | *0* | *1* | *1* | *0* | *0* | *3* |
|  | Grant & Hicks (2018)^50^ | *PR22* | *1* | *1* | *1* | *1* | *0* | *0* | *4* |
|  | Boeck et al. (2021)^51^ | *PR23* | *1* | *1* | *1* | *1* | *1* | *0* | *5* |
|  | Roos et al. (2016)^52^ | *PR24* | *1* | *1* | *1* | *1* | *0* | *0* | *4* |
|  | Smetana et al. (2022)^53^ | *PR25* | *1* | *1* | *1* | *1* | *1* | *0* | *5* |
|  | Liu et al. (2021)^54^ | *PR26* | *1* | *1* | *1* | *1* | *1* | *1* | *6* |
|  | van Vliet et al. (2021)^55^ | *PR27* | *1* | *1* | *1* | *1* | *0* | *0* | *4* |
|  | Crimarco et al. (2020)^56^ | *PR28* | *1* | *1* | *1* | *1* | *0* | *0* | *4* |
|  | Saget et al. (2021)^57^ | *PR29* | *1* | *1* | *1* | *1* | *1* | *0* | *5* |
|  | Mejia et al. (2020)^58^ | *PR30* | *1* | *0* | *1* | *1* | *1* | *1* | *5* |
|  | Detzel et al. (2021)^59^ | *PR31* | *1* | *1* | *1* | *1* | *1* | *0* | *5* |
|  | Mertens et al. (2020)^60^ | *PR32* | *1* | *1* | *0* | *1* | *0* | *0* | *3* |
|  | Heusala et al. (2020)^61^ | *PR33* | *1* | *0* | *1* | *1* | *1* | *0* | *4* |
|  | Goldstein et al. (2017)^62^ | *PR34* | *1* | *1* | *1* | *1* | *0* | *0* | *4* |
|  | Fresan et al. (2019)^63^ | *PR35* | *1* | *0* | *1* | *1* | *1* | *1* | *5* |
|  | Saerens et al. (2021)^64^ | *PR36* | *1* | *1* | *1* | *1* | *1* | *0* | *5* |
|  | Farsi et al. (2021)^65^ | *PR37* | *1* | *1* | *1* | *1* | *0* | *0* | *4* |
|  | De Marchi et al. (2021)^66^ | *PR38* | *1* | *1* | *1* | *1* | *1* | *1* | *6* |
|  | Kouw et al. (2021)^67^ | *PR39* | *1* | *1* | *1* | *1* | *0* | *1* | *5* |
|  | Curtain & Grafenauer (2019)^68^ | *PR40* | *1* | *1* | *1* | *1* | *1* | *1* | *6* |
|  | Cherta-Murillo & Frost (2021)^68^ | *PR41* | *1* | *1* | *1* | *1* | *0* | *1* | *5* |
|  | Bottin et al. (2016)^69^ | *PR42* | *1* | *1* | *1* | *1* | *0* | *1* | *5* |
|  | Coelho et al. (2021)^70^ | *PR43* | *1* | *1* | *1* | *1* | *0* | *0* | *4* |
|  | Fresan & Rippin (2021)^71^ | *PR44* | *1* | *1* | *1* | *1* | *1* | *0* | *5* |
|  | Clegg et al. (2021)^72^ | *PR45* | *1* | *1* | *1* | *1* | *1* | *1* | *6* |
|  | Alessandrini et al. (2021)^73^ | *PR46* | *1* | *1* | *1* | *1* | *1* | *1* | *6* |
|  | Bianchi et al. (2021)^74^ | *PR47* | *1* | *1* | *1* | *1* | *1* | *1* | *6* |
|  | Fresan et al. (2019)^75^ | *PR48* | *1* | *1* | *1* | *1* | *1* | *0* | *5* |
|  | Bryngelsson et al. (2022)^76^ | *PR49* | *1* | *1* | *1* | *1* | *1* | *1* | *6* |
|  | Marques et al. (2021)^77^ | *PR50* | *1* | *1* | *1* | *1* | *1* | *1* | *6* |
|  | Saget et al. (2021)^78^ | *PR51* | *1* | *1* | *1* | *1* | *1* | *0* | *5* |
|  | Coluccia et al. (2021)^79^ | *PR52* | *1* | *1* | *1* | *1* | *0* | *0* | *4* |
|  | Boukid et al. (2021)^80^ | *PR53* | *1* | *1* | *1* | *0* | *1* | *0* | *4* |
|  | Pointke & Pawelzik (2022)^81^ | *PR54* | *1* | *1* | *1* | *1* | *1* | *1* | *6* |
|  | Shen et al. (2019)^82^ | *PR55* | *1* | *1* | *1* | *0* | *0* | *1* | *4* |
|  | Farsi et al. (2023)^83^ | *PR56* | *1* | *1* | *1* | *1* | *0* | *1* | *5* |
|  | Melville et al. (2023)^84^ | *PR57* | *1* | *1* | *1* | *1* | *0* | *1* | *5* |
|  | Quantis (2020)^85^ | *GL1* | *1* | *1* | *1* | *0* | *0* | *0* | *3* |
|  | CarbonCloud (2020)^86^ | *GL2* | *1* | *0* | *1* | *0* | *0* | *0* | *2* |
|  | CarbonCloud (2021)^87^ | *GL3* | *1* | *0* | *1* | *0* | *0* | *0* | *2* |
|  | CarbonCloud (2021)^88^ | *GL4* | *1* | *0* | *1* | *0* | *0* | *0* | *2* |
|  | Alpro (2020)^89^ | *GL5* | *0* | *0* | *0* | *0* | *0* | *0* | *0* |
|  | Ritchie (2022)^90^ | *GL6* | *1* | *1* | *0* | *0* | *1* | *0* | *3* |
|  | Khan et al. (2019)^91^ | *GL7* | *1* | *1* | *1* | *1* | *0* | *0* | *4* |
|  | Heller & Keoleian (2018)^92^ | *GL8* | *1* | *1* | *1* | *1* | *0* | *0* | *4* |
|  | Dettling et al. (2016)^93^ | *GL9* | *1* | *1* | *1* | *1* | *0* | *0* | *4* |
|  | Reinhardt et al. (2020)^94^ | *GL10* | *1* | *1* | *1* | *0* | *1* | *0* | *4* |
|  | Kazer et al. (2021)^95^ | *GL11* | *1* | *1* | *1* | *0* | *0* | *0* | *3* |
|  | THIS Co (2022)^96^ | *GL12* | *0* | *1* | *0* | *0* | *0* | *0* | *1* |
|  | Foundation Earth (2022)^97^ | *GL13* | *0* | *0* | *1* | *0* | *0* | *0* | *1* |
|  | Foundation Earth (2022)^98^ | *GL14* | *0* | *0* | *1* | *0* | *0* | *0* | *1* |
|  | Foundation Earth (2022)^99^ | *GL15* | *0* | *0* | *1* | *0* | *0* | *0* | *1* |
|  | Foundation Earth (2022)^100^ | *GL16* | *0* | *0* | *1* | *0* | *0* | *0* | *1* |
|  | Foundation Earth (2022)^101^ | *GL17* | *0* | *0* | *1* | *0* | *0* | *0* | *1* |
|  | Foundation Earth (2022)^102^ | *GL18* | *0* | *0* | *1* | *0* | *0* | *0* | *1* |
|  | Foundation Earth (2022)^103^ | *GL19* | *0* | *0* | *1* | *0* | *0* | *0* | *1* |
|  | Foundation Earth (2022)^104^ | *GL20* | *0* | *0* | *1* | *0* | *0* | *0* | *1* |
|  | Foundation Earth (2022)^105^ | *GL21* | *0* | *0* | *1* | *0* | *0* | *0* | *1* |
|  | Foundation Earth (2022)^106^ | *GL22* | *0* | *0* | *1* | *0* | *0* | *0* | *1* |
|  | Naked (2022)^107^ | *GL23* | *0* | *0* | *1* | *0* | *0* | *0* | *1* |
|  | Naked (2022)^108^ | *GL24* | *0* | *0* | *1* | *0* | *0* | *0* | *1* |
|  | Naked (2022)^109^ | *GL25* | *0* | *0* | *1* | *0* | *0* | *0* | *1* |
|  | Naked (2022)^110^ | *GL26* | *0* | *0* | *1* | *0* | *0* | *0* | *1* |
|  | Naked (2022)^111^ | *GL27* | *0* | *0* | *1* | *0* | *0* | *0* | *1* |
|  | Naked (2022)^112^ | *GL28* | *0* | *0* | *1* | *0* | *0* | *0* | *1* |
|  | Naked (2022)^113^ | *GL29* | *0* | *0* | *1* | *0* | *0* | *0* | *1* |
|  | Naked (2022)^114^ | *GL30* | *0* | *0* | *1* | *0* | *0* | *0* | *1* |
|  | Naked (2022)^115^ | *GL31* | *0* | *0* | *1* | *0* | *0* | *0* | *1* |
|  | Naked (2022)^116^ | *GL32* | *0* | *0* | *1* | *0* | *0* | *0* | *1* |
|  | DUG Drinks (2022)^117^ | *GL33* | *0* | *0* | *1* | *0* | *0* | *0* | *1* |
|  | Cammelbeeck & Rolvink (2017)^118^ | *GL34* | *1* | *1* | *1* | *1* | *1* | *0* | *5* |
|  | Goudie & Hughes (2022)^119^ | *GL35* | *1* | *1* | *0* | *1* | *1* | *0* | *4* |
|  | Kazer et al. (2022)^120^ | *GL36* | *1* | *1* | *1* | *0* | *0* | *0* | *3* |

#### Sensitivity analysis of nutrient and environmental outcomes on studies partially funded by the industry

**Table S13: Sensitivity analysis based on funding source (partially funded by the industry) of the percentage difference (Novel plant-based foods vs animal-based foods) for the nutrient content and environmental impacts.^a^**

| **Parameter (%)** | **Percentage difference in health/environmental outcomes**  **of NPBFs vs ABFs** | **Academic funder vs studies partially funded by the industry** |
| --- | --- | --- |
|  | **Studies partially funded by the industry (=11)** | **Wilcoxon Test** |
|  | **Median (IQR)** | **P value** |
| **Energy^b^** | -11.27  (-30.62 to 2.89) | <0.001 |
| **Fibre**^c^ | 733.33  (483.33 to 966.67) | <0.001 |
| **Saturated Fat^b^** | -84.46  (-88.69 to -57.61) | <0.001 |
| **Total Sugar** | 18.31  (-50.70 to 126.76) | 0.105 |
| **Sodium**^c^ | 58.87  (14.57 to 98.58) | <0.001 |
| **Greenhouse gas emissions**^c^ | -86.13  (-90.98 to -75.20) | 0.002 |
| **Land Use^b^** | -83.76  (-91.70 to -76.44) | 0.012 |
| **Water Use** | -56.43  (-84.83 to -41.79) | 0.447 |
| ^a^ The superscript ^b^ and ^c^ indicate the direction and dimension of the association:  ^b^ Industry funded studies show a more positive impact on health and environmental outcomes of their PB products (versus animal sourced foods) as compared to academically funded studies  ^c^ Industry funded studies show a less positive impact on health and environmental outcomes of their PB products (versus animal sourced foods) as compared to academically funded studies  *Abbreviations: IQR: Interquartile ranges; NPBFs: Novel plant-based foods; ABFs: Animal-based foods* | | |

# Supplementary references

1. Smart Protein Project. *Plant-based foods in Europe: How big is the market?* 2021. *Smart Protein Plant-based Food Sector Report*. Available at: <https://smartproteinproject.eu/plant-based-food-sector-report>.

2. Devlin E. This raises £15m in series B funding round to fuel ongoing rapid growth. Accessed April, 2023. Available at: <https://www.thegrocer.co.uk/fundraising/this-raises-15m-in-series-b-funding-round-to-fuel-ongoing-rapid-growth/676873.article>.

3. Ettinger J. Heura Saw a 260% Increase In International Vegan Meat Sales in 2022. Green Queen. Accessed March, 2023. Available at: <https://www.greenqueen.com.hk/heura-2022-international-vegan-meat-sales/>.

4. Datassential Research. *2023 Food Trends*. 2022. *FOODBYTES: Trend report*. Available at: <https://offers.datassential.com/2023-trends>.

5. Foster-Collins L. The death of vegan brands and the unlikely saviour of plant-based diets. Accessed June, 2023. Available at: <https://www.thegrocer.co.uk/plant-based/the-death-of-vegan-brands-and-saviour-of-plant-based-diets/680057.article?utm_source=Weekly%20Shop%20(The%20Grocer)&utm_medium=email&utm_campaign=2023-06-12&c=&cid=DM1080641&bid=132435027>.

6. Tatum M. Is Beyond Meat beyond hope? And what its slump says about the other plant-based players. The Grocer. Accessed March, 2023. Available at: <https://www.thegrocer.co.uk/plant-based/is-beyond-meat-beyond-hope-and-what-its-slump-says-about-the-other-plant-based-players/669272.article>.

7. Good Food Institute. *2022 State of the Industry Report: Plant-based meat, seafood, eggs, and dairy*. 2022. Available at:

8. Whitmee S, Haines A, Beyrer C, et al. Safeguarding human health in the Anthropocene epoch: report of The Rockefeller Foundation–Lancet Commission on planetary health. *The Lancet*. 2015;386(10007):1973-2028. doi:10.1016/s0140-6736(15)60901-1.

9. Jarmul S, Dangour AD, Green R, Liew Z, Haines A, Scheelbeek PF. Climate change mitigation through dietary change: a systematic review of empirical and modelling studies on the environmental footprints and health effects of 'sustainable diets'. *Environmental Research Letters*. 2020;15:123014. doi:10.1088/1748-9326/abc2f7.

10. Alae-Carew C, Green R, Stewart C, Cook B, Dangour AD, Scheelbeek PFD. The role of plant-based alternative foods in sustainable and healthy food systems: Consumption trends in the UK. *Science of Total Environment*. 2021:151041. doi:10.1016/j.scitotenv.2021.151041.

11. The World Bank. High income. Accessed June, 2022. Available at: <https://data.worldbank.org/income-level/high-income>.

12. Chang C-C, Lin C-J. LIBSVM: a library for support vector machines. *ACM Transactions on Intelligent Systems and Technology*. 2011;2(3):1-27. doi:10.1145/1961189.1961199.

13. Pedregosa F, Varoquaux G, Gramfort A, et al. Scikit-learn: Machine learning in Python. *The Journal of Machine Learning Research*. 2011;12:2825-2830.

14. Berrang-Ford L, Sietsma AJ, Callaghan M, et al. Systematic mapping of global research on climate and health: a machine learning review. *The Lancet Planetary Health*. 2021;5(8):e514-e525. doi:10.1016/s2542-5196(21)00179-0.

15. Critical Appraisal Skills Programme. CASP Checklist: CASP Randomised Controlled Trial Checklist. Accessed March, 2023. Available at: <https://casp-uk.net/casp-tools-checklists/>.

16. Renotte N. nicknochnack/Longform-Summarization-with-Hugging-Face. Accessed September, 2022. Available at: <https://github.com/nicknochnack/Longform-Summarization-with-Hugging-Face/blob/main/LongSummarization.ipynb>.

17. SACN & COT. *1st Meeting*. 2021. *Joint SACN/COT working group on plant-based drinks*. Available at: <https://app.box.com/s/l3kmzedxd6fo49a4p5eztbnaauf6917w>.

18. Global Burden of Disease Collaborative Network. Global Burden of Disease Results Tool: UK 2019. Global Health Data Exchange, Institute for Health Metrics and Evaluation. Accessed 26 April, 2022. Available at: <https://vizhub.healthdata.org/gbd-compare/>.

19. iPES-Food. *The Politics of Protein: Examining claims about the livestock, fish, ‘alternative proteins’ and sustainability*. 2022. Available at: <https://www.ipes-food.org/_img/upload/files/PoliticsOfProtein.pdf>.

20. Nelson G, Bogard J, Lividini K, et al. Income growth and climate change effects on global nutrition security to mid-century. *Nature Sustainability*. 2018;1(12):773-781. doi:10.1038/s41893-018-0192-z.

21. Leme ACB, Hou S, Fisberg RM, Fisberg M, Haines J. Adherence to Food-Based Dietary Guidelines: A Systemic Review of High-Income and Low- and Middle-Income Countries. *Nutrients*. 2021;13(3):1038. doi:10.3390/nu13031038.

22. Silva BQ, Smetana S. Review on milk substitutes from an environmental and nutritional point of view. *Applied Food Research*. 2022;2(1):100105. doi:10.1016/j.afres.2022.100105.

23. Edge MS, Garrett JL. The nutrition limitations of mimicking meat. *Cereal Foods World*. 2020;65(3)

24. Boeing H. Health effects of plant-based diets: From wholesome-mixed diets to extreme vegetarian diets. *Annals of Nutrition and Metabolism*. 2017;71(Supplement 2):89-89. 21st International Congress of Nutrition, ICN 2017. Buenos Aires Argentina. doi:<http://dx.doi.org/10.1159/000480486>.

25. PHE. McCance and Widdowson’s The Composition of Foods Integrated Dataset (CoFID) 2021. Accessed May, 2022. Available at: <https://www.gov.uk/government/publications/composition-of-foods-integrated-dataset-cofid>.

26. Poore J, Nemecek T. Reducing food’s environmental impacts through producers and consumers. *Science*. 2018;360(6392):987-992. doi:10.1126/science.aaq0216.

27. RStudio Team. RStudio: Integrated Development Environment for R. RStudio. Available at: <http://www.rstudio.com/>.

28. Harrington RA, Adhikari V, Rayner M, Scarborough P. Nutrient composition databases in the age of big data: foodDB, a comprehensive, real-time database infrastructure. *BMJ Open*. 2019;9(6):e026652. doi:10.1136/bmjopen-2018-026652.

29. Ridoutt B, Anastasiou K, Baird D, Garcia Navarro J, Hendrie G. Cropland Footprints of Australian Dietary Choices. *Nutrients*. 2020;12(5):1212.

30. Vogelsang-O'Dwyer M, Sahin AW, Zannini E, Arendt EK. Physicochemical and nutritional properties of high protein emulsion-type lupin-based model milk alternatives: effect of protein source and homogenization pressure. *Journal of the Science of Food and Agriculture*. 2021;102(12):5086-5097.

31. Pontonio E, Raho S, Dingeo C, Centrone D, Carofiglio VE, Rizzello CG. Nutritional, Functional, and Technological Characterization of a Novel Gluten- and Lactose-Free Yogurt-Style Snack Produced With Selected Lactic Acid Bacteria and Leguminosae Flours. *Frontiers in Microbiology*. 2020;11:1664.

32. Ma W, He X, Braverman L. Iodine Content in Milk Alternatives. *Thyroid*. 2016;26(9):1308-1310.

33. Richard C, Lewis ED, Zhao YY, et al. Measurement of the total choline content in 48 commercial dairy products or dairy alternatives. *Journal of Food Composition and Analysis*. 2016;45:1-8.

34. Cole E, Goeler-Slough N, Cox A, Nolden A. Examination of the nutritional composition of alternative beef burgers available in the United States. *International Journal of Food Sciences and Nutrition*. 2022;73(4):425-432. doi:10.1080/09637486.2021.2010035.

35. Martinez-Padilla E, Li K, Blok Frandsen H, Skejovic Joehnke M, Vargas-Bello-Perez E, Lykke Petersen I. In Vitro Protein Digestibility and Fatty Acid Profile of Commercial Plant-Based Milk Alternatives. *Foods*. 2020;9(12):1784.

36. Collard KM, McCormick DP. A Nutritional Comparison of Cow's Milk and Alternative Milk Products. *Academic Pediatrics*. 2020;21(6):1067-1069.

37. Zhang YY, Hughes J, Grafenauer S. Got Mylk? The Emerging Role of Australian Plant-Based Milk Alternatives as A Cow's Milk Substitute. *Nutrients*. 2020;12(5):1254.

38. Isidro V. The nutritional limitations of plant-based beverages in infancy and childhood. *Nutrición Hospitalaria*. 2017;34(5):1205-1214.

39. Jeske S, Zannini E, Arendt EK. Evaluation of Physicochemical and Glycaemic Properties of Commercial Plant-Based Milk Substitutes. *Plant Foods for Human Nutrition*. 2017;72(1):26-33.

40. Sumner O, Burbridge L. Plant-based milks: the dental perspective. *British Dental Journal*. 2020;8(6):16-23.

41. Toribio-Mateas MA, Bester A, Klimenko N. Impact of Plant-Based Meat Alternatives on the Gut Microbiota of Consumers: A Real-World Study. *Foods*. 2021;10(9):2040. doi:10.3390/foods10092040.

42. Dineva M, Rayman MP, Bath SC. Iodine status of consumers of milk-alternative drinks v. cows' milk: data from the UK National Diet and Nutrition Survey. *The British Journal of Nutrition*. 2021;126(1):28-36.

43. Al-Saedi N, Agarwal M, Islam S, Ren Y-L. Study on the Correlation between the Protein Profile of Lupin Milk and Its Cheese Production Compared with Cow's Milk. *Molecules*. 2021;26(8):2395.

44. Seves SM, Verkaik-Kloosterman J, Biesbroek S, Temme EH. Are more environmentally sustainable diets with less meat and dairy nutritionally adequate? *Public Health Nutrition*. 2017;20(11):2050-2062.

45. Sun LJ, Tan KWJ, Siow PC, Henry CJ. Soya milk exerts different effects on plasma amino acid responses and incretin hormone secretion compared with cows' milk in healthy, young men. *British Journal of Nutrition*. 2016;116(7):1216-1221.

46. Angelino D, Rosi A, Vici G, et al. Nutritional Quality of Plant-Based Drinks Sold in Italy: The Food Labelling of Italian Products (FLIP) Study. *Foods*. 2020;9(5):682.

47. Szparaga A, Tabor S, Kocira S, et al. Survivability of probiotic bacteria in model systems of non-fermented and fermented coconut and hemp milks. *Sustainability*. 2019;11(21):6093.

48. Tangyu M, Fritz M, Aragao-Borner R, et al. Genome-based selection and application of food-grade microbes for chickpea milk fermentation towards increased L-lysine content, elimination of indigestible sugars, and improved flavour. *Microbial Cell Factories*. 2021;20(1):1-21.

49. Winans KS, Kendall A, Marvinney E, Macadam-Somer I, Geyer R. Life cycle assessment of California unsweetened almond milk. *International Journal of Life Cycle Assessment*. 2020;25(3):577-587.

50. Grant CA, Hicks AL. Comparative life cycle assessment of milk and plant-based alternatives. *Environmental Engineering Science*. 2018;35(11):1235-1247.

51. Boeck T, Sahin AW, Zannini E, Arendt EK. Nutritional properties and health aspects of pulses and their use in plant-based yogurt alternatives. *Comprehensive Reviews in Food Science and Food Safety*. 2021;20(4):3858-3880.

52. Roos E, Patel M, Spangberg J. Producing oat drink or cow's milk on a Swedish farm - Environmental impacts considering the service of grazing, the opportunity cost of land and the demand for beef and protein. *Agricultural Systems*. 2016;142:23-32.

53. Smetana S, Profeta A, Voigt R, Kircher C, Heinz V. Meat substitution in burgers: nutritional scoring, sensorial testing, and Life Cycle Assessment. *Future Foods*. 2021;4:100042. doi:10.1016/j.fufo.2021.100042.

54. Liu H, Balamurugan S, Shao S, He J. Fatty acids and volatile flavor compounds in commercial plant-based burgers. *Journal of Food Science*. 2021;86(2):293-305.

55. van Vliet S, Bain JR, Muehlbauer MJ, et al. A metabolomics comparison of plant-based meat and grass-fed meat indicates large nutritional differences despite comparable Nutrition Facts panels. *Scientific Reports*. 2021;11(1):13828.

56. Crimarco A, Springfield S, Petlura C, et al. A randomized crossover trial on the effect of plant-based compared with animal-based meat on trimethylamine-N-oxide and cardiovascular disease risk factors in generally healthy adults: Study With Appetizing Plantfood-Meat Eating Alternative Trial (SWAP-MEAT). *The American Journal of Clinical Nutrition*. 2020;112(5):1188-1199.

57. Saget S, Costa M, Santos CS, et al. Substitution of beef with pea protein reduces the environmental footprint of meat balls whilst supporting health and climate stabilisation goals. *Journal of Cleaner Production*. 2021;297:126447.

58. Mejia M, Fresan U, Oda K, Sabate J, Harwatt H, Uriegas-Mejia G. Life Cycle Assessment of the Production of a Large Variety of Meat Analogs by Three Diverse Factories. *Journal of Hunger and Environmental Nutrition*. 2020;15(5):699-711.

59. Detzel A, Kruger M, Busch M, et al. Life cycle assessment of animal-based foods and plant-based protein-rich alternatives: An environmental perspective. *Journal of the Science of Food and Agriculture*. 2021;102(12):5098-5110.

60. Mertens E, Biesbroek S, Dofkova M, et al. Potential impact of meat replacers on nutrient quality and greenhouse gas emissions of diets in four European countries. *Sustainability*. 2020;12(17):6838.

61. Heusala H, Sinkko T, Mogensen L, Knudson MT. Carbon footprint and land use of food products containing oat protein concentrate. *Journal of Cleaner Production*. 2020;276:122938.

62. Goldstein B, Moses R, Sammons N, Birkved M. Potential to curb the environmental burdens of American beef consumption using a novel plant-based beef substitute. *PloS one*. 2017;12(12):e0189029.

63. Fresan U, Mejia MA, Craig WJ, Jaceldo-Siegl K, Sabate J. Meat analogs from different protein sources: a comparison of their sustainability and nutritional content. *Sustainability*. 2019;11(12):3231.

64. Saerens W, Smetana S, Van Campenhout L, Lammers V, Heinz V. Life cycle assessment of burger patties produced with extruded meat substitutes. *Journal of Cleaner Production*. 2021;306:127177.

65. Farsi DN, Uthumange D, Munoz Munoz J, Commane DM. The nutritional impact of replacing dietary meat with meat alternatives in the UK: a modelling analysis using nationally representative data. *The British Journal of Nutrition*. 2021;127(11):1-31.

66. De Marchi M, Costa A, Pozza M, Goi A, Manuelian CL. Detailed characterization of plant-based burgers. *Scientific Reports*. 2021;11(1):2049.

67. Kouw IWK, Pinckaers PJM, Le Bourgot C, et al. Ingestion of an ample amount of meat substitute based on a lysine-enriched, plant-based protein blend stimulates postprandial muscle protein synthesis to a similar extent as an isonitrogenous amount of chicken in healthy, young men. *British Journal of Nutrition*. 2021;128(10):1955-1965. doi:10.1017/S0007114521004906.

68. Curtain F, Grafenauer S. Plant-Based Meat Substitutes in the Flexitarian Age: An Audit of Products on Supermarket Shelves. *Nutrients*. 2019;11(11):2603.

69. Bottin JH, Swann JR, Cropp E, et al. Mycoprotein reduces energy intake and postprandial insulin release without altering glucagon-like peptide-1 and peptide tyrosine-tyrosine concentrations in healthy overweight and obese adults: a randomised-controlled trial. *The British Journal of Nutrition*. 2016;116(2):360-74.

70. Coelho MOC, Monteyne AJ, Dirks ML, Finnigan TJA, Stephens FB, Wall BT. Daily mycoprotein consumption for 1 week does not affect insulin sensitivity or glycaemic control but modulates the plasma lipidome in healthy adults: a randomised controlled trial. *British Journal of Nutrition*. 2021;125(2):147-160.

71. Fresan U, Rippin H. Nutritional Quality of Plant-Based Cheese Available in Spanish Supermarkets: How Do They Compare to Dairy Cheese? *Nutrients*. 2021;13(9):3291. doi:10.3390/nu13093291.

72. Clegg ME, Tarrado Ribes A, Reynolds R, Kliem K, Stergiadis S. A comparative assessment of the nutritional composition of dairy and plant-based dairy alternatives available for sale in the UK and the implications for consumers' dietary intakes. *Food Research International*. 2021;148doi:10.1016/j.foodres.2021.110586.

73. Alessandrini R, Brown MK, Pombo-Rodrigues S, Bhageerutty S, He FJ, MacGregor GA. Nutritional Quality of Plant-Based Meat Products Available in the UK: A Cross-Sectional Survey. *Nutrients*. 2021;13(12):4225. doi:10.3390/nu13124225.

74. Bianchi F, Stewart C, Astbury NM, Cook B, Aveyard P, Jebb SA. Replacing meat with alternative plant-based products (RE-MAP): a randomized controlled trial of a multicomponent behavioral intervention to reduce meat consumption. *The American Journal of Clinical Nutrition*. Dec 27 2021;115(5):1357-1366. doi:10.1093/ajcn/nqab414.

75. Fresan U, Marrin D, Mejia M, Sabaté J. Water Footprint of Meat Analogs: Selected Indicators According to Life Cycle Assessment. *Water*. 2019;11(4):728. doi:10.3390/w11040728.

76. Bryngelsson S, Moshtaghian H, Bianchi M, Hallstrom E. Nutritional assessment of plant-based meat analogues on the Swedish market. *International Journal of Food Sciences and Nutrition*. 2022;73(7):889-901. doi:10.1080/09637486.2022.2078286.

77. Marques M, Correig E, Capdevila E, et al. Essential and Non-essential Trace Elements in Milks and Plant-Based Drinks. *Biological Trace Element Research*. 2021:1-10. doi:10.1007/s12011-021-03021-5.

78. Saget S, Porto Costa M, Santos CS, Vasconcelos M, Styles D, Williams M. Comparative life cycle assessment of plant and beef-based patties, including carbon opportunity costs. *Sustainable Production and Consumption*. 2021;28:936-952. doi:10.1016/j.spc.2021.07.017.

79. Coluccia B, Agnusdei G, De Leo F, Vecchio Y, La Fata CM, Miglietta PP. Assessing the carbon footprint across the supply chain: Cow milk vs soy drink. *Science of the Total Environment*. 2022;806(Pt. 3):151200–151200. doi:10.1016/j.scitotenv.2021.151200.

80. Boukid F, Lamri M, Dar BN, Garron M, Castellari M. Vegan Alternatives to Processed Cheese and Yogurt Launched in the European Market during 2020: A Nutritional Challenge? *Foods*. 2021;10(11):2782. doi:10.3390/foods10112782.

81. Pointke M, Pawelzik E. Plant-Based Alternative Products: Are They Healthy Alternatives? Micro- and Macronutrients and Nutritional Scoring. *Nutrients*. 2022;14(3):601. doi:10.3390/nu14030601.

82. Shen P, Walker GD, Yuan Y, et al. Effects of soy and bovine milk beverages on enamel mineral content in a randomized, double-blind in situ clinical study. *Journal of Dentistry*. 2019;88:103160. doi:10.1016/j.jdent.2019.06.007.

83. Farsi DN, Gallegos JL, Koutsidis G, et al. Substituting meat for mycoprotein reduces genotoxicity and increases the abundance of beneficial microbes in the gut: Mycomeat, a randomised crossover control trial. *European Journal of Nutrition*. 2023;62(3):1479-1492. doi:10.1007/s00394-023-03088-x.

84. Melville H, Shahid M, Gaines A, et al. The nutritional profile of plant-based meat analogues available for sale in Australia. *Nutrition & Dietetics*. 2023;80(2):211-222. doi:10.1111/1747-0080.12793.

85. Quantis. *Violife 100% vegan alternative to cheese vs. dairy cheese in Europe, UK, US and Canada*. 2022. *Life Cycle Assessment Technical Summary*. Available at: <https://violifefoods.com/wp-content/uploads/2022/01/Violife_TechnicalSummary_2022-01-25-UPDATED.pdf>.

86. CarbonCloud. *Climate footprint for Enriched ambient oat drink, Sweden*. 2020. Available at: <https://a.storyblok.com/f/107921/x/5a334aa2dc/02-enriched-ambient-oat-drink_d_sweden-2.pdf>.

87. CarbonCloud. *The climate footprint of Barista oat drink, Sweden/Finland Oatly*. 2021. Available at: <https://a.storyblok.com/f/107921/x/d3e35308bb/04-barista-finland-1.pdf>.

88. CarbonCloud. *The climate footprint of Barista oat drink, UK Oatly*. 2021. Available at: <https://a.storyblok.com/f/107921/x/13d3f633d4/barista-uk_e.pdf>.

89. Alpro. *Feeding Our Future with Plants: Leading the way to a planetary plate by 2025*. 2020. *Sustainability Report: Vision 2020*. Available at: <https://downloads.ctfassets.net/s64jgdakkdiy/4Obsp45T6ghqRCy60SbXjQ/183c5f968ea9f49a75447f4f58eace11/200916_Alpro_Sustainability_Report_Vision_2020.pdf>.

90. Ritchie H. Dairy vs. plant-based milk: what are the environmental impacts? Accessed May, 2022. Available at: <https://ourworldindata.org/environmental-impact-milks>.

91. Khan S, Loyola C, Dettling J, Hester J, Moses R. *Comparative Environmental LCA of the Impossible Burger with Conventional Ground Beef Burger* 2019. Available at: <https://assets.ctfassets.net/hhv516v5f7sj/4exF7Ex74UoYku640WSF3t/cc213b148ee80fa2d8062e430012ec56/Impossible_foods_comparative_LCA.pdf>.

92. Heller MC, Keoleian GA. *Beyond Meat’s Beyond Burger Life. Cycle Assessment: A detailed comparison between a plant- based and an animal-based protein source*. 2018:1-38. *CSS Report*. Available at: <https://css.umich.edu/sites/default/files/publication/CSS18-10.pdf>.

93. Dettling J, Tu Q, Faist M, DelDuce A, Mandlebaum S. *A comparative Life Cycle Assessment of plant-based foods and meat foods*. 2016. Available at: <https://www.morningstarfarms.com/content/dam/NorthAmerica/morningstarfarms/pdf/MSFPlantBasedLCAReport_2016-04-10_Final.pdf>.

94. Reinhardt G, Gärtner S, Wagner T. *Ökologische Fußabdrücke von Lebensmitteln und Gerichten in Deutschland*. 2020. Available at:

95. Kazer J, Orfanos G, Gallop C. *Quorn Footprint Comparison Report*. 2021. Available at: <https://www.quorn.co.uk/assets/files/content/Carbon-Trust-Comparison-Report-2021.pdf>.

96. THIS Co. Why THIS? Accessed May, 2022. Available at: <https://this.co/why/>.

97. Foundation Earth. Mighty Barista. Accessed May, 2022. Available at: <https://www.foundation-earth.org/product/M00M/>.

98. Foundation Earth. Protein Oat MIGHTY M.LK - 1L. Accessed May, 2022. Available at: <https://www.foundation-earth.org/product/MP07/>.

99. Foundation Earth. Unsweetened MIGHTY M.LK - 1L. Accessed May, 2022. Available at: <https://www.foundation-earth.org/product/M2PM/>.

100. Foundation Earth. Original MIGHTY M.LK 1L. Accessed May, 2022. Available at: <https://www.foundation-earth.org/product/M75M/>.

101. Foundation Earth. MIGHTY M.lkology Semi. Accessed May, 2022. Available at: <https://www.foundation-earth.org/product/MP60/>.

102. Foundation Earth. MIGHTY M.lkology Whole. Accessed May, 2022. Available at: <https://www.foundation-earth.org/product/MP05/>.

103. Foundation Earth. Jack & Bry Jackfruit Beef Burgers 200g. Accessed May, 2022. Available at: <https://www.foundation-earth.org/product/JB22/>.

104. Foundation Earth. VFC Bites Original. Accessed May, 2022. Available at: <https://www.foundation-earth.org/product/V6UV/>.

105. Foundation Earth. VFC Fillets Original. Accessed May, 2022. Available at: <https://www.foundation-earth.org/product/V6HV/>.

106. Foundation Earth. VFC Poppers Original. Accessed May, 2022. Available at: <https://www.foundation-earth.org/product/V6HV/>.

107. Naked. Better Naked Evolution Meatballs. Accessed May, 2022. Available at: <https://www.betternaked.com/products/better-naked-evolution-meatballs/>.

108. Naked. Better Naked Spicy Bean Burgers – Veg & Proud. Accessed May, 2022. Available at: <https://www.betternaked.com/products/better-naked-spicy-bean-burgers-veg-proud/>.

109. Naked. Better Naked Garlic Kyivs – Without The Cluck. Accessed May, 2022. Available at: <https://www.betternaked.com/products/better-naked-garlic-kyivs-without-the-cluck/>.

110. Naked. Better Naked Nuggets – Without The Cluck. Accessed May, 2022. Available at: <https://www.betternaked.com/products/better-naked-nuggets-without-the-cluck/>.

111. Naked. Better Naked Lightly Battered Goujons – Without The Splash. Accessed May, 2022. Available at: <https://www.betternaked.com/products/better-naked-lightly-battered-goujons-without-the-splash/>.

112. Naked. Better Naked Southern Fried Goujons – Without The Cluck. Accessed April, 2023. Available at: <https://www.foundation-earth.org/product/FB46/>.

113. Naked. Better Naked Evolution Burger. Accessed May, 2022. Available at: <https://www.betternaked.com/products/better-naked-evolution-burger/>.

114. Naked. Better Naked Evolution Sausages. Accessed May, 2022. Available at: <https://www.betternaked.com/products/better-naked-evolution-sausages/>.

115. Naked. Better Naked Evolution Mince. Accessed May, 2022. Available at: <https://www.betternaked.com/products/better-naked-evolution-mince/>.

116. Naked. Tikka Pieces. Accessed May, 2022. Available at: <https://www.betternaked.com/products/tikka-pieces/>.

117. DUG Drinks. Super-sustainability. Accessed June, 2022. Available at: <https://dugdrinks.com/sustainability/>.

118. Cammelbeeck T, Rolvink R. *Onderzoek Alternatieven Voor Vlees: Wat Zijn Alternatieven Voor Wie Minder Vlees Wil Eten?* 2017. Available at: <https://www.consumentenbond.nl/binaries/content/assets/cbhippowebsite/tests/gezond-eten/rapport-vleesvervangers-v4.pdf>.

119. Goudie S, Hughes I. *The Broken Plate Report 2022*. 2022. Available at: <https://foodfoundation.org.uk/publication/broken-plate-2022>.

120. Kazer J, Orfanos G, Gallop C. *Quorn Footprint Comparison Report*. 2022. Available at: <https://www.quorn.co.uk/assets/files/content/Carbon-Trust-Comparison-Report-2022.pdf>.

121. Cherta-Murillo A, Frost GS. The association of mycoprotein-based food consumption with diet quality, energy intake and non-communicable diseases' risk in the UK adult population using the National Diet and Nutrition Survey (NDNS) years 2008/2009-2016/2017: a cross-sectional study. *The British Journal of Nutrition*. 2021;127(11):1685-1694.
