# Supplementary figures and images for: Mapping the evidence of novel plant-based foods: a systematic review of nutritional, health, and environmental impacts in high-income countries
Source: Nutr Rev. 2024 Apr 25;83(7):e1626–46. doi: 10.1093/nutrit/nuae031 (PMC12166169; doi:10.1093/nutrit/nuae031)

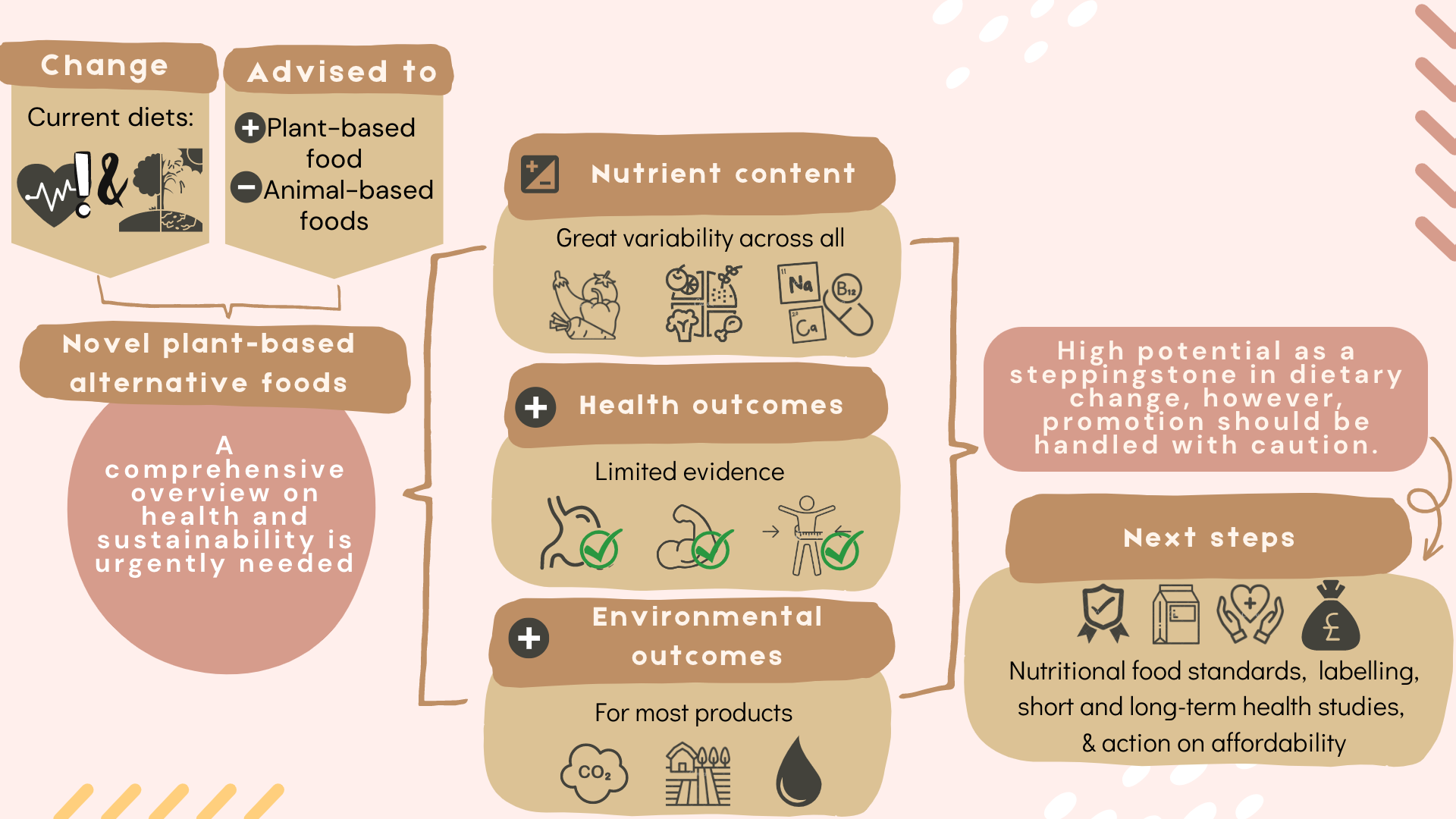

Supplement: nuae031_Supplementary_Data [file nuae031_supplementary_data.zip › nuae031_Supplementary_Data/graphic_abstract.tiff]
